# Supplementary material for: Care bundle to reduce readmission in patients with heart failure: a modified Delphi consensus panel in Argentina
Source: BMJ Open. 2020 Dec 29;10(12):e040028. doi: 10.1136/bmjopen-2020-040028 (PMC7778781; doi:10.1136/bmjopen-2020-040028)

Supplemental Table 1. Interventions and criteria pondering results

	1. Priority	2. Intended effects	3. Unintended effects	4. Balance	5. Quality of evidence on effects	6.Values and preferences	7. Resource required	8. Quality of evidence on resources	9. Cost-effectiveness	10. Equity	11. Acceptability	12. Feasibility	13. Measurability	Score	
Weighting factor	1.00	1.00	0.89	0.89	0.89	0.78	0.78	0.78	0.89	0.89	0.78	0.89	0.78		
Recommendation															
ACE-inhibitors	9.00	9.00	2.67	7.11	8.00	6.22	7.00	6.22	8.00	7.11	7.00	8.00	7.00	**96.00**	7.38
Lab	9.00	9.00	1.78	7.11	7.11	6.22	7.00	5.44	8.00	7.56	7.00	8.00	7.00	**95.67**	7.36
Blood Pressure	8.00	8.00	2.67	7.11	7.11	6.22	7.00	6.22	8.00	7.11	6.61	8.00	6.61	**92.33**	7.10
Dairy weight	8.50	8.50	1.78	8.00	6.22	6.22	6.22	5.44	7.11	7.56	6.22	7.11	6.22	**90.56**	6.97
Beta block	9.00	9.00	2.67	7.11	8.00	6.22	6.22	2.72	7.11	7.11	7.00	7.56	7.00	**90.39**	6.95
Sodium	8.50	8.00	1.78	7.11	6.22	5.44	7.00	5.44	7.56	7.56	6.22	7.56	5.44	**89.28**	6.87
Follow-up	9.00	9.00	1.78	7.56	6.22	6.22	4.67	5.44	7.11	7.11	6.22	7.11	6.22	**89.11**	6.85
Smoking cesation	9.00	9.00	2.22	7.11	7.11	4.67	5.44	5.44	7.11	7.11	6.22	6.22	5.44	**86.67**	6.67
Anti mineralocorticoids	8.00	8.00	4.44	6.22	8.00	5.44	6.22	6.22	7.11	7.11	6.22	7.11	6.22	**86.44**	6.65
Diuretics	9.00	8.00	4.44	6.67	5.33	5.44	6.61	5.44	7.11	6.67	6.22	8.00	7.00	**86.06**	6.62
Sacubitril	8.00	9.00	2.67	7.11	8.00	6.22	4.67	6.22	6.22	5.33	5.44	6.22	7.00	**85.78**	6.60
Echocardiography	8.00	8.00	0.89	7.11	6.22	6.22	3.89	4.67	7.11	6.22	6.22	6.22	6.61	**84.61**	6.51
Descompensation Cause	9.00	8.50	4.44	7.11	6.22	6.22	5.06	5.44	7.11	6.67	6.22	6.22	6.22	**84.56**	6.50
Telephone tracking	8.00	8.00	1.78	7.11	6.22	6.22	4.28	5.44	6.67	6.22	5.44	5.33	6.22	**82.39**	6.34
Flu vaccine	7.00	7.00	2.22	6.67	5.33	5.44	5.44	4.67	7.11	7.11	6.22	7.11	6.22	**82.11**	6.32
Weightloss	7.00	7.00	2.67	6.22	5.33	4.67	5.83	4.67	6.22	5.78	5.44	6.22	5.44	**76.17**	5.86
Exercicse	7.00	7.00	7.11	7.11	6.22	5.44	6.22	3.89	6.22	7.11	5.44	6.22	5.44	**75.22**	5.79
Sexual activity	7.00	7.00	4.00	6.22	4.44	5.44	6.22	4.67	6.22	6.22	4.67	6.22	4.67	**74.00**	5.69
ProBNP	7.00	7.00	1.78	6.22	6.22	5.44	3.89	5.44	4.44	3.56	3.89	4.44	6.22	**71.00**	5.46
Lung echography	6.00	6.00	1.78	6.67	5.33	5.06	3.89	4.67	4.89	4.44	5.06	5.33	6.22	**70.78**	5.44
Psychologist	6.00	7.00	3.56	6.22	5.33	4.67	4.67	4.67	6.22	5.33	4.67	4.44	4.67	**69.33**	5.33
Ferritin	7.00	3.00	2.67	6.22	6.22	4.67	3.89	3.89	5.78	4.44	4.67	5.33	5.83	**67.28**	5.18
Ivabrav	6.00	6.00	2.67	6.22	6.22	4.67	5.44	4.67	5.33	4.44	4.28	6.22	5.83	**67.22**	5.17
Apnea	7.00	7.00	3.56	6.22	5.78	4.67	2.33	4.67	5.33	3.56	4.67	4.00	5.44	**66.11**	5.09
Resynchronizer	6.00	4.00	3.56	6.22	6.67	4.67	1.56	5.44	5.33	2.67	4.67	4.00	6.22	**62.89**	4.84
Wireless HF monitor	3.00	6.00	2.67	4.44	5.33	2.33	0.78	4.28	4.44	1.78	2.72	1.78	5.44	**48.67**	3.74

Supplemental Annex 1. Search strategies

Search	Query	Items found
#17	Search **(#15 AND #16)**	1046
#16	Search **((Systematic Review[sb] OR Systematic Review[tiab] OR Meta-Analysis[pt] OR Meta-Analys*[tiab] OR "Cochrane Database Syst Rev"[ta] OR Metaanalysis[tiab] OR Metanalysis[tiab] OR Sysrev_Methods[sb] OR (MEDLINE[tiab] AND Cochrane[tiab]) OR Guideline[pt] OR Practice Guideline[pt] OR Guideline*[ti] OR Guide Line*[tiab] OR Consensus[tiab] OR Recommendation*[ti] OR Randomized Controlled Trial[pt] OR Random*[ti] OR Controlled Trial*[tiab] OR Control Trial*[tiab] OR Technology Assessment, Biomedical[Mesh] OR Technology Assessment[tiab] OR Technology Appraisal[tiab] OR HTA[tiab] OR Overview[ti] OR (Review[ti] AND Literature[ti])))**	1277580
#15	Search **(#8 AND #14)**	5959
#14	Search **(#9 OR #10 OR #11 OR #12 OR #13)**	37520
#13	Search **Re-Hospitaliz*[tiab]**	1206
#12	Search **Rehospitaliz*[tiab]**	5251
#11	Search **Readmitted[tiab]**	6511
#10	Search **Readmission*[tiab]**	24703
#9	Search **Patient Readmission[Mesh]**	15507
#8	Search **(#1 OR #2 OR #3 OR #4 OR #5 OR #6 OR #7)**	204191
#7	Search **Heart Failure[tiab]**	163364
#6	Search **Myocardial Decompensat*[tiab]**	33
#5	Search **Myocardial Failure[tiab]**	757
#4	Search **Cardiac Decompensat*[tiab]**	1156
#3	Search **Heart Decompensat*[tiab]**	122
#2	Search **Cardiac Failure[tiab]**	11760
#1	Search **Heart Failure[MeSH]**	115775

No.	Query	Results
#19	#17 AND #18	**80**
#18	(((('systematic review':ti,ab OR 'meta analysis (topic)':pt OR 'meta analysis':ti,ab OR cochrane:jt OR metaanalysis:ti,ab OR metanalysis.ti,ab. OR (medline:ab AND cochrane:ab) OR practice) AND guideline:pt OR guideline*:ti OR 'guide lines':ti,ab OR consensus:ti,ab OR recommendation*:ti OR randomized) AND controlled AND trial:pt OR random*:ti OR controlled) AND trial*:ti,ab OR 'control trial':ti,ab OR biomedical) AND technology AND 'assessment'/exp OR 'technology assessment':ti,ab OR 'technology appraisal':ti,ab OR hta:ti,ab OR overview:ti OR (review:ti AND literature:ti)	**194196**

No.	Query	Results
#17	#8 AND #16	**16307**
#16	#9 OR #10 OR #11 OR #12 OR #13 OR #14 OR #15	**75899**
#15	're hospitaliz*':ti,ab	**3444**
#14	're hospitalis*':ti,ab	**486**
#13	rehospitalis*:ti,ab	**1208**
#12	rehospitaliz*:ti,ab	**8201**
#11	readmitted:ti,ab	**13183**
#10	readmission*:ti,ab	**45071**
#9	'hospital readmission'/exp	**56483**
#8	#1 OR #2 OR #3 OR #4 OR #5 OR #6 OR #7	**536876**
#7	'heart failure':ti,ab	**259617**
#6	(myocardial NEAR/1 decompensat*):ti,ab	**57**
#5	'myocardial failure':ti,ab	**934**
#4	(cardiac NEAR/1 decompensat*):ti,ab	**1917**
#3	(heart NEAR/1 decompensat*):ti,ab	**6759**
#2	'cardiac failure':ti,ab	**16881**
#1	'heart failure'/exp	**491308**

CINAHL (EBSCO) 26-09-2019

#	Query	Results
S22	S20 AND S21	610
S21	(MH "Systematic Review") OR (MH "Cochrane Library") OR TI Meta-Analysis OR AB Meta-Analysis OR TI Metaanalysis OR AB Metaanalysis OR (AB Medline AND AB Cochrane) OR TI Guideline* OR AB Guideline* OR TI “Guide Lines” OR AB “Guide Lines” OR TI Consensus OR TI Recommendation* OR TI Random* OR AB Random* OR TI “Controlled Trial” OR AB “Controlled Trial” OR TI “Technology Assessment” OR AB “Technology Assessment” OR TI “Technology Appraisal” OR AB “Technology Appraisal” OR TI HTA OR AB HTA OR TI Overview OR (TI Review AND TI Literature)	547,021
S20	S13 AND S19	2,297
S19	S14 OR S15 OR S16 OR S17 OR S18	13,923
S18	TI Re-Hospitali* OR AB Re-Hospitali*	504
S17	TI Rehospitali* OR AB Rehospitali*	2,196
S16	TI Readmitted OR AB Readmitted	2,020

S15	TI Readmission* OR AB Readmission*	12,455
S14	(MH "Readmission")	11,566
S13	S1 OR S2 OR S3 OR S4 OR S5 OR S6 OR S7 OR S8 OR S9 OR S10 OR S11 OR S12	35,130
S12	TI "Heart Failure" OR AB "Heart Failure"	44,414
S11	TI "Myocardial Decompensated" OR AB "Myocardial Decompensated"	173
S10	TI "Myocardial Decompensation" OR AB "Myocardial Decompensation"	7
S9	TI "Myocardial Failure" OR AB "Myocardial Failure"	44
S8	TI "Cardiac Decompensated" OR AB "Cardiac Decompensated"	386
S7	TI "Cardiac Decompensated" OR AB "Cardiac Decompensated"	0
S6	TI "Cardiac Decompensation" OR AB "Cardiac Decompensation"	98
S5	TI "Heart Decompensated" OR AB "Heart Decompensated"	1,597
S4	TI "Heart Decompensated" OR AB "Heart Decompensated"	0
S3	TI "Heart Decompensation" OR AB "Heart Decompensation"	8
S2	TI "Cardiac Failure" OR AB "Cardiac Failure"	1,227
S1	(MH "Heart Failure+")	36,791

Global Health (OVID) 26-09-2019

☐	# ▲	**Searches**	**Results**
☐	1	Cardiac Failure.ti,ab.	1058
☐	2	Heart Decompensat*.ti,ab.	1
☐	3	Cardiac Decompensat*.ti,ab.	38
☐	4	Myocardial Failure.ti,ab.	61
☐	5	Myocardial Decompensat*.ti,ab.	0
☐	6	Heart Failure.ti,ab.	9089
☐	7	or/1-6	10064
☐	8	Readmission*.ti,ab.	2050
☐	9	Readmitted.ti,ab.	1113
☐	10	Rehospitaliz*.ti,ab.	310
☐	11	Re-Hospitaliz*.ti,ab.	121
☐	12	or/8-11	3203
☐	13	7 and 8	158

Estrategias Heart Failure Bundles

PubMed 24-09-2019

	Query	Items found
#14	**(#13 NOT Branch[tiab])**	319
#13	**(#4 AND #12)**	2109
#12	**(#5 OR #6 OR #7 OR #8 OR #9 OR #10 OR #11)**	204129
#11	**Heart Failure[tiab]**	163313
#10	**Myocardial Decompensat*[tiab]**	33
#9	**Myocardial Failure[tiab]**	757
#8	**Cardiac Decompensat*[tiab]**	1155
#7	**Heart Decompensat*[tiab]**	122
#6	**Cardiac Failure[tiab]**	11758
#5	**Heart Failure[MeSH]**	115710
#4	**(#1 OR #2 OR #3)**	62341
#3	**Bundles[tiab]**	27534
#2	**Bundle[tiab]**	38958
#1	**Patient Care Bundles[MeSH]**	662

EMBase (Elsevier) 24-09-2019

No.	Query	Results
#14	#13 NOT branch:ti,ab	**1009**
#13	#4 AND #12	**5474**
#12	#5 OR #6 OR #7 OR #8 OR #9 OR #10 OR #11	**536741**
#11	(myocardial NEAR/1 decompensat*):ti,ab	**57**
#10	'myocardial failure':ti,ab	**934**
#9	(cardiac NEAR/1 decompensat*):ti,ab	**1917**
#8	(heart NEAR/1 decompensat*):ti,ab	**6757**
#7	'cardiac failure':ti,ab	**16879**
#6	'heart failure':ti,ab	**259558**
#5	'heart failure'/exp	**491184**
#4	#1 OR #2 OR #3	**78092**
#3	bundles:ti,ab	**31971**
#2	bundle:ti,ab	**51066**
#1	'care bundle'/exp	**1150**

CINAHL (EBSCO) 24-09-2019

#	Query	Results
S13	S12 NOT (TI Branch OR AB Branch)	74
S12	S3 AND S11	548
S11	S4 OR S5 OR S6 OR S7 OR S8 OR S9 OR S10	34,961
S10	TI "Heart Failure" OR AB "Heart Failure"	44,368
S9	TI "Myocardial Decompensation" OR AB "Myocardial Decompensation"	7
S8	TI "Myocardial Failure" OR AB "Myocardial Failure"	44
S7	TI "Cardiac Decompensation" OR AB "Cardiac Decompensation"	98
S6	(TI "Heart Decompensation" OR AB "Heart Decompensation") OR (TI "Heart Decompensated" OR "Heart Decompensated")	8
S5	TI "Cardiac Failure" OR AB "Cardiac Failure"	1,227
S4	(MH "Heart Failure+")	36,787
S3	S1 OR S2	6,601
S2	TI Bundles OR AB Bundles	6,463
S1	TI Bundle OR AB Bundle	6,572

Cochrane Library (Wiley) 24-09-2019

ID	Search	Hits
#1	MeSH descriptor: [Patient Care Bundles] explode all trees	21
#2	Bundle:ti,ab,kw	1731
#3	Bundles:ti,ab,kw	354
#4	#1 OR #2 OR #3	1968
#5	MeSH descriptor: [Heart Failure] explode all trees	8355
#6	(Cardiac NEAR/1 Failure):ti,ab,kw	993
#7	(Heart NEAR/1 Decompesat*):ti,ab,kw	1
#8	(Cardiac NEAR/1 Decompesat*):ti,ab,kw	0
#9	(Myocardial NEAR/1 Failure):ti,ab,kw	118
#10	(Myocardial NEAR/1 Decompensat*):ti,ab,kw	1
#11	(Heart NEAR/1 Failure):ti,ab,kw	27302
#12	#5 OR #6 OR #7 OR #8 OR #9 OR #10 OR #11	27757
#13	#4 AND #12	338
#14	#13 NOT Branch:ti,ab,kw	32

Global Health (OVID) 26-09-2019

☐	# ▲	Searches	Results
☐	1	Bundle.ti,ab.	1822
☐	2	Bundles.ti,ab.	1467
☐	3	1 or 2	3054
☐	4	Cardiac Failure.ti,ab.	1058

☐	5	Heart Decompensat*.ti,ab.	1
☐	6	Cardiac Decompensat*.ti,ab.	38
☐	7	Myocardial Failure.ti,ab.	61
☐	8	Myocardial Decompensat*.ti,ab.	0
☐	9	Heart Failure.ti,ab.	9089
☐	10	or/4-9	10064
☐	11	3 and 10	66
☐	12	11 not Branch.ti,ab.	17

Supplemental Annex 2. Flowcharts

Flow chart for literature review of HF interventions

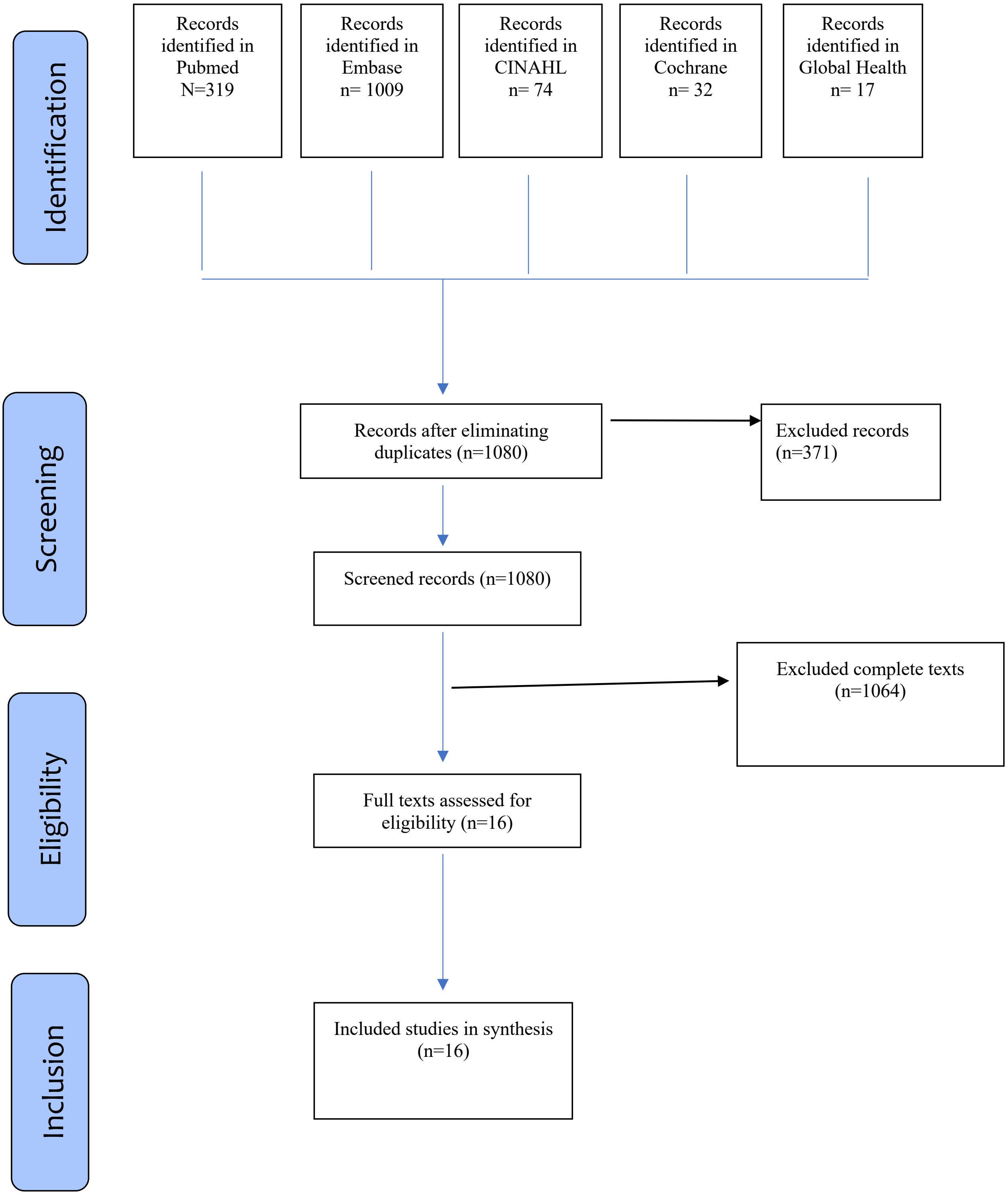

Flow chart for literature review of Readmission

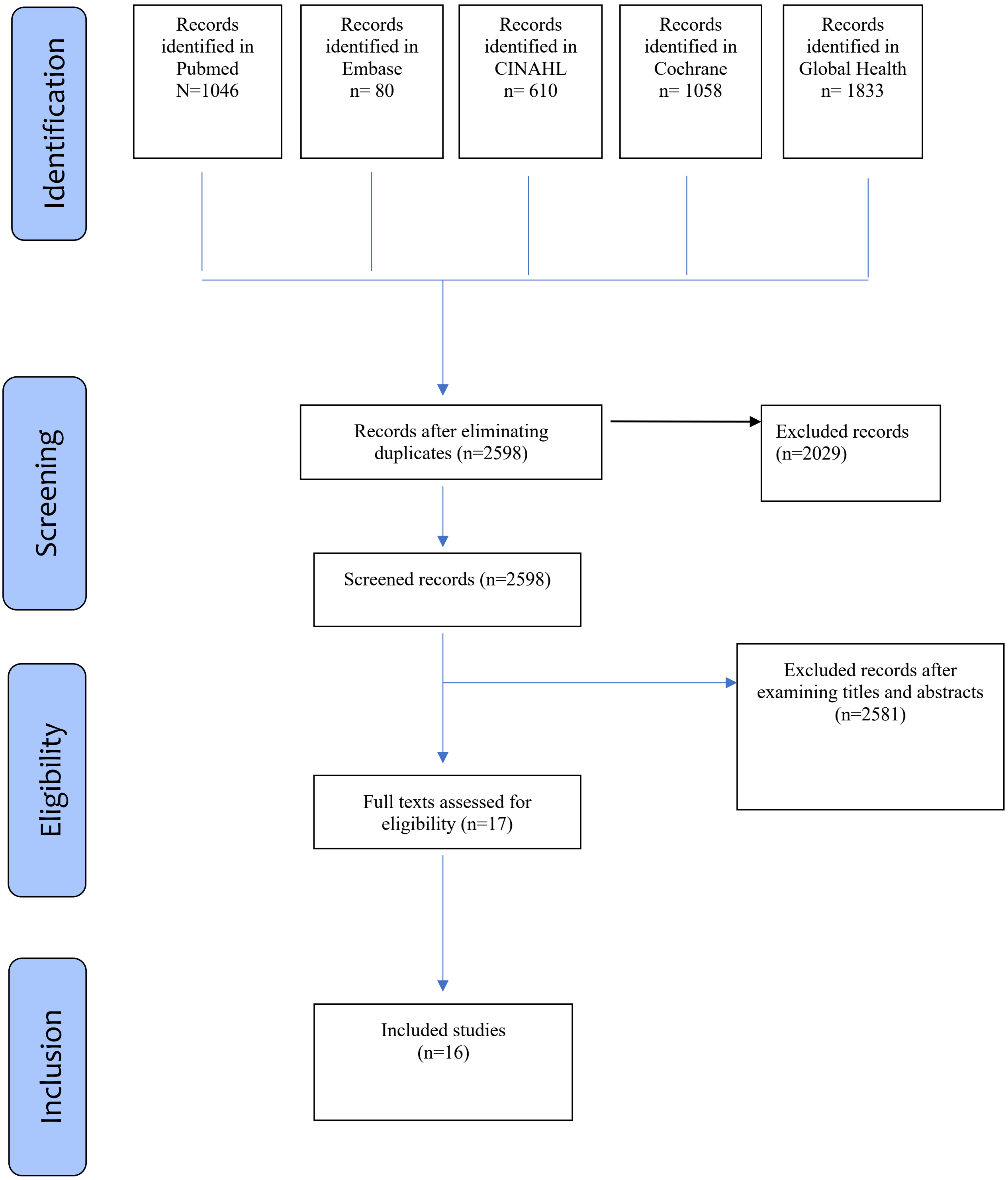

Supplement: Supplementary data [file bmjopen-2020-040028supp001.pdf]
