# Supplementary material for: Care bundle to reduce readmission in patients with heart failure: a modified Delphi consensus panel in Argentina
Source: BMJ Open. 2020 Dec 29;10(12):e040028. doi: 10.1136/bmjopen-2020-040028 (PMC7778781; doi:10.1136/bmjopen-2020-040028)
Supplement: Author's manuscript [file bmjopen-2020-040028.draft_revisions.pdf]

BMJ Open is committed to open peer review. As part of this commitment we make the peer review history of every article we publish publicly available.

When an article is published we post the peer reviewers' comments and the authors' responses online. We also post the versions of the paper that were used during peer review. These are the versions that the peer review comments apply to.

The versions of the paper that follow are the versions that were submitted during the peer review process. They are not the versions of record or the final published versions. They should not be cited or distributed as the published version of this manuscript.

BMJ Open is an open access journal and the full, final, typeset and author-corrected version of record of the manuscript is available on our site with no access controls, subscription charges or pay-per-view fees (http://bmjopen.bmj.com).

If you have any questions on BMJ Open's open peer review process please email info.bmjopen@bmj.com

BMJ Open

Care Bundle to Reduce Readmission in Patients with Heart Failure: A Modified Delphi Consensus Panel in Argentina

Journal:	*BMJ Open*
Manuscript ID	bmjopen-2020-040028
Article Type:	Original research
Date Submitted by the Author:	05-May-2020
Complete List of Authors:	Roberti, Javier; Universidad Nacional de Entre Rios; Institute for Clinical Effectiveness and Health Policy<br>Vita, Tomas; Institute for Clinical Effectiveness and Health Policy<br>Piastrella, Jimena; Novartis Argentina SA<br>Porley, Carlos; Novartis Argentina SA<br>Pereyra, Lisandro; Novartis Argentina SA<br>Diez, Mirta; Instituto Cardiovascular de Buenos Aires<br>Renedo, Florencia; Fundacion Favaloro Hospital Universitario<br>Fairman, Enrique; Clinica Bazterrica<br>Fernandez, Alberto; Sanatorio Modelo Quilmes<br>Thierer, Jorge; CEMIC<br>Garcia Elorrio, Ezequiel; Institute for Clinical Effectiveness and Health Policy
Keywords:	Adult cardiology < CARDIOLOGY, Heart failure < CARDIOLOGY, Quality in health care < HEALTH SERVICES ADMINISTRATION & MANAGEMENT, Protocols & guidelines < HEALTH SERVICES ADMINISTRATION & MANAGEMENT

SCHOLARONE™
Manuscripts

I, the Submitting Author has the right to grant and does grant on behalf of all authors of the Work (as defined in the below author licence), an exclusive licence and/or a non-exclusive licence for contributions from authors who are: i) UK Crown employees; ii) where BMJ has agreed a CC-BY licence shall apply, and/or iii) in accordance with the terms applicable for US Federal Government officers or employees acting as part of their official duties; on a worldwide, perpetual, irrevocable, royalty-free basis to BMJ Publishing Group Ltd ("BMJ") its licensees and where the relevant Journal is co-owned by BMJ to the co-owners of the Journal, to publish the Work in this journal and any other BMJ products and to exploit all rights, as set out in our licence.

The Submitting Author accepts and understands that any supply made under these terms is made by BMJ to the Submitting Author unless you are acting as an employee on behalf of your employer or a postgraduate student of an affiliated institution which is paying any applicable article publishing charge ("APC") for Open Access articles. Where the Submitting Author wishes to make the Work available on an Open Access basis (and intends to pay the relevant APC), the terms of reuse of such Open Access shall be governed by a Creative Commons licence – details of these licences and which Creative Commons licence will apply to this Work are set out in our licence referred to above.

Other than as permitted in any relevant BMJ Author's Self Archiving Policies, I confirm this Work has not been accepted for publication elsewhere, is not being considered for publication elsewhere and does not duplicate material already published. I confirm all authors consent to publication of this Work and authorise the granting of this licence.

For peer review only

Care Bundle to Reduce Readmission in Patients with Heart Failure: A Modified Delphi Consensus Panel in Argentina

Javier Roberti[1*], Tomás Vita[1], Jimena Piastrella[2], Carlos Porley[2], Lisandro Pereyra[2] Mirta Diez[3], Florencia Renedo[4], Enrique Fairman[5], Alberto Fernández[6], Jorge Thierer[7], Ezequiel García Elorrio[1]

1. Institute for Clinical Effectiveness and Health Policy (IECS), Buenos Aires, Argentina
2. Novartis Argentina, Buenos Aires, Argentina
3. Instituto Cardiológico Buenos Aires – ICBA, Buenos Aires, Argentina
4. Fundación Favaloro, Buenos Aires, Argentina
5. Clínica Bazterrica, Buenos Aires, Argentina
6. Clínica Modelo Quilmes, Quilmes, Argentina
7. CEMIC, Buenos Aires, Argentina

***Corresponding Author**

Javier Roberti, Email: jroberti@iecs.org.ar

For peer review only

Abstract

Background. Despite several medical advances to treat heart failure (HF), mortality and hospital readmission have not changed significantly; a large proportion of patients with HF are not receiving adequate treatment. A care bundle is a set of evidence-based interventions applied together in every eligible patient to improve reliability of care and clinical outcomes.

Objective. The aim of this study was to develop consensus among Argentine cardiologists on a care bundle to reduce readmissions in patients with HF.

Methods. This study used a mixed method design and was conducted between August 2019 and January 2020. The approach used to develop a care bundle involved three phases: a) a literature review to define the list of interventions that would be evaluated; b) a modified Delphi panel to select interventions for the bundle; finally, c) development of the HF care bundle. The process included three rounds of scoring.

Results. Twenty-four experts participated in the two online rounds and 18 (75%) of them participated in the third-round meeting. Twenty-six interventions were evaluated, and each obtained a global score. The proposed bundle was based on four categories that comprised 12 specific interventions: medication, continuum of care, lifestyle habits, predischarge exams.

Conclusion. Following a systematic mixed method approach, we have developed a care bundle

For peer review only

of interventions that could decrease readmission of patients with HF. The application of this bundle could contribute to scale evidence-based interventions.

Article summary

Strengths and limitations of this study

- Potential interventions were chosen through a systematic review
- Cardiologist experts participated in a transparent consensus process
- This study could contribute to scale evidence-based interventions
- A care bundle of critical interventions was developed
- As in most consensus processes, participants could have misinterpreted statements

Funding statement: This work was supported by Novartis Argentina SA.

.

Introduction

Despite several medical advances to treat heart failure (HF), mortality and hospital readmission have not changed significantly.[1] Treatment adherence, diet, exercise, regular monitoring, and paperwork demanded by the health system place a significant burden on patients and their carers.[2,3] Moreover, a high percentage of patients with HF

For peer review only

are not receiving adequate treatment despite the increased use of evidence-based therapy and enhanced adherence to performance measures.[4-6] Several initiatives have confirmed to diminish HF readmission; however, they are challenging to replicate. In this context, the use of a care bundle with additional strategies such as quality improvement collaboratives (QICs) to scale-up its use could contribute to the optimization of the treatment of patients with HF.[7,8]

A care bundle is defined as a set of evidence-based interventions, called elements, that should be applied together in every eligible patient to enhance the reliability of care and to improve clinical outcomes.[9,10] The completion of the interventions of a bundle should be measured as all-or-nothing; when all components performed collectively and reliably, they have shown to improve patient outcomes.[11] A care bundle approach for HF should focus on providing evidence-based clinical practice, engaging patients and careers as active partners, and creating processes to ensure a quality handoff from hospital care.

Hospitalizations in HF are the main trigger for treatment interventions; however, financers' lack of awareness of the clinical burden and the low urgency to intervene in these patients compared to other cardiovascular diseases represent significant challenges for implementation.[12,13] In fact, HF guidelines recommend initiating and uptitrating disease-modifying therapies during hospitalization.[14] As the preliminary phase of a future QIC, the aim of this study was to develop consensus among Argentine cardiologists on a care bundle to reduce readmissions in patients with HF.

For peer review only - http://bmjopen.bmj.com/site/about/guidelines.xhtml

Methods

This study used a mixed method design and was conducted between August 2019 and January 2020. The approach used to develop a care bundle involved three phases: a) a literature review to define the list of interventions that would be evaluated; b) a modified Delphi panel to select interventions for the bundle; finally, c) development of the HF care bundle. The process had seven steps, including three rounds of scoring. See Fig 1 for an illustration of the study design.

Figure 1. Flowchart of Delphi process

Phase 1.

Step 1. Explorative review of the literature

In preparation of the Delphi questionnaire, a review of the literature was performed. We searched in PubMed, LILACS, EMBASE, The Cochrane Library and Google Scholar for relevant literature. We included articles describing interventions for HF, with special attention to those specified in the Guidelines of the American College of Cardiology (ACC), the American Heart Association (AHA), the European Society of Cardiology (ESC) and the Argentine Cardiology Society.[7,8,15] We used a pragmatic explorative approach. Supplemental annex 1 shows search strategies used; supplemental annex 2 shows the PRISMA flowchart of selection process. Based on the literature, we proposed a list of 26 interventions to be assessed by experts. Table 1 shows the 26 interventions selected for evaluation.

Table 1. List of interventions proposed

For peer review only

Potential interventions
1. Receive ß-blockers at least at initial dose, plan increase to its maximum tolerable dose during outpatient care.
2. Receive ACE-inhibitor or Angiotensin II antagonists at least at initial dose, plan increase to its maximum tolerable dose during outpatient care.
3. Receive mineralocorticoid antagonists at initial dose, plan increase to maximum tolerable dose if ejection fraction is $\leqq$ 35% during outpatient care.
4. Receive ivabradine at least at initial dose and plan increase to maximum tolerable dose during outpatient care, if heart rate >70 bpm despite ß-blockers at maximum tolerable dose and being in sinus rhythm.
5. Receive ARNI at least at initial dose, plan increase to maximum tolerable dose during outpatient care.
6. Diuretic dose should be adjusted based on results of imaging, laboratory, and functional class examinations.
7. Implant a resynchronizer in case of complete blockage of the left branch with a QRS >0.15 sec, in sinus rhythm, receiving optimal medical treatment and persisting in CF III-IV.
8. Receive antipneucoccal and influenza vaccines.
9. Measure ferritin and correct it if low (<100 ng/mL or 100 to 300 ng/mL if transferrin saturation is <20%).
10. Measure urea, creatinine, calculate GFR and study ionogram (sodium, potassium and chlorine) to establish prognosis and therapy feasibility.
11. Perform cardiac ultrasound and communicate results to treating physician to establish therapeutic action, adjust medication and improve the risk stratification.
12. Perform pulmonary ultrasound and communicate result to treating physician to establish therapeutic action, adjust medication and improve the patient's risk stratification.
13. A pulmonary artery pressure monitoring device should be fitted to improve monitoring and adjust medication by the attending physician.
14. BNP or NT-proBNP should be measured and reported to treating physician to establish therapeutic action, adjust medication and improve the patient's risk stratification.
15. Monitor blood pressure and try to reach values between 130/80 - 110/70, prioritizing use of ß-blockers and ACEi or ARNI.

For peer review only

16. Instruct patient to weigh daily and consult or increase the diuretic dose if increase is ≧ 2kg in 3 days.

17. Indicate patient to ingest a maximum of 2grs of sodium per day and inform on which foods are rich in sodium.

18. If the patient is a smoker, a recommendation will be made for smoking cessation and an action plan to achieve it.

19. Advise patient to lose weight if obese or overweight and not in advanced stages of CHF.

20. Advise patient to perform aerobic exercise at least 4 times/week for 40 min up to 80% of predicted HR for their age.

21. Within 1 week of discharge, specialist nurse should call patient to follow-up on adherence to dietary hygiene measures, medication and its adverse effects, weight gain and monitoring by the cardiologist.

22. Schedule an appointment for a consultation with the specialist within one week of discharge.

23. Patient should be evaluated by a psychologist/psychiatrist to screen for depression and initiate treatment accordingly.

24. Reassure patient that there are no restrictions on sexual activity. If phosphodiesterase inhibitors are used, the patient should be informed about contraindications and precautions.

25. The cause of decompensation of IC should have been identified.

26. if the patient has obstructive sleep apnoea, treatment with continuous positive airway pressure should be started.

For peer review only

Step 2. Developing the list of interventions

The target patient for the interventions was an individual with signs and symptoms of HF and an ejection fraction lower than 40% that went through a hospitalization and will be discharged during the next 48 hours. Based on the literature, we proposed a list of 26 interventions to be assessed by experts. To guide the selection of interventions during

For peer review only - http://bmjopen.bmj.com/site/about/guidelines.xhtml

the consensus process, we used the criteria proposed by GRADE from *Evidence to Decision* framework validated and adopted by WHO (project EtD / DECIDE) and others from the care package literature to help panels of experts use evidence in a structured and transparent way to inform decisions in the context of healthcare recommendations.[10] The potential interventions were presented to panelists as short statements with references to supporting literature. For each statement, panelists were asked to rate interventions by 13 criteria or parameters using a nine-point Likert scale ranging from 1 (extremely inappropriate / insignificant) to 9 (extremely appropriate/ significant) [16]. The 13 criteria were 1) priority, 2) unintended effects, 3) intended effects, 4) balance between unintended and intended effects, 5) quality of evidence on effects, 6) values and preferences, 7) resource required, 8) quality of evidence on resources, 9) cost-effectiveness, 10) equity, 11) acceptability, 12) feasibility, and 13) measurability. The EtD framework ensures that panels consider important criteria, structure discussion and identify reasons for disagreements, based on the balance of health benefits and harms, human rights and sociocultural acceptability, health equity, equality and non-discrimination, societal implications, financial and economic considerations, feasibility and health system considerations and the meta-criterion quality of evidence.[17,18]

Phase 2. Consensus on a care bundle.

Step 1. Selection of experts' panel

We used a modified RAND-UCLA Delphi method to select interventions for HF care bundle to be applied during the discharge process care to prevent readmissions, where an expert panel members use their professional judgment alongside the best available

For peer review only

evidence to identify areas where consensus can be reached for the topic under consideration.[16]

In the second stage, 26 experts in chronic HF were selected from different clinical contexts in Argentina, mainly from the high-density area of Buenos Aires. All participants needed to have ample treatment experience in patients with HF and experience in scientific research on HF. Twenty-six potential participants were sent a recruitment letter and information sheet via e-mail, follow-up telephone calls were made to confirm reception of material. Of the total, 24 (92%) professionals accepted to participate. Panelists were given a small honorarium for their time and effort.

Step 2. Interventions rating

Permanent disagreement was defined as at least six panelists rating the indication in the 1-3 region, and at least six panelists rating it in the 7-9 region after three rounds of scoring. Each intervention obtained 13 scores, one for each parameter or criterion.[16] Then, this score was multiplied by the weighting factor obtained in the rating of the parameters and mean was calculated for each intervention. Interventions were then sorted according to this global score.

Step 3. First round online survey

For the first round, the experts received a link for the online questionnaire (surveymonkey.com, San Mateo, California, USA). Twenty-six interventions in 13 criteria were evaluated for disagreement following the classic RAND definition of at least six panelists rating the parameter in the 1-3 region and at least six panelist rating it in the 7-

For peer review only

9 region.[16] Security sockets layer (SSL) encryption is used to protect data while being transmitted by ensuring secure connections between participants and the server. An email was issued to each participant requesting them to open the survey monkey tool, answer the questions and press the submit button. All participants had the contact details for the research team, who they could contact if they had any doubts regarding the materials provided. Participants had 10 days to complete the survey, with a reminder email sent to non-responders.

Step 4. Second online survey round

In round two, all interventions and their criteria that did not reach an agreement in the first round were included for a new vote. Participants were shown the whole group's ratings as frequency data on a rating scale for each item, alongside their own ratings for each statement from round one. They could revise their own original rating considering the group ratings if they so wished. Participants had 10 days to complete round two with a reminder sent to non-responders.

Step 5. Consensus meeting

All participants of the online survey were invited via email and telephone to a three-hour in person consensus meeting. The main objectives of the meeting were to discuss the indications that have been rated with disagreement and inconsistencies in appropriateness ratings. Panelists were encouraged to refer to the literature. Of the total of participants who responded the online rounds, 18 (75%) took part in the meeting.

For peer review only

During the session, results of the second online survey were presented and discussed, followed by an anonymous onsite rating of those items that had showed disagreement on previous round. In addition, the 13 quality criteria were anonymously rated using a 1-9 Likert scale to obtain a factor for final evaluation of each intervention. The panel mean score was calculated for each statement and classified into three categories: 1–3.5 (potentially inappropriate); 3.6– 6.4 (uncertain), and 6.5–9 (potentially appropriate). The moderator was a physician, not a specialist in the area being rated to avoid bringing their bias to the discussion, he had led the literature review and was familiar with the material.

Step 6. Development of HF care bundle with expert committee

We invited five of the participants of previous rounds with extensive expertise to join an expert committee to discuss the results of the survey and rate the bundle. The results of the whole process were presented in this meeting. The expert committee selected those interventions with the highest scores for the final evaluation and no disagreements. Based on these results, a final bundle of interventions was developed.

Ethical aspects and role of funding source

Written informed consent was obtained from all participants included in the study, and all procedures were conducted according to the Declaration of Helsinki. This study was evaluated and approved by an independent ethics committee.

For peer review only - http://bmjopen.bmj.com/site/about/guidelines.xhtml

Patient and Public Involvement

The research question addresses a most significant issue for patients and was informed by a review of the literature on patients' journey when admitted for heart failure care. Only physicians with a vast clinical experience participated in the consensus process; these participants brought patients' concerns and perspectives into the process. The results of this study are disseminated via open access publications. This study is complemented by a qualitative research on patients' perspectives, reported separately.

Results

Twenty-four experts participated in the two online rounds and 18 (75%) of them participated in the third-round meeting. Eight (33.3%) were female; 16 (66.7%) participants worked in the private sector, 2 (8.3%) in the public sector and 6 (25%) in both sectors. Sixteen (66.7%) participants worked in the metropolitan area of Buenos Aires, while the rest, 8 (33.3%), worked in other provinces of Argentina. The median of professional experience was 26.5 years (range, 9 – 40 years).

Twenty-six interventions were evaluated, and each intervention obtained a global score. After the three rounds, consensus was reached for all interventions except for one intervention under one criterion (unintended effects of telephone tracking of patients). The mean global scores of 13 interventions were above the cutoff score of 6.5 and seven of these scored a median of 7.1 (Supplemental table 1). Of the 13 final interventions, 5 (38%) were related to medications, 2 (15%) were diagnostic tests, and 2 (15%) were habit

For peer review only - http://bmjopen.bmj.com/site/about/guidelines.xhtml

recommendations (hyposodic diet and daily weight monitoring). The two most valuable criteria ranked by experts were priority and desirable effects of the intervention. Administration of ACE-inhibitors, renal function and ionogram evaluation and blood pressure control were considered the most important interventions. The use of beta-blockers was the fifth intervention in the ranking of potential interventions while the use of anti-mineralocorticoids, furosemide and ARNI were ninth, tenth and eleventh, respectively. The use of ARNI ranked better on undesirable effects than the administration of furosemide and anti-mineralocorticoids. The administration of sacubitril/valsartan and the use of ACE-inhibitors obtained the same scores in six criteria; mainly related to scientific evidence evaluation; of note under the criteria that evaluated equity, ACE-inhibitors ranked higher. Smoking cessation and blood pressure control were ranked better than the use of medication because, unlike medication, the first two had no potential undesirable effects according to participants.

Daily weight monitoring and a follow-up appointment within a week of discharge were fourth and seventh, respectively. While echocardiography during hospitalization and determining the cause of decompensation were the interventions positioned last in the list of elements that could be included into the bundle, above the established cut-off point. Measurement of biomarker pro-BNP and BNP ranked low and did not make the cutoff. Physical exercise and cardiac resynchronization therapy did not rank high for the overall goal of reducing hospital readmission.

For peer review only

Proposed Bundle

The proposed bundle was based on four categories of interventions: medication, continuum of care, lifestyle habits, predischarge tests. See Table 2. Beta-blockers, angiotensin receptor/neprilysin inhibitor (ARNI) or angiotensin-converting enzyme inhibitors (ACE-inhibitors), furosemide and anti-mineralocorticoids were included as interventions related to pharmacologic treatment. ARNI or ACE-inhibitors can be used indistinctly. The election would depend on clinician judge and patient choice. Follow-up appointment with a specialist within a week of discharge and daily weight monitoring were also included as interventions that reduce 30-day hospital readmission under "continuum of care" category, while smoking cessation counseling and low sodium diet were interventions classified as lifestyle habits. The interventions included under pre discharge tests were blood test – renal function and ionogram evaluation, blood pressure control, echocardiogram during hospitalization and determination of the decompensating cause.

Table 2. Proposed Bundle

Interventions	
Pre discharge medications	Beta Blockers – reach optimal dose
	ACE-inhibitors or ARNI– reach optimal dose
	Anti-mineralocorticoids – reach optimal dose
	Furosemide – reach optimal dose
Continuum of care after discharge	Follow up appointment with the specialist seven days after discharge
	Daily weight monitoring under same conditions

For peer review only - http://bmjopen.bmj.com/site/about/guidelines.xhtml

Lifestyle habits	Low sodium diet – intake of <2grs of sodium per day
	Tobacco cessation counseling
Predischarge tests	Renal function and ionogram evaluation
	Blood pressure control (pre-discharge)
	Echocardiogram during hospitalization
	Determination of the decompensating cause

For peer review only

Discussion

Following a validated and systematic consensus method, we created a care bundle of interventions that could contribute to the management of HF and prevent readmission within the 30 days following discharge. The reduction of readmissions for patients with HF represents a challenge due to various factors such as a lack of targeted therapy for patients with preserved ejection fraction, the natural history of HF, the need for daily management to avoid decompensations, and problems related to access to care, health literacy, and other socioeconomic factors.[19] HF is a complex syndrome and the result of congestion and/or poor perfusion leads to negative effects on multiple organ systems. In fact, patients with HF have multiple comorbidities, a long list of medications, and symptoms that may be attributed to a number of etiologies.[19]

However, several studies have evaluated single interventions to improve outcomes in patients with HF; ESC, ACC and HF Argentinean guidelines have established recommendations but interventions considered critical have not been specified.[7,8,15] For

For peer review only - http://bmjopen.bmj.com/site/about/guidelines.xhtml

participants, the intervention's priority and its desirable effects were the two most important criteria to evaluate each intervention. Probably, these two criteria are what cardiologists in the local context refer to when assessing any treatment in time-constrained settings. We have defined priorities in a time constrained setting as it is usually the case with clinical work. As all the potential interventions had been recommended on HF guidelines, no significant differences in global scores were observed among those above the cut-off point. Indeed, when the results were shown to the experts, all of them agreed on the significance of the interventions that finally included in the bundle.

Almost half of the interventions were related to medication, which, with robust evidence supporting its efficacy, is widely accepted as the backbone of HF treatment. It has been shown that interventions to improve adherence to medications for HF significantly reduce the risk for both hospitalization and death.[4] Interestingly, the use of beta-blockers was only fifth on the interventions ranking although beta-blockers have been confirmed to reduce mortality, readmission and improve symptomatology.[20-23] It is possible that the use of beta-blockers was not chosen as the most important interventions in our study as many patients have HF in very advanced stages and require inotropes during hospitalization while beta-blockers are administered during outpatient care. Clinical trials angiotensin receptor/neprilysin inhibitor (ARNI) studies showed a significant reduction in mortality and readmission when compared to angiotensin-converting enzyme inhibitors (ACE-inhibitors).[24,25] The administration of ACE-inhibitors was pondered as the most important intervention and, at a granular level, the difference between ARNI and ACE-inhibitors was based mainly on costs and access. The high cost of the drug in an economically

constrained setting explains the gap between evidence and real-world practice. Due to new evidence in recent years, the final recommendations in the bundle allow the physician to decide between ARNI or ACE-inhibitors.[24,25]

Interestingly, many of the interventions were not strictly clinical issue such as daily weight monitoring or a follow-up appointment; reflecting the importance of an integrated care and the complex setting in which professionals and the health system have to interact to achieve the best quality outcomes. Importantly, laboratory examinations such as tests of renal function and the ionogram, obtained a high score and valued as the second most important intervention. Of the five medications included in the bundle, four could negatively affect renal function. Following the *primum non nocere* principle is that physicians first want to assure that medication is not harming. On the other hand, in the Argentinian health system, BNP test is expensive and is not covered by most health insurances which could explain the fact that this test was not included in the final list of elements.

In pioneering care bundles, two components were essential to their success; participating clinicians agreed that there was sufficient medical evidence supporting each individual element in the bundle to recommend that it be applied to most, if not all, patients; at a minimum, each element should be considered for every patient.[26,29] Second, the list of elements in the bundle should not be extensive. The goal of the bundle approach is to develop a short list of interventions that are already recommended and accepted in guidelines; this allows improvement rather than a debate on validity of the elements. It is essential that elements have the consensus of local clinicians.[30] On the other hand, checklists and protocols serve to augment memory and limit the chance of human error,

For peer review only - http://bmjopen.bmj.com/site/about/guidelines.xhtml

improving communication, standardizing responses, reducing unnecessary clinical variation.[31]

Our study has limitations that should be acknowledged. Participants could have misinterpreted the original statements describing the potential interventions; the same applies to criteria definitions. We tried to control this factor by providing participants with information and discussing definitions during the meeting. Also, we disclose the funding by a pharmaceutical company and the participation of professionals affiliated to this company, which could be interpreted as a limitation. We have made every effort to maintain the transparency of the process with the participation of professionals in every stage.

In conclusion, following a mixed method approach we developed a care bundle of interventions that could decrease readmission of patients with HF. The application of this bundle could be used to scale evidence-based interventions under the framework of Quality Improvement Learning Collaborative.

Acknowledgements

We would like to thank the participation of the following health professionals: José Luis Barisani (Clínica Adventista), Roberto Colque (Sanatorio Allende), Marcelo Crespo (Sanatorio Los Arcos), Clara Huerta (Hospital Córdoba), Juan Pablo Escalante (Instituto Cardiovascular Rosario), David Flores (Hospital Nacional de Clínicas), Jimena Gambarte

(Hospital Alemán), Daniela García Brasca (Hospital Italiano de Córdoba), Alfredo Larraburu (Sanatorio Los Arcos), Adrián Lescano (Centro Gallego), Javier Marino (ICBA), Lidia Lobo Márquez (Instituto de Cardiología), Cristian Mastantuono (Hospital Pirovano), Felipe Martínez (Universidad Nacional de Córdoba), Stella Pereiro (Hospital Churruca), Eduardo Perna (Instituto de Cardiología de Corrientes Juana Francisca Cabral), José Santucci (Hospital Austral), Julieta Soricetti (Hospital Durand), Ezequiel Zaidel (Sanatorio Güemes).

A. **Contributorship statement:** Javier Roberti: Methodology, investigation, analysis, writing. Tomas Vita: investigation, analysis, writing. Jimena Piastrella, Carlos Porley, and Lisandro Pereyra: investigation, interpretation, review; Mirta Diez, Florencia Renedo, Enrique Fairman, Alberto Fernández, Jorge Thierer: investigation, interpretation, review; Ezequiel García Elorrio: concept, methodology, analysis, interpretation, review.

B. **Competing interests:** Jimena Piastrella, Carlos Porley, and Lisandro Pereyra are employees of Novartis Argentina SA, funder of this study. Javier Roberti, Tomas Vita, Florencia Renedo, Enrique Fairman, Alberto Fernandez, Jorge Thierer and Ezequiel Garcia Elorrio have no competing interest to declare.

C. **Funding**: this study was funded by Novartis Argentina SA.

D. **Data sharing statement**: Supplementary data such as search strategies, flowcharts and table of scores are available in supplementary materials and upon request.

For peer review only - http://bmjopen.bmj.com/site/about/guidelines.xhtml

- **Authors' Contributions:** state how each author was involved in writing the article.
- **Funding statement:** preferably worded as follows. Either: 'This work was supported by [name of funder] grant number [xxx]' or 'This research received no specific grant from any funding agency in the public, commercial or not-for-profit sectors'.
- **data sharing statement**, such as: "Technical appendix, statistical code, and dataset available from the Dryad repository, DOI: [include DOI for dataset here].

References

1. Bytyci I, Bajraktari G. Mortality in heart failure patients. Anatol J Cardiol. 2015;15(1):63-8. Epub 2015/01/01. doi: 10.5152/akd.2014.5731. PubMed PMID: 25550250; PubMed Central PMCID: PMCPMC5336901.

2. Nordfonn OK, Morken IM, Bru LE, Husebo AML. Patients' experience with heart failure treatment and self-care-A qualitative study exploring the burden of treatment. J Clin Nurs. 2019;28(9-10):1782-93. Epub 2019/01/23. doi: 10.1111/jocn.14799. PubMed PMID: 30667120.

3. Evangelista LS, Shinnick MA. What do we know about adherence and self-care? J Cardiovasc Nurs. 2008;23(3):250-7. Epub 2008/04/26. doi: 10.1097/01.JCN.0000317428.98844.4d. PubMed PMID: 18437067; PubMed Central PMCID: PMCPMC2880251.

4. Ruppar TM, Cooper PS, Mehr DR, Delgado JM, Dunbar-Jacob JM. Medication Adherence Interventions Improve Heart Failure Mortality and Readmission Rates: Systematic Review and Meta-Analysis of Controlled Trials. J Am Heart Assoc. 2016;5(6). Epub 2016/06/19. doi: 10.1161/JAHA.115.002606. PubMed PMID: 27317347; PubMed Central PMCID: PMCPMC4937243.

For peer review only

5. Fonarow GC, Abraham WT, Albert NM, Gattis Stough W, Gheorghiade M, Greenberg BH, et al. Influence of a performance-improvement initiative on quality of care for patients hospitalized with heart failure: results of the Organized Program to Initiate Lifesaving Treatment in Hospitalized Patients With Heart Failure (OPTIMIZE-HF). Arch Intern Med. 2007;167(14):1493-502. doi: 10.1001/archinte.167.14.1493. PubMed PMID: 17646603.

6. Panella M, Marchisio S, Demarchi ML, Manzoli L, Di Stanislao F. Reduced in-hospital mortality for heart failure with clinical pathways: the results of a cluster randomised controlled trial. Qual Saf Health Care. 2009;18(5):369-73. doi: 10.1136/qshc.2008.026559. PubMed PMID: 19812099.

7. Ponikowski P, Voors AA, Anker SD, Bueno H, Cleland JG, Coats AJ, et al. 2016 ESC Guidelines for the diagnosis and treatment of acute and chronic heart failure: The Task Force for the diagnosis and treatment of acute and chronic heart failure of the European Society of Cardiology (ESC)Developed with the special contribution of the Heart Failure Association (HFA) of the ESC. Eur Heart J. 2016;37(27):2129-200. doi: 10.1093/eurheartj/ehw128. PubMed PMID: 27206819.

8. Yancy CW, Jessup M, Bozkurt B, Butler J, Casey DE, Jr., Colvin MM, et al. 2017 ACC/AHA/HFSA Focused Update of the 2013 ACCF/AHA Guideline for the Management of Heart Failure: A Report of the American College of Cardiology/American Heart Association Task Force on Clinical Practice Guidelines and the Heart Failure Society of America. J Am Coll Cardiol. 2017;70(6):776-803. doi: 10.1016/j.jacc.2017.04.025. PubMed PMID: 28461007.

9. Lavallee JF, Gray TA, Dumville J, Russell W, Cullum N. The effects of care bundles on patient outcomes: a systematic review and meta-analysis. Implement Sci. 2017;12(1):142. doi:

For peer review only

10.1186/s13012-017-0670-0. PubMed PMID: 29187217; PubMed Central PMCID: PMCPMC5707820.

10. Resar R, Griffin F, Haraden C, Nolan T. Using Care Bundles to Improve Health Care Quality. Cambridge, Massachusetts: Institute for Healthcare Improvement;, 2012.

11. Quality AfHRa. Eliminating CLABSI, A National Patient Safety Imperative: Final Report. Rockville, MD.: 2013.

12. Arora S, Patel P, Lahewala S, Patel N, Patel NJ, Thakore K, et al. Etiologies, Trends, and Predictors of 30-Day Readmission in Patients With Heart Failure. Am J Cardiol. 2017;119(5):760-9. Epub 2017/01/23. doi: 10.1016/j.amjcard.2016.11.022. PubMed PMID: 28109560.

13. Wan TTH, Terry A, Cobb E, McKee B, Tregerman R, Barbaro SDS. Strategies to Modify the Risk of Heart Failure Readmission: A Systematic Review and Meta-Analysis. Health Serv Res Manag Epidemiol. 2017;4:2333392817701050. Epub 2017/05/04. doi: 10.1177/2333392817701050. PubMed PMID: 28462286; PubMed Central PMCID: PMCPMC5406120.

14. Seferovic PM, Ponikowski P, Anker SD, Bauersachs J, Chioncel O, Cleland JGF, et al. Clinical practice update on heart failure 2019: pharmacotherapy, procedures, devices and patient management. An expert consensus meeting report of the Heart Failure Association of the European Society of Cardiology. Eur J Heart Fail. 2019;21(10):1169-86. Epub 2019/05/28. doi: 10.1002/ejhf.1531. PubMed PMID: 31129923.

For peer review only

15. Cardiología SAd. Consenso de insuficiencia cardíaca crónica. Revista Argentina de Cardiología. 2016;84.

16. Fitch K. The Rand/UCLA appropriateness method user's manual. Santa Monica: Rand; 2001.

17. Moberg J, Oxman AD, Rosenbaum S, Schunemann HJ, Guyatt G, Flottorp S, et al. The GRADE Evidence to Decision (EtD) framework for health system and public health decisions. Health Res Policy Syst. 2018;16(1):45. Epub 2018/05/31. doi: 10.1186/s12961-018-0320-2. PubMed PMID: 29843743; PubMed Central PMCID: PMCPMC5975536.

18. Alonso-Coello P, Schunemann HJ, Moberg J, Brignardello-Petersen R, Akl EA, Davoli M, et al. [GRADE Evidence to Decision (EtD) frameworks: a systematic and transparent approach to making well informed healthcare choices. 1: Introduction]. Gac Sanit. 2018;32(2):166 e1- e10. Epub 2017/08/22. doi: 10.1016/j.gaceta.2017.02.010. PubMed PMID: 28822594.

19. Goldgrab D, Balakumaran K, Kim MJ, Tabtabai SR. Updates in heart failure 30-day readmission prevention. Heart Fail Rev. 2019;24(2):177-87. Epub 2018/11/30. doi: 10.1007/s10741-018-9754-4. PubMed PMID: 30488242.

20. Hjalmarson A, Goldstein S, Fagerberg B, Wedel H, Waagstein F, Kjekshus J, et al. Effects of controlled-release metoprolol on total mortality, hospitalizations, and well-being in patients with heart failure: the Metoprolol CR/XL Randomized Intervention Trial in congestive heart failure (MERIT-HF). MERIT-HF Study Group. JAMA. 2000;283(10):1295-302. doi: 10.1001/jama.283.10.1295. PubMed PMID: 10714728.

For peer review only

21. Packer M, Coats AJ, Fowler MB, Katus HA, Krum H, Mohacsi P, et al. Effect of carvedilol on survival in severe chronic heart failure. N Engl J Med. 2001;344(22):1651-8. doi: 10.1056/NEJM200105313442201. PubMed PMID: 11386263.

22. Packer M, Bristow MR, Cohn JN, Colucci WS, Fowler MB, Gilbert EM, et al. The effect of carvedilol on morbidity and mortality in patients with chronic heart failure. U.S. Carvedilol Heart Failure Study Group. N Engl J Med. 1996;334(21):1349-55. doi: 10.1056/NEJM199605233342101. PubMed PMID: 8614419.

23. Effect of metoprolol CR/XL in chronic heart failure: Metoprolol CR/XL Randomised Intervention Trial in Congestive Heart Failure (MERIT-HF). Lancet. 1999;353(9169):2001-7. PubMed PMID: 10376614.

24. McMurray JJ, Packer M, Desai AS, Gong J, Lefkowitz MP, Rizkala AR, et al. Angiotensin-neprilysin inhibition versus enalapril in heart failure. N Engl J Med. 2014;371(11):993-1004. doi: 10.1056/NEJMoa1409077. PubMed PMID: 25176015.

25. Wachter R, Senni M, Belohlavek J, Straburzynska-Migaj E, Witte KK, Kobalava Z, et al. Initiation of sacubitril/valsartan in haemodynamically stabilised heart failure patients in hospital or early after discharge: primary results of the randomised TRANSITION study. Eur J Heart Fail. 2019;21(8):998-1007. doi: 10.1002/ejhf.1498. PubMed PMID: 31134724.

26. Resar R, Pronovost P, Haraden C, Simmonds T, Rainey T, Nolan T. Using a Bundle Approach to Improve Ventilator Care Processes and Reduce Ventilator-Associated Pneumonia. The Joint Commission Journal on Quality and Patient Safety. 2005;31(5):243-8. doi: 10.1016/s1553-7250(05)31031-2.

For peer review only

27. Burger CD, Resar RK. "Ventilator bundle" approach to prevention of ventilator-associated pneumonia. Mayo Clin Proc. 2006;81(6):849-50. Epub 2006/06/15. doi: 10.4065/81.6.849. PubMed PMID: 16770987.

28. Bird D, Zambuto A, O'Donnell C, Silva J, Korn C, Burke R, et al. Adherence to ventilator-associated pneumonia bundle and incidence of ventilator-associated pneumonia in the surgical intensive care unit. Arch Surg. 2010;145(5):465-70. Epub 2010/05/19. doi: 10.1001/archsurg.2010.69. PubMed PMID: 20479345.

29. Furuya EY, Dick A, Perencevich EN, Pogorzelska M, Goldmann D, Stone PW. Central line bundle implementation in US intensive care units and impact on bloodstream infections. PLoS One. 2011;6(1):e15452. Epub 2011/01/27. doi: 10.1371/journal.pone.0015452. PubMed PMID: 21267440; PubMed Central PMCID: PMCPMC3022589.

30. Resar R, Griffin F, Haraden C, Nolan T. Using Care Bundles to Improve Health Care Quality. . Cambridge, Massachusetts: Institute for Healthcare Improvements; 2012.

31. Arora KS, Shields LE, Grobman WA, D'Alton ME, Lappen JR, Mercer BM. Triggers, bundles, protocols, and checklists--what every maternal care provider needs to know. Am J Obstet Gynecol. 2016;214(4):444-51. Epub 2015/10/20. doi: 10.1016/j.ajog.2015.10.011. PubMed PMID: 26478105.

Supporting information

For peer review only

S1 Table S1. Interventions and criteria pondering results

S2. Supplemental Annex 1. Search strategies

S3. Supplemental Annex 2. Flowcharts for literature review

For peer review only

Figure 1. Flowchart of Delphi process

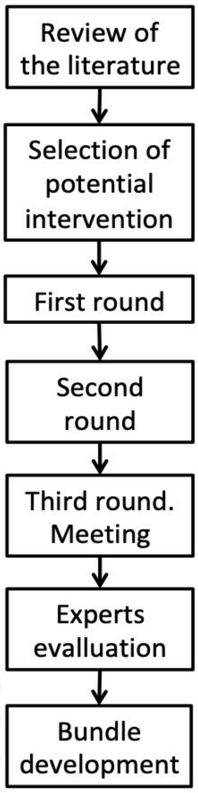

For peer review only

Supplemental Table 1. Interventions and criteria pondering results

	1. Priority	2. Intended effects	3. Unintended effects	4. Balance	5. Quality of evidence on effects	6.Values and preferences	7. Resource required	8. Quality of evidence on resources	9. Cost-effectiveness	10. Equity	11. Acceptability	12. Feasibility	13. Measurability		
Weighting factor	1.00	1.00	0.89	0.89	0.89	0.78	0.78	0.78	0.89	0.89	0.78	0.89	0.78	Score	
Recommendation															
ACE-inhibitors	9.00	9.00	2.67	7.11	8.00	6.22	7.00	6.22	8.00	7.11	7.00	8.00	7.00	**96.00**	7.38
Lab	9.00	9.00	1.78	7.11	7.11	6.22	7.00	5.44	8.00	7.56	7.00	8.00	7.00	**95.67**	7.36
Blood Pressure	8.00	8.00	2.67	7.11	7.11	6.22	7.00	6.22	8.00	7.11	6.61	8.00	6.61	**92.33**	7.10
Dairy weight	8.50	8.50	1.78	8.00	6.22	6.22	6.22	5.44	7.11	7.56	6.22	7.11	6.22	**90.56**	6.97
Beta block	9.00	9.00	2.67	7.11	8.00	6.22	6.22	2.72	7.11	7.11	7.00	7.56	7.00	**90.39**	6.95
Sodium	8.50	8.00	1.78	7.11	6.22	5.44	7.00	5.44	7.56	7.56	6.22	7.56	5.44	**89.28**	6.87
Follow-up	9.00	9.00	1.78	7.56	6.22	6.22	4.67	5.44	7.11	7.11	6.22	7.11	6.22	**89.11**	6.85
Smoking cesation	9.00	9.00	2.22	7.11	7.11	4.67	5.44	5.44	7.11	7.11	6.22	6.22	5.44	**86.67**	6.67
Anti mineralocorticoids	8.00	8.00	4.44	6.22	8.00	5.44	6.22	6.22	7.11	7.11	6.22	7.11	6.22	**86.44**	6.65
Diuretics	9.00	8.00	4.44	6.67	5.33	5.44	6.61	5.44	7.11	6.67	6.22	8.00	7.00	**86.06**	6.62
Sacubitril	8.00	9.00	2.67	7.11	8.00	6.22	4.67	6.22	6.22	5.33	5.44	6.22	7.00	**85.78**	6.60
Echocardiography	8.00	8.00	0.89	7.11	6.22	6.22	3.89	4.67	7.11	6.22	6.22	6.22	6.61	**84.61**	6.51
Descompensation Cause	9.00	8.50	4.44	7.11	6.22	6.22	5.06	5.44	7.11	6.67	6.22	6.22	6.22	**84.56**	6.50
Telephone tracking	8.00	8.00	1.78	7.11	6.22	6.22	4.28	5.44	6.67	6.22	5.44	5.33	6.22	**82.39**	6.34
Flu vaccine	7.00	7.00	2.22	6.67	5.33	5.44	5.44	4.67	7.11	7.11	6.22	7.11	6.22	**82.11**	6.32
Weightloss	7.00	7.00	2.67	6.22	5.33	4.67	5.83	4.67	6.22	5.78	5.44	6.22	5.44	**76.17**	5.86
Exercicse	7.00	7.00	7.11	7.11	6.22	5.44	6.22	3.89	6.22	7.11	5.44	6.22	5.44	**75.22**	5.79
Sexual activity	7.00	7.00	4.00	6.22	4.44	5.44	6.22	4.67	6.22	6.22	4.67	6.22	4.67	**74.00**	5.69
ProBNP	7.00	7.00	1.78	6.22	6.22	5.44	3.89	5.44	4.44	3.56	3.89	4.44	6.22	**71.00**	5.46
Lung echography	6.00	6.00	1.78	6.67	5.33	5.06	3.89	4.67	4.89	4.44	5.06	5.33	6.22	**70.78**	5.44
Psychologist	6.00	7.00	3.56	6.22	5.33	4.67	4.67	4.67	6.22	5.33	4.67	4.44	4.67	**69.33**	5.33
Ferritin	7.00	3.00	2.67	6.22	6.22	4.67	3.89	3.89	5.78	4.44	4.67	5.33	5.83	**67.28**	5.18
Ivabrav	6.00	6.00	2.67	6.22	6.22	4.67	5.44	4.67	5.33	4.44	4.28	6.22	5.83	**67.22**	5.17
Apnea	7.00	7.00	3.56	6.22	5.78	4.67	2.33	4.67	5.33	3.56	4.67	4.00	5.44	**66.11**	5.09
Resynchronizer	6.00	4.00	3.56	6.22	6.67	4.67	1.56	5.44	5.33	2.67	4.67	4.00	6.22	**62.89**	4.84
Wireless HF monitor	3.00	6.00	2.67	4.44	5.33	2.33	0.78	4.28	4.44	1.78	2.72	1.78	5.44	**48.67**	3.74

Supplemental Annex 1. Search strategies

Search	Query	Items found
#17	Search **(#15 AND #16)**	1046
#16	Search **((Systematic Review[sb] OR Systematic Review[tiab] OR Meta-Analysis[pt] OR Meta-Analys*[tiab] OR "Cochrane Database Syst Rev"[ta] OR Metaanalysis[tiab] OR Metanalysis[tiab] OR Sysrev_Methods[sb] OR (MEDLINE[tiab] AND Cochrane[tiab]) OR Guideline[pt] OR Practice Guideline[pt] OR Guideline*[ti] OR Guide Line*[tiab] OR Consensus[tiab] OR Recommendation*[ti] OR Randomized Controlled Trial[pt] OR Random*[ti] OR Controlled Trial*[tiab] OR Control Trial*[tiab] OR Technology Assessment, Biomedical[Mesh] OR Technology Assessment[tiab] OR Technology Appraisal[tiab] OR HTA[tiab] OR Overview[ti] OR (Review[ti] AND Literature[ti])))**	1277580
#15	Search **(#8 AND #14)**	5959
#14	Search **(#9 OR #10 OR #11 OR #12 OR #13)**	37520
#13	Search **Re-Hospitaliz*[tiab]**	1206
#12	Search **Rehospitaliz*[tiab]**	5251
#11	Search **Readmitted[tiab]**	6511
#10	Search **Readmission*[tiab]**	24703
#9	Search **Patient Readmission[Mesh]**	15507
#8	Search **(#1 OR #2 OR #3 OR #4 OR #5 OR #6 OR #7)**	204191
#7	Search **Heart Failure[tiab]**	163364
#6	Search **Myocardial Decompensat*[tiab]**	33
#5	Search **Myocardial Failure[tiab]**	757
#4	Search **Cardiac Decompensat*[tiab]**	1156
#3	Search **Heart Decompensat*[tiab]**	122
#2	Search **Cardiac Failure[tiab]**	11760
#1	Search **Heart Failure[MeSH]**	115775

No.	Query	Results
#19	#17 AND #18	**80**
#18	((((('systematic review':ti,ab OR 'meta analysis (topic)':pt OR 'meta analysis':ti,ab OR cochrane:jt OR metaanalysis:ti,ab OR metanalysis.ti,ab. OR (medline:ab AND cochrane:ab) OR practice) AND guideline:pt OR guideline*:ti OR 'guide lines':ti,ab OR consensus:ti,ab OR recommendation*:ti OR randomized) AND controlled AND trial:pt OR random*:ti OR controlled) AND trial*:ti,ab OR 'control trial':ti,ab OR biomedical) AND technology AND 'assessment'/exp OR 'technology assessment':ti,ab OR 'technology appraisal':ti,ab OR hta:ti,ab OR overview:ti OR (review:ti AND literature:ti)	**194196**

For peer review only

No.	Query	Results
#17	#8 AND #16	**16307**
#16	#9 OR #10 OR #11 OR #12 OR #13 OR #14 OR #15	**75899**
#15	're hospitaliz*':ti,ab	**3444**
#14	're hospitalis*':ti,ab	**486**
#13	rehospitalis*:ti,ab	**1208**
#12	rehospitaliz*:ti,ab	**8201**
#11	readmitted:ti,ab	**13183**
#10	readmission*:ti,ab	**45071**
#9	'hospital readmission'/exp	**56483**
#8	#1 OR #2 OR #3 OR #4 OR #5 OR #6 OR #7	**536876**
#7	'heart failure':ti,ab	**259617**
#6	(myocardial NEAR/1 decompensat*):ti,ab	**57**
#5	'myocardial failure':ti,ab	**934**
#4	(cardiac NEAR/1 decompensat*):ti,ab	**1917**
#3	(heart NEAR/1 decompensat*):ti,ab	**6759**
#2	'cardiac failure':ti,ab	**16881**
#1	'heart failure'/exp	**491308**

CINAHL (EBSCO) 26-09-2019

#	Query	Results
S22	S20 AND S21	610
S21	(MH "Systematic Review") OR (MH "Cochrane Library") OR TI Meta-Analysis OR AB Meta-Analysis OR TI Metaanalysis OR AB Metaanalysis OR (AB Medline AND AB Cochrane) OR TI Guideline* OR AB Guideline* OR TI “Guide Lines” OR AB “Guide Lines” OR TI Consensus OR TI Recommendation* OR TI Random* OR AB Random* OR TI “Controlled Trial” OR AB “Controlled Trial” OR TI “Technology Assessment” OR AB “Technology Assessment” OR TI “Technology Appraisal” OR AB “Technology Appraisal” OR TI HTA OR AB HTA OR TI Overview OR (TI Review AND TI Literature)	547,021
S20	S13 AND S19	2,297
S19	S14 OR S15 OR S16 OR S17 OR S18	13,923
S18	TI Re-Hospitali* OR AB Re-Hospitali*	504
S17	TI Rehospitali* OR AB Rehospitali*	2,196
S16	TI Readmitted OR AB Readmitted	2,020
S15	TI Readmission* OR AB Readmission*	12,455

For peer review only

S14	(MH "Readmission")	11,566
S13	S1 OR S2 OR S3 OR S4 OR S5 OR S6 OR S7 OR S8 OR S9 OR S10 OR S11 OR S12	35,130
S12	TI "Heart Failure" OR AB "Heart Failure"	44,414
S11	TI "Myocardial Decompensated" OR AB "Myocardial Decompensated"	173
S10	TI "Myocardial Decompensation" OR AB "Myocardial Decompensation"	7
S9	TI "Myocardial Failure" OR AB "Myocardial Failure"	44
S8	TI "Cardiac Decompensated" OR AB "Cardiac Decompensated"	386
S7	TI "Cardiac Decompensated" OR AB "Cardiac Decompensated"	0
S6	TI "Cardiac Decompensation" OR AB "Cardiac Decompensation"	98
S5	TI "Heart Decompensated" OR AB "Heart Decompensated"	1,597
S4	TI "Heart Decompensated" OR AB "Heart Decompensated"	0
S3	TI "Heart Decompensation" OR AB "Heart Decompensation"	8
S2	TI "Cardiac Failure" OR AB "Cardiac Failure"	1,227
S1	(MH "Heart Failure+")	36,791

Global Health (OVID) 26-09-2019

☐	# ▲	**Searches**	**Results**
☐	1	Cardiac Failure.ti,ab.	1058
☐	2	Heart Decompensat*.ti,ab.	1
☐	3	Cardiac Decompensat*.ti,ab.	38
☐	4	Myocardial Failure.ti,ab.	61
☐	5	Myocardial Decompensat*.ti,ab.	0
☐	6	Heart Failure.ti,ab.	9089
☐	7	or/1-6	10064
☐	8	Readmission*.ti,ab.	2050
☐	9	Readmitted.ti,ab.	1113
☐	10	Rehospitaliz*.ti,ab.	310
☐	11	Re-Hospitaliz*.ti,ab.	121
☐	12	or/8-11	3203
☐	13	7 and 8	158

Estrategias Heart Failure Bundles

PubMed 24-09-2019

For peer review only

	Query	Items found
#14	**(#13 NOT Branch[tiab])**	319
#13	**(#4 AND #12)**	2109
#12	**(#5 OR #6 OR #7 OR #8 OR #9 OR #10 OR #11)**	204129
#11	**Heart Failure[tiab]**	163313
#10	**Myocardial Decompensat*[tiab]**	33
#9	**Myocardial Failure[tiab]**	757
#8	**Cardiac Decompensat*[tiab]**	1155
#7	**Heart Decompensat*[tiab]**	122
#6	**Cardiac Failure[tiab]**	11758
#5	**Heart Failure[MeSH]**	115710
#4	**(#1 OR #2 OR #3)**	62341
#3	**Bundles[tiab]**	27534
#2	**Bundle[tiab]**	38958
#1	**Patient Care Bundles[MeSH]**	662

EMBase (Elsevier) 24-09-2019

No.	Query	Results
#14	#13 NOT branch:ti,ab	**1009**
#13	#4 AND #12	**5474**
#12	#5 OR #6 OR #7 OR #8 OR #9 OR #10 OR #11	**536741**
#11	(myocardial NEAR/1 decompensat*):ti,ab	**57**
#10	'myocardial failure':ti,ab	**934**
#9	(cardiac NEAR/1 decompensat*):ti,ab	**1917**
#8	(heart NEAR/1 decompensat*):ti,ab	**6757**
#7	'cardiac failure':ti,ab	**16879**
#6	'heart failure':ti,ab	**259558**
#5	'heart failure'/exp	**491184**
#4	#1 OR #2 OR #3	**78092**
#3	bundles:ti,ab	**31971**
#2	bundle:ti,ab	**51066**
#1	'care bundle'/exp	**1150**

CINAHL (EBSCO) 24-09-2019

For peer review

#	Query	Results
S13	S12 NOT (TI Branch OR AB Branch)	74
S12	S3 AND S11	548
S11	S4 OR S5 OR S6 OR S7 OR S8 OR S9 OR S10	34,961
S10	TI "Heart Failure" OR AB "Heart Failure"	44,368
S9	TI "Myocardial Decompensation" OR AB "Myocardial Decompensation"	7
S8	TI "Myocardial Failure" OR AB "Myocardial Failure"	44
S7	TI "Cardiac Decompensation" OR AB "Cardiac Decompensation"	98
S6	(TI "Heart Decompensation" OR AB "Heart Decompensation") OR (TI "Heart Decompensated" OR "Heart Decompensated")	8
S5	TI "Cardiac Failure" OR AB "Cardiac Failure"	1,227
S4	(MH "Heart Failure+")	36,787
S3	S1 OR S2	6,601
S2	TI Bundles OR AB Bundles	6,463
S1	TI Bundle OR AB Bundle	6,572

Cochrane Library (Wiley) 24-09-2019

ID	Search	Hits
#1	MeSH descriptor: [Patient Care Bundles] explode all trees	21
#2	Bundle:ti,ab,kw	1731
#3	Bundles:ti,ab,kw	354
#4	#1 OR #2 OR #3	1968
#5	MeSH descriptor: [Heart Failure] explode all trees	8355
#6	(Cardiac NEAR/1 Failure):ti,ab,kw	993
#7	(Heart NEAR/1 Decompesat*):ti,ab,kw	1
#8	(Cardiac NEAR/1 Decompesat*):ti,ab,kw	0
#9	(Myocardial NEAR/1 Failure):ti,ab,kw	118
#10	(Myocardial NEAR/1 Decompensat*):ti,ab,kw	1
#11	(Heart NEAR/1 Failure):ti,ab,kw	27302
#12	#5 OR #6 OR #7 OR #8 OR #9 OR #10 OR #11	27757
#13	#4 AND #12	338
#14	#13 NOT Branch:ti,ab,kw	32

Global Health (OVID) 26-09-2019

☐	# ▲	Searches	Results
☐	1	Bundle.ti,ab.	1822
☐	2	Bundles.ti,ab.	1467
☐	3	1 or 2	3054
☐	4	Cardiac Failure.ti,ab.	1058
☐	5	Heart Decompensat*.ti,ab.	1
☐	6	Cardiac Decompensat*.ti,ab.	38

For peer review only

☐	7	Myocardial Failure.ti,ab.	61
☐	8	Myocardial Decompensat*.ti,ab.	0
☐	9	Heart Failure.ti,ab.	9089
☐	10	or/4-9	10064
☐	11	3 and 10	66
☐	12	11 not Branch.ti,ab.	17

For peer review only

Supplemental Annex 2. Flowcharts

Flow chart for literature review of HF interventions

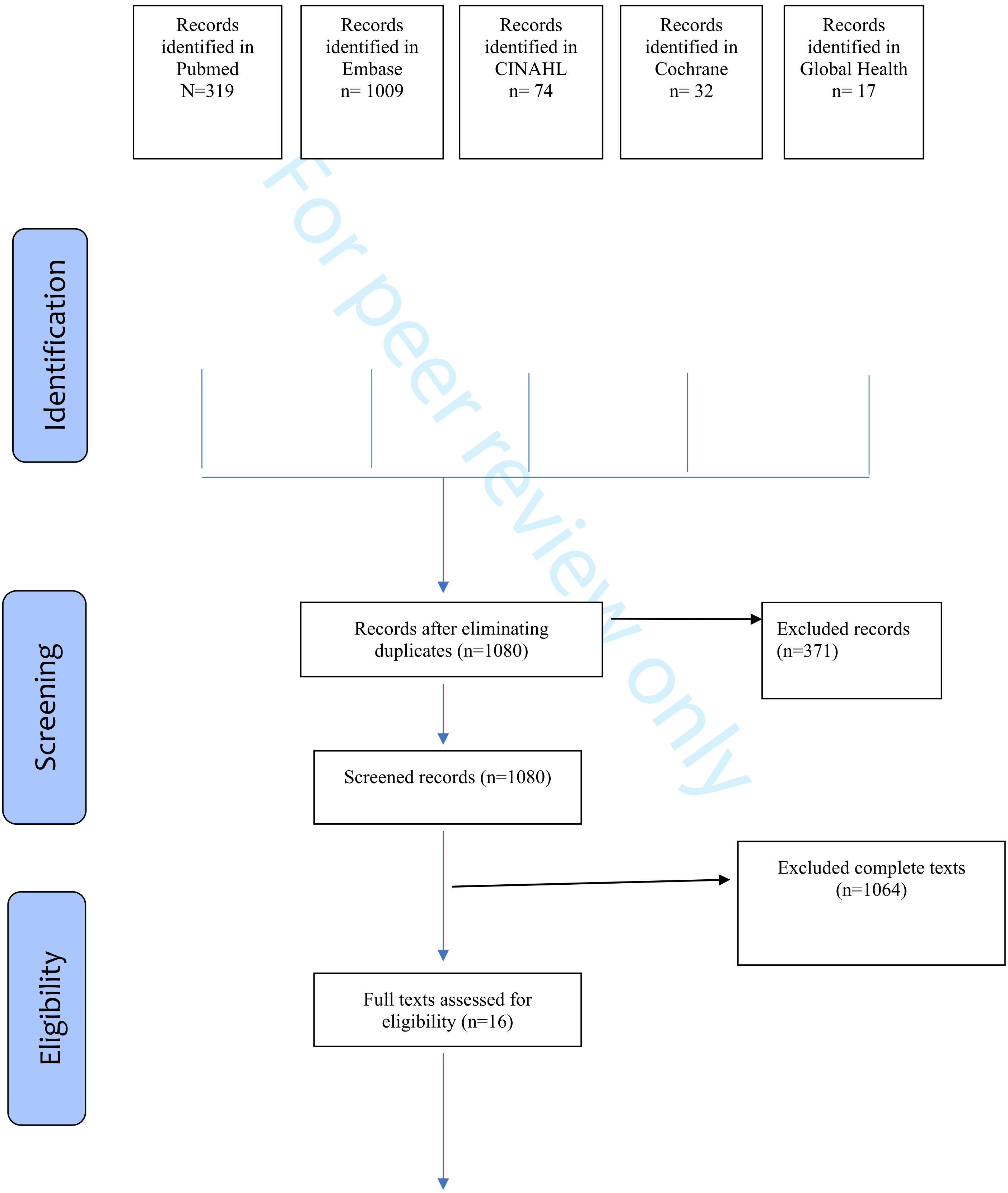

For peer review only

Flow chart for literature review of Readmission

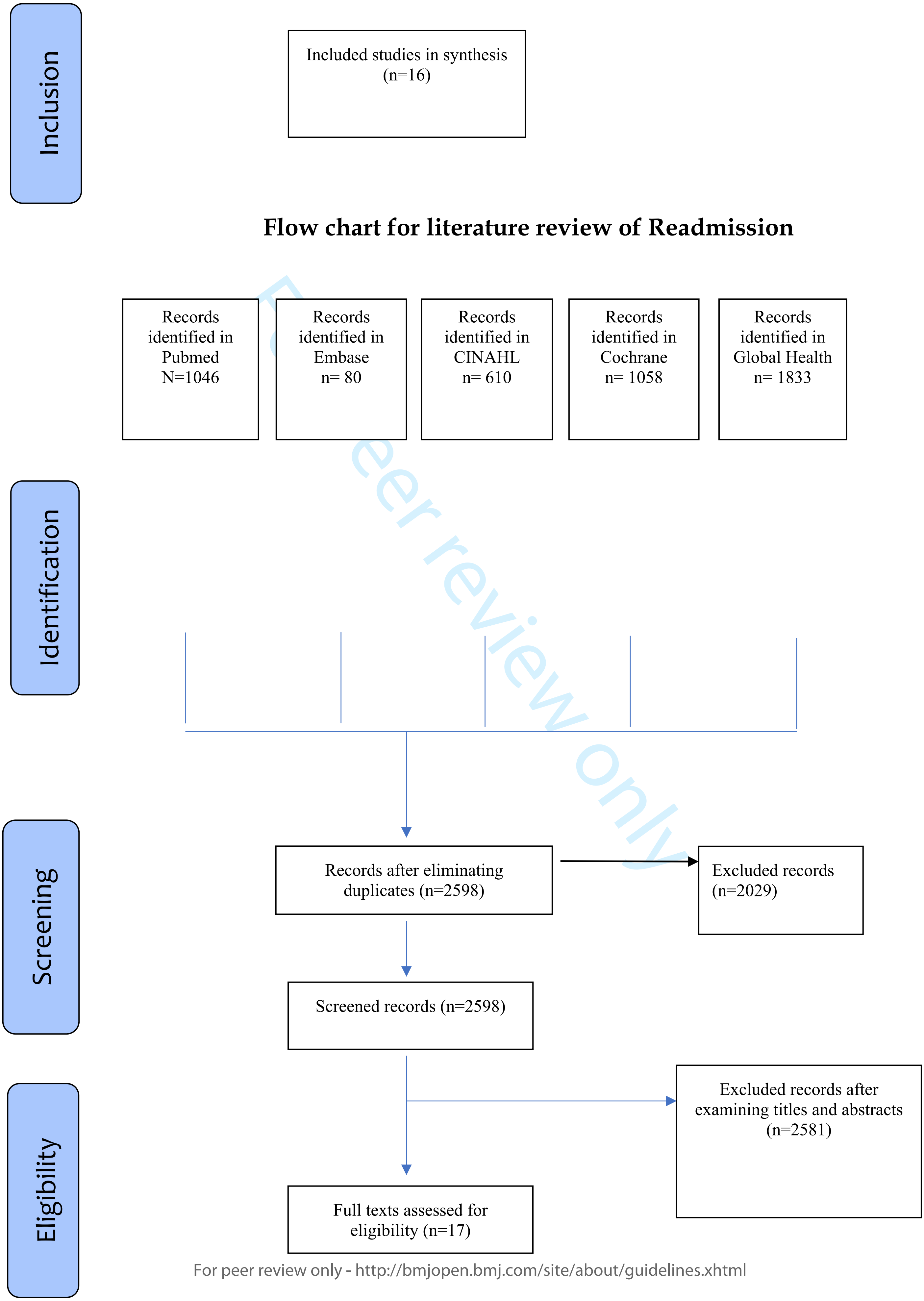

For peer review only

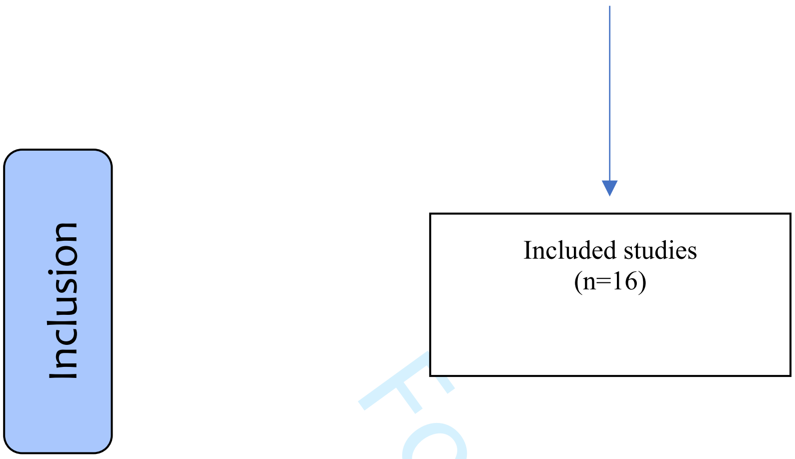

For peer review only

BMJ Open

Care Bundle to Reduce Readmission in Patients with Heart Failure: A Modified Delphi Consensus Panel in Argentina

Journal:	*BMJ Open*
Manuscript ID	bmjopen-2020-040028.R1
Article Type:	Original research
Date Submitted by the Author:	14-Nov-2020

Complete List of Authors:	Roberti, Javier; Universidad Nacional de Entre Rios; Institute for Clinical Effectiveness and Health Policy<br>Vita, Tomas; Institute for Clinical Effectiveness and Health Policy<br>Piastrella, Jimena; Novartis Argentina SA<br>Porley, Carlos; Novartis Argentina SA<br>Pereyra, Lisandro; Novartis Argentina SA<br>Diez, Mirta; Instituto Cardiovascular de Buenos Aires<br>Renedo, Florencia; Fundacion Favaloro Hospital Universitario<br>Fairman, Enrique; Clinica Bazterrica<br>Fernandez, Alberto; Sanatorio Modelo Quilmes<br>Thierer, Jorge; CEMIC<br>Garcia Elorrio, Ezequiel; Institute for Clinical Effectiveness and Health Policy

**Primary Subject Heading**:	Cardiovascular medicine
Secondary Subject Heading:	Cardiovascular medicine
Keywords:	Adult cardiology < CARDIOLOGY, Heart failure < CARDIOLOGY, Quality in health care < HEALTH SERVICES ADMINISTRATION & MANAGEMENT, Protocols & guidelines < HEALTH SERVICES ADMINISTRATION & MANAGEMENT

SCHOLARONE™
Manuscripts

I, the Submitting Author has the right to grant and does grant on behalf of all authors of the Work (as defined in the below author licence), an exclusive licence and/or a non-exclusive licence for contributions from authors who are: i) UK Crown employees; ii) where BMJ has agreed a CC-BY licence shall apply, and/or iii) in accordance with the terms applicable for US Federal Government officers or employees acting as part of their official duties; on a worldwide, perpetual, irrevocable, royalty-free basis to BMJ Publishing Group Ltd ("BMJ") its licensees and where the relevant Journal is co-owned by BMJ to the co-owners of the Journal, to publish the Work in this journal and any other BMJ products and to exploit all rights, as set out in our licence.

The Submitting Author accepts and understands that any supply made under these terms is made by BMJ to the Submitting Author unless you are acting as an employee on behalf of your employer or a postgraduate student of an affiliated institution which is paying any applicable article publishing charge ("APC") for Open Access articles. Where the Submitting Author wishes to make the Work available on an Open Access basis (and intends to pay the relevant APC), the terms of reuse of such Open Access shall be governed by a Creative Commons licence – details of these licences and which Creative Commons licence will apply to this Work are set out in our licence referred to above.

Other than as permitted in any relevant BMJ Author's Self Archiving Policies, I confirm this Work has not been accepted for publication elsewhere, is not being considered for publication elsewhere and does not duplicate material already published. I confirm all authors consent to publication of this Work and authorise the granting of this licence.

For peer review only

Care Bundle to Reduce Readmission in Patients with Heart Failure: A Modified Delphi Consensus Panel in Argentina

Javier Roberti[1,2*], Tomás Vita[1], Jimena Piastrella[3], Carlos Porley[3], Lisandro Pereyra[3] Mirta Diez[4], Florencia Renedo[5], Enrique Fairman[6], Alberto Fernández[7], Jorge Thierer[8], Ezequiel García Elorrio[1]

1. Institute for Clinical Effectiveness and Health Policy (IECS), Buenos Aires, Argentina
2. National Scientific and Technical Research Council (CONICET)- Argentina
3. Novartis Argentina, Buenos Aires, Argentina
4. Instituto Cardiológico Buenos Aires – ICBA, Buenos Aires, Argentina
5. Fundación Favaloro, Buenos Aires, Argentina
6. Clínica Bazterrica, Buenos Aires, Argentina
7. Clínica Modelo Quilmes, Quilmes, Argentina
8. CEMIC, Buenos Aires, Argentina

***Corresponding Author**

Javier Roberti, Email: jroberti@iecs.org.ar

For peer review only

Abstract

Objectives. The aim of this study was to develop consensus among Argentine cardiologists on a care bundle to reduce readmissions in patients with heart failure (HF).

Setting: Hospitals and cardiology clinics in Argentina providing in-hospital care for patients with HF.

Participants: Twenty-four cardiology experts in cardiology participated in the two online rounds and 18 (75%) of them participated in the third-round meeting.

Methods: This study used a mixed method design and was conducted between August 2019 and January 2020. The approach used to develop a care bundle (a set of evidence-based interventions applied together to improve clinical outcomes) involved three phases: a) a literature review to define the list of interventions that would be evaluated; b) a modified Delphi panel to select interventions for the bundle; finally, c) development of the HF care bundle. The process included three rounds of scoring.

Results: Twenty-six interventions were evaluated. The proposed bundle was based on four categories: medication, continuum of care, lifestyle habits, predischarge tests. Medication: Beta-blockers, angiotensin receptor neprilysin inhibitors or ACE-inhibitors, furosemide and anti-mineralocorticoids; Continuum of care: follow-up appointment, daily weight monitoring; Lifestyle habits: smoking cessation counseling and low sodium diet; pre-discharge tests: renal function, ionogram, blood pressure control, echocardiogram and determination of decompensating cause.

Conclusion: Following a systematic mixed method approach, we have developed a care bundle of interventions that could decrease readmission of patients with HF. The application of this bundle could contribute to scale evidence-based interventions.

Article summary

Strengths and limitations of this study

For peer review only

- Potential interventions were chosen through a systematic review
- Cardiologist experts participated in a transparent consensus process
- Consensus was reached through Delphi process
- As in most consensus, participants could have misinterpreted statements
- Potential bias from cardiologists as only specialty involved in process.

Funding statement: This work was supported by Novartis Argentina SA, grant number number NCC CLCZ696BAR05R

Introduction

Despite several medical advances to treat heart failure (HF), mortality and hospital readmission have not changed significantly.[1] Treatment adherence, diet, exercise, regular monitoring, and paperwork demanded by the health system place a significant burden on patients and their caregivers.[2,3] Moreover, a high percentage of patients with HF are not receiving adequate treatment despite the increased use of evidence-based therapy and enhanced adherence to performance measures.[4-6] Several initiatives have confirmed to diminish HF readmission; however, they are challenging to replicate. In this context, the use of a care bundle with additional strategies such as quality improvement collaboratives (QICs) to scale-up its use could contribute to the optimization of the treatment of patients with HF.[7,8]

A care bundle is defined as a set of evidence-based interventions, called elements, that should be applied together in every eligible patient to enhance the reliability of care and

For peer review only - http://bmjopen.bmj.com/site/about/guidelines.xhtml

to improve clinical outcomes.[9,10] The completion of the interventions of a bundle should be measured as all-or-nothing; when all components performed collectively and reliably, they have shown to improve patient outcomes.[11] A care bundle approach for HF should focus on providing evidence-based clinical practice, engaging patients and careers as active partners, and creating processes to ensure a quality handoff from hospital care.

Hospitalizations in HF are the main trigger for treatment interventions; however, financers' lack of awareness of the clinical burden and the low urgency to intervene in these patients compared to other cardiovascular diseases represent significant challenges for implementation.[12,13] In fact, HF guidelines recommend initiating and uptitrating disease-modifying therapies during hospitalization.[14] As the preliminary phase of a future QIC, the aim of this study was to develop consensus among Argentine cardiologists on a care bundle to reduce readmissions in patients with HF.

Methods

This study used a mixed method design and was conducted between August 2019 and January 2020. The approach used to develop a care bundle involved three phases: a) a literature review to define the list of interventions that would be evaluated; b) a modified Delphi panel to select interventions for the bundle; finally, c) development of the HF care bundle. The process had seven steps, including three rounds of scoring. See Figure 1 for an illustration of the study design.

(Figure 1)

For peer review only

Phase 1.

Step 1. Explorative review of the literature

In preparation of the Delphi questionnaire, a review of the literature was performed. We searched in PubMed, LILACS, EMBASE, The Cochrane Library and Google Scholar for relevant literature. No HF care bundle was identified in the literature; a review of gray literature showed isolated experiences shared over the internet. We included articles describing interventions for HF, with special attention to those specified in the Guidelines of the American College of Cardiology (ACC), the American Heart Association (AHA), the European Society of Cardiology (ESC) and the Argentine Cardiology Society.[7,8,15] We used a pragmatic explorative approach. Supplemental annex 1 shows search strategies used; supplemental annex 2 shows the PRISMA flowchart of selection process. Based on the literature, we proposed a list of 26 interventions to be assessed by experts. Table 1 shows the 26 interventions selected for evaluation.

(Table 1)

Step 2. Developing the list of interventions

The target patient for the interventions was an individual with signs and symptoms of HF and an ejection fraction lower than 40% that underwent a hospitalization and was discharged during the next 48 hours. Based on the literature, we proposed a list of 26 interventions to be assessed by experts. To guide the selection of interventions during the consensus process, we used the criteria proposed by GRADE from *Evidence to Decision* framework validated and adopted by WHO (project EtD / DECIDE) and others from the care package literature to help panels of experts use evidence in a structured

For peer review only - http://bmjopen.bmj.com/site/about/guidelines.xhtml

and transparent way to inform decisions in the context of healthcare recommendations.[10] The potential interventions were presented to panelists as short statements with references to supporting literature. For each statement, panelists were asked to rate interventions by 13 criteria or parameters using a nine-point Likert scale ranging from 1 (extremely inappropriate / insignificant) to 9 (extremely appropriate/ significant).[16] The 13 criteria were 1) priority, 2) unintended effects, 3) intended effects, 4) balance between unintended and intended effects, 5) quality of evidence on effects, 6) values and preferences, 7) resource required, 8) quality of evidence on resources, 9) cost-effectiveness, 10) equity, 11) acceptability, 12) feasibility, and 13) measurability. The EtD framework ensures that panels consider important criteria, structure discussion and identify reasons for disagreements, based on the balance of health benefits and harms, human rights and sociocultural acceptability, health equity, equality and non-discrimination, societal implications, financial and economic considerations, feasibility and health system considerations and the meta-criterion quality of evidence.[17,18]

Phase 2. Consensus on a care bundle.

Step 1. Selection of experts' panel

We used a modified RAND-UCLA Delphi method to select interventions for HF care bundle to be applied during the discharge process care to prevent readmissions, where an expert panel members use their professional judgment alongside the best available evidence to identify areas where consensus can be reached for the topic under consideration.[16]

For peer review only

In the second stage, 26 experts in chronic HF were selected from different clinical contexts in Argentina, mainly from the high-density area of Buenos Aires. All participants were specialists in cardiology, needed to have proven experience in treating patients with HF and in scientific research on HF, currently practicing in hospitals which had a cardiology fellowship, at least 80 beds, and with 3,000 admissions or more per year. These physicians were known to the team for their involvement in HF treatment and research and were recommended by first participants to be selected. Twenty-six potential participants were sent a recruitment letter and information sheet via e-mail, follow-up telephone calls were made to confirm reception of material. Of the total, 24 (92%) professionals accepted to participate. Panelists were given a small honorarium for their time and effort.

Step 2. Interventions rating

Permanent disagreement was defined as at least six panelists rating the indication in the 1-3 region, and at least six panelists rating it in the 7-9 region after three rounds of scoring. Each intervention obtained 13 scores, one for each parameter or criterion.[16] Then, this score was multiplied by the weighting factor obtained in the rating of the parameters and mean was calculated for each intervention. Interventions were then sorted according to this global score.

Step 3. First round online survey

For the first round, the experts received a link for the online questionnaire (surveymonkey.com, San Mateo, California, USA). Twenty-six interventions in 13 criteria were evaluated for disagreement following the classic RAND definition of at least six

For peer review only - http://bmjopen.bmj.com/site/about/guidelines.xhtml

panelists rating the parameter in the 1-3 region and at least six panelist rating it in the 7-9 region.[16] Security sockets layer (SSL) encryption is used to protect data while being transmitted by ensuring secure connections between participants and the server. An email was issued to each participant requesting them to open the survey monkey tool, answer the questions and press the submit button. All participants had the contact details for the research team, who they could contact if they had any doubts regarding the materials provided. Participants had 10 days to complete the survey, with a reminder email sent to non-responders.

Step 4. Second online survey round

In round two, all interventions and their criteria that did not reach an agreement in the first round were included for a new vote. Participants were shown the whole group's ratings as frequency data on a rating scale for each item, alongside their own ratings for each statement from round one. They could revise their own original rating considering the group ratings if they so wished. Participants had 10 days to complete round two with a reminder sent to non-responders.

Step 5. Consensus meeting

All participants of the online survey were invited via email and telephone to a three-hour in person consensus meeting. The main objectives of the meeting were to discuss the indications that have been rated with disagreement and inconsistencies in appropriateness ratings. Panelists were encouraged to refer to the literature. Of the total of participants who responded the online rounds, 18 (75%) took part in the meeting. During the session, results of the second online survey were presented and discussed,

For peer review only

followed by an anonymous onsite rating of those items that had showed disagreement on previous round. In addition, the 13 quality criteria were anonymously rated using a 1-9 Likert scale to obtain a factor for final evaluation of each intervention. The panel mean score was calculated for each statement and classified into three categories: 1–3.5 (potentially inappropriate); 3.6– 6.4 (uncertain), and 6.5–9 (potentially appropriate). The moderator was a physician, not a specialist in the area being rated to avoid bringing their bias to the discussion, he had led the literature review and was familiar with the material.

Step 6. Development of HF care bundle with expert committee

We invited five of the participants of previous rounds with extensive expertise to join an expert committee to discuss the results of the survey and rate the bundle. The results of the whole process were presented in this meeting. The expert committee selected those interventions with the highest scores for the final evaluation and no disagreements. Based on these results, a final bundle of interventions was developed.

Ethical aspects and role of funding source

Written informed consent was obtained from all participants included in the study, and all procedures were conducted according to the Declaration of Helsinki. The founder of the study did not intervene in any stage of the consensus process. This study was evaluated and approved by an independent ethics committee, Independent Ethics Committee for Clinical Pharmacology Trials Prof Luis M. Zieher, Buenos Aires, Argentina, protocol number 0042/19v.05 on 17 October 2019

For peer review only

Patient and Public Involvement

The research question addresses a most significant issue for patients and was informed by a review of the literature on patients' journey when admitted for heart failure care. Only physicians with a vast clinical experience participated in the consensus process; these participants brought patients' concerns and perspectives into the process. The results of this study are disseminated via open access publications. This study is complemented by a qualitative research on patients' perspectives, reported separately.

Results

Twenty-four experts participated in the two online rounds and 18 (75%) of them participated in the third-round meeting. Eight (33.3%) were female; 16 (66.7%) participants worked in the private sector, 2 (8.3%) in the public sector and 6 (25%) in both sectors. Sixteen (66.7%) participants worked in the metropolitan area of Buenos Aires, while the rest, 8 (33.3%), worked in other provinces of Argentina. The median of professional experience was 26.5 years (range, 9 – 40 years).

Twenty-six interventions were evaluated, and each intervention obtained a global score. After the three rounds, consensus was reached for all interventions except for one intervention under one criterion (unintended effects of telephone tracking of patients). The mean global scores of 13 interventions were above the cutoff score of 6.5 and seven of these scored a median of 7.1 (Supplemental table 1). Of the 13 final interventions, 5 (38%) were related to medications, 2 (15%) were diagnostic tests, and 2 (15%) were habit recommendations (hyposodic diet and daily weight monitoring). The two most valuable criteria ranked by experts were priority and desirable effects of the intervention.

For peer review only

Administration of ACE-inhibitors, renal function and ionogram evaluation and blood pressure control were considered the most important interventions. The use of beta-blockers was the fifth intervention in the ranking of potential interventions while the use of anti-mineralocorticoids, furosemide and angiotensin receptor neprilysin inhibitor (ARNI) were ninth, tenth and eleventh, respectively. The use of ARNI ranked better on undesirable effects than the administration of furosemide and anti-mineralocorticoids. The administration of ARNI and the use of ACE-inhibitors obtained the same scores in six criteria; mainly related to scientific evidence evaluation; of note under the criteria that evaluated equity, ACE-inhibitors ranked higher. Smoking cessation and blood pressure control were ranked better than the use of medication because, unlike medication, the first two had no potential undesirable effects according to participants.

Daily weight monitoring and a follow-up appointment within a week of discharge were fourth and seventh, respectively. While echocardiography during hospitalization and determining the cause of decompensation were the interventions positioned last in the list of elements that could be included into the bundle, above the established cut-off point. Measurement of biomarker pro-BNP and BNP ranked low and did not make the cutoff. Physical exercise and cardiac resynchronization therapy did not rank high for the overall goal of reducing hospital readmission.

Proposed Bundle

The proposed bundle was based on four categories of interventions: medication, continuum of care, lifestyle habits, predischarge tests (Table 2). Beta-blockers, ARNI or ACE-inhibitors, furosemide and anti-mineralocorticoids were included as interventions

related to pharmacologic treatment. ARNI or ACE-inhibitors can be used interchangeably. The election would depend on clinician judge and patient choice. Follow-up appointment with a specialist within a week of discharge and daily weight monitoring were also included as interventions that reduce 30-day hospital readmission under "continuum of care" category, while smoking cessation counseling and low sodium diet were interventions classified as lifestyle habits. The interventions included under pre discharge tests were renal function and ionogram evaluation, blood pressure control, echocardiogram during hospitalization and determination of the decompensating cause.

(Table 2)

Discussion

Following a validated and systematic consensus method, we created a care bundle of interventions that could contribute to the management of HF and prevent readmission within the 30 days after discharge. The reduction of readmissions for patients with HF represents a challenge due to various factors such as a lack of targeted therapy for patients with preserved ejection fraction, the natural history of HF, the need for daily management to avoid decompensations, and problems related to access to care, health literacy, and other socioeconomic factors.[19] HF is a complex syndrome and the result of congestion and/or poor perfusion leads to negative effects on multiple organ systems. In fact, patients with HF have multiple comorbidities, a long list of medications, and symptoms that may be attributed to several etiologies.[19]

Several studies have evaluated single interventions to improve outcomes in patients with HF; ESC, ACC and HF Argentinean guidelines have established recommendations, but

interventions considered critical have not been specified.[7,8,15]. For participants, the intervention's priority and its desirable effects were the two most important criteria to evaluate each intervention. Probably, these two criteria are what cardiologists in the local context refer to when assessing any treatment in time-constrained settings. We have defined priorities in a time constrained setting as it is usually the case with clinical work. As all the potential interventions had been recommended on HF guidelines, no significant differences in global scores were observed among those above the cut-off point. Indeed, when the results were shown to the experts, all of them agreed on the significance of the interventions that finally included in the bundle.

Almost half of the interventions were related to medication, which, with robust evidence supporting its efficacy, is widely accepted as the backbone of HF treatment. It has been shown that interventions to improve adherence to medications for HF significantly reduce the risk for both hospitalization and death.[4] Interestingly, the use of beta-blockers was only fifth on the interventions ranking although beta-blockers have been confirmed to reduce mortality, readmission and improve symptomatology.[20-23] It is possible that the use of beta-blockers was not chosen as the most important interventions in our study as many patients have HF in very advanced stages and require inotropes during hospitalization while beta-blockers are administered during outpatient care. Clinical trials of ARNI showed a significant reduction in mortality and readmission when compared to ACE inhibitors.[24,25] The administration of ACE-inhibitors was pondered as the most important intervention and, at a granular level, the difference between ARNI and ACE-inhibitors was based mainly on costs and access. The high cost of the drug in an economically constrained setting explains the gap between evidence and real-world practice. Due to

For peer review only - http://bmjopen.bmj.com/site/about/guidelines.xhtml

new evidence in recent years, the final recommendations in the bundle allow the physician to decide between ARNI or ACE-inhibitors.[24,25]

Interestingly, many of the interventions were not strictly clinical issue such as daily weight monitoring or a follow-up appointment; reflecting the importance of an integrated care and the complex setting in which professionals and the health system have to interact to achieve the best quality outcomes; moreover, in a follow-up appointment, the patient could be provided with information to prevent re-admission. Importantly, laboratory examinations such as tests of renal function and the ionogram, obtained a high score and valued as the second most important intervention. Of the five medications included in the bundle, four could negatively affect renal function. Also, in the Argentinian health system, BNP test is expensive and is not covered by most health insurances which could explain the fact that this test was not included in the final list of elements.

In pioneering care bundles, two components were essential to their success; participating clinicians agreed that there was sufficient medical evidence supporting each individual element in the bundle to recommend that it be applied to most, if not all, patients; at a minimum, each element should be considered for every patient.[26,29] Second, the list of elements in the bundle should not be extensive. The goal of the bundle approach is to develop a short list of interventions that are already recommended and accepted in guidelines; this allows improvement rather than a debate on validity of the elements. It is essential that elements have the consensus of local clinicians.[30] On the other hand, checklists and protocols serve to augment memory and limit the chance of human error, improving communication, standardizing responses, reducing unnecessary clinical variation.[31]

For peer review only

Our study has limitations that should be acknowledged. Participants could have misinterpreted the original statements describing the potential interventions and the same applies to criteria definitions. We tried to control this factor by providing participants with information and discussing definitions during the meeting. An important limitation is related to the method itself; our results reflect the opinion of selected cardiology specialists with a vast experience. However, cardiologists see most of HF patients and once the bundle applicability is assessed, it could be disseminated to other contexts that include other specialties. The interventions were not tested in community or primary care but this bundle is intended to be used during patient hospitalization, before discharge. Also, we disclose the funding by a pharmaceutical company and the participation of professionals affiliated to this company, which could be interpreted as a limitation. We have made every effort to maintain the transparency of the process with the participation of professionals in every stage.

In conclusion, following a mixed method approach we developed a care bundle of interventions that could decrease readmission of patients with HF. Our next step will be to study the implementation of this bundle in several sites. The application of this bundle could be used to scale evidence-based interventions under the framework of Quality Improvement Learning Collaborative.

Acknowledgements

We would like to thank the participation of the following health professionals: José Luis Barisani (Clínica Adventista), Roberto Colque (Sanatorio Allende), Marcelo Crespo (Sanatorio Los Arcos), Clara Huerta (Hospital Córdoba), Juan Pablo Escalante (Instituto

For peer review only - http://bmjopen.bmj.com/site/about/guidelines.xhtml

Cardiovascular Rosario), David Flores (Hospital Nacional de Clínicas), Jimena Gambarte (Hospital Alemán), Daniela García Brasca (Hospital Italiano de Córdoba), Alfredo Larraburu (Sanatorio Los Arcos), Adrián Lescano (Centro Gallego), Javier Marino (ICBA), Lidia Lobo Márquez (Instituto de Cardiología), Cristian Mastantuono (Hospital Pirovano), Felipe Martínez (Universidad Nacional de Córdoba), Stella Pereiro (Hospital Churruca), Eduardo Perna (Instituto de Cardiología de Corrientes Juana Francisca Cabral), José Santucci (Hospital Austral), Julieta Soricetti (Hospital Durand), Ezequiel Zaidel (Sanatorio Güemes).

A. **Contributorship statement:** Javier Roberti: Methodology, investigation, analysis, writing. Tomas Vita: investigation, analysis, writing. Jimena Piastrella, Carlos Porley, and Lisandro Pereyra: investigation, interpretation, review; Mirta Diez, Florencia Renedo, Enrique Fairman, Alberto Fernández, Jorge Thierer: investigation, interpretation, review; Ezequiel García Elorrio: concept, methodology, analysis, interpretation, review.

B. **Competing interests:** Jimena Piastrella, Carlos Porley, and Lisandro Pereyra are employees of Novartis Argentina SA, funder of this study. Javier Roberti, Tomas Vita, Florencia Renedo, Enrique Fairman, Alberto Fernandez, Jorge Thierer and Ezequiel Garcia Elorrio have no competing interest to declare.

C. **Funding**: This study was supported by Novartis Argentina SA (grant number NCC CLCZ696BAR05R).

D. **Data sharing statement**: Supplementary data such as search strategies, flowcharts and table of scores are available in supplementary materials and upon request.

References

1. Bytyci I, Bajraktari G. Mortality in heart failure patients. Anatol J Cardiol. 2015;15(1):63-8. Epub 2015/01/01. doi: 10.5152/akd.2014.5731.

2. Nordfonn OK, Morken IM, Bru LE, Husebo AML. Patients' experience with heart failure treatment and self-care-A qualitative study exploring the burden of treatment. J Clin Nurs. 2019;28(9-10):1782-93. Epub 2019/01/23. doi: 10.1111/jocn.14799.

3. Evangelista LS, Shinnick MA. What do we know about adherence and self-care? J Cardiovasc Nurs. 2008;23(3):250-7. Epub 2008/04/26. doi: 10.1097/01.JCN.0000317428.98844.4d.

4. Ruppar TM, Cooper PS, Mehr DR, Delgado JM, Dunbar-Jacob JM. Medication Adherence Interventions Improve Heart Failure Mortality and Readmission Rates: Systematic Review and Meta-Analysis of Controlled Trials. J Am Heart Assoc. 2016;5(6). Epub 2016/06/19. doi: 10.1161/JAHA.115.002606.

5. Fonarow GC, Abraham WT, Albert NM, Gattis Stough W, Gheorghiade M, Greenberg BH, et al. Influence of a performance-improvement initiative on quality of care for patients hospitalized with heart failure: results of the Organized Program to Initiate Lifesaving Treatment in Hospitalized Patients With Heart Failure (OPTIMIZE-HF). Arch Intern Med. 2007;167(14):1493-502. doi: 10.1001/archinte.167.14.1493.

6. Panella M, Marchisio S, Demarchi ML, Manzoli L, Di Stanislao F. Reduced in-hospital mortality for heart failure with clinical pathways: the results of a cluster randomised controlled trial. Qual Saf Health Care. 2009;18(5):369-73. doi: 10.1136/qshc.2008.026559.

For peer review only

7. Ponikowski P, Voors AA, Anker SD, Bueno H, Cleland JG, Coats AJ, et al. 2016 ESC Guidelines for the diagnosis and treatment of acute and chronic heart failure: The Task Force for the diagnosis and treatment of acute and chronic heart failure of the European Society of Cardiology (ESC) Developed with the special contribution of the Heart Failure Association (HFA) of the ESC. Eur Heart J. 2016;37(27):2129-200. doi: 10.1093/eurheartj/ehw128.

8. Yancy CW, Jessup M, Bozkurt B, Butler J, Casey DE, Jr., Colvin MM, et al. 2017 ACC/AHA/HFSA Focused Update of the 2013 ACCF/AHA Guideline for the Management of Heart Failure: A Report of the American College of Cardiology/American Heart Association Task Force on Clinical Practice Guidelines and the Heart Failure Society of America. J Am Coll Cardiol. 2017;70(6):776-803. doi: 10.1016/j.jacc.2017.04.025.

9. Lavallee JF, Gray TA, Dumville J, Russell W, Cullum N. The effects of care bundles on patient outcomes: a systematic review and meta-analysis. Implement Sci. 2017;12(1):142. doi: 10.1186/s13012-017-0670-0.

10. Resar R, Griffin F, Haraden C, Nolan T. Using Care Bundles to Improve Health Care Quality. Cambridge, Massachusetts: Institute for Healthcare Improvement;, 2012.

11. Agency for Healthcare Research and Quality. Eliminating CLABSI: A National Patient Safety Imperative. Final Report. Rockville, MD: 2013. Available online at https://www.ahrq.gov/hai/cusp/clabsi-final/index.html. Accessed 10 December 2019.

12. Arora S, Patel P, Lahewala S, Patel N, Patel NJ, Thakore K, et al. Etiologies, Trends, and Predictors of 30-Day Readmission in Patients With Heart Failure. Am J Cardiol. 2017;119(5):760-9. Epub 2017/01/23. doi: 10.1016/j.amjcard.2016.11.022.

For peer review only

13. Wan TTH, Terry A, Cobb E, McKee B, Tregerman R, Barbaro SDS. Strategies to Modify the Risk of Heart Failure Readmission: A Systematic Review and Meta-Analysis. Health Serv Res Manag Epidemiol. 2017;4:2333392817701050. Epub 2017/05/04. doi: 10.1177/2333392817701050.

14. Seferovic PM, Ponikowski P, Anker SD, Bauersachs J, Chioncel O, Cleland JGF, et al. Clinical practice update on heart failure 2019: pharmacotherapy, procedures, devices and patient management. An expert consensus meeting report of the Heart Failure Association of the European Society of Cardiology. Eur J Heart Fail. 2019;21(10):1169-86. Epub 2019/05/28. doi: 10.1002/ejhf.1531.

15. Sociedad Argentina de Cardiología. Consenso de insuficiencia cardíaca crónica. Revista Argentina de Cardiología. 2016; 84(S3):3-50.

16. Fitch K, Bernstein SJ, Aguilar DM, Burnand B, LaCalle JR, Lazaro P, et al. The RAND/UCLA Appropriateness Method User's Manual. Santa Monica, CA: RAND Corporation, 2001. https://www.rand.org/pubs/monograph_reports/MR1269.html. Also available in print form

17. Moberg J, Oxman AD, Rosenbaum S, Schunemann HJ, Guyatt G, Flottorp S, et al. The GRADE Evidence to Decision (EtD) framework for health system and public health decisions. Health Res Policy Syst. 2018;16(1):45. Epub 2018/05/31. doi: 10.1186/s12961-018-0320-2.

18. Alonso-Coello P, Schunemann HJ, Moberg J, Brignardello-Petersen R, Akl EA, Davoli M, et al. [GRADE Evidence to Decision (EtD) frameworks: a systematic and transparent approach to making well informed healthcare choices. 1: Introduction]. Gac Sanit. 2018;32(2):166 e1- e10. Epub 2017/08/22. doi: 10.1016/j.gaceta.2017.02.010.

For peer review only

19. Goldgrab D, Balakumaran K, Kim MJ, Tabtabai SR. Updates in heart failure 30-day readmission prevention. Heart Fail Rev. 2019;24(2):177-87. Epub 2018/11/30. doi: 10.1007/s10741-018-9754-4.

20. Hjalmarson A, Goldstein S, Fagerberg B, Wedel H, Waagstein F, Kjekshus J, et al. Effects of controlled-release metoprolol on total mortality, hospitalizations, and well-being in patients with heart failure: the Metoprolol CR/XL Randomized Intervention Trial in congestive heart failure (MERIT-HF). MERIT-HF Study Group. JAMA. 2000;283(10):1295-302. doi: 10.1001/jama.283.10.1295.

21. Packer M, Coats AJ, Fowler MB, Katus HA, Krum H, Mohacsi P, et al. Effect of carvedilol on survival in severe chronic heart failure. N Engl J Med. 2001;344(22):1651-8. doi: 10.1056/NEJM200105313442201.

22. Packer M, Bristow MR, Cohn JN, Colucci WS, Fowler MB, Gilbert EM, et al. The effect of carvedilol on morbidity and mortality in patients with chronic heart failure. U.S. Carvedilol Heart Failure Study Group. N Engl J Med. 1996;334(21):1349-55. doi: 10.1056/NEJM199605233342101.

23. Effect of metoprolol CR/XL in chronic heart failure: Metoprolol CR/XL Randomised Intervention Trial in Congestive Heart Failure (MERIT-HF). Lancet. 1999;353(9169):2001-7.

24. McMurray JJ, Packer M, Desai AS, Gong J, Lefkowitz MP, Rizkala AR, et al. Angiotensin-neprilysin inhibition versus enalapril in heart failure. N Engl J Med. 2014;371(11):993-1004. doi: 10.1056/NEJMoa1409077.

For peer review only

25. Wachter R, Senni M, Belohlavek J, Straburzynska-Migaj E, Witte KK, Kobalava Z, et al. Initiation of sacubitril/valsartan in haemodynamically stabilised heart failure patients in hospital or early after discharge: primary results of the randomised TRANSITION study. Eur J Heart Fail. 2019;21(8):998-1007. doi: 10.1002/ejhf.1498.

26. Resar R, Pronovost P, Haraden C, Simmonds T, Rainey T, Nolan T. Using a Bundle Approach to Improve Ventilator Care Processes and Reduce Ventilator-Associated Pneumonia. The Joint Commission Journal on Quality and Patient Safety. 2005;31(5):243-8. doi: 10.1016/s1553-7250(05)31031-2.

27. Burger CD, Resar RK. "Ventilator bundle" approach to prevention of ventilator-associated pneumonia. Mayo Clin Proc. 2006;81(6):849-50. Epub 2006/06/15. doi: 10.4065/81.6.849.

28. Bird D, Zambuto A, O'Donnell C, Silva J, Korn C, Burke R, et al. Adherence to ventilator-associated pneumonia bundle and incidence of ventilator-associated pneumonia in the surgical intensive care unit. Arch Surg. 2010;145(5):465-70. Epub 2010/05/19. doi: 10.1001/archsurg.2010.69.

29. Furuya EY, Dick A, Perencevich EN, Pogorzelska M, Goldmann D, Stone PW. Central line bundle implementation in US intensive care units and impact on bloodstream infections. PLoS One. 2011;6(1):e15452. Epub 2011/01/27. doi: 10.1371/journal.pone.0015452.

30. Resar R, Griffin F, Haraden C, Nolan T. Using Care Bundles to Improve Health Care Quality. Cambridge, Massachusetts: Institute for Healthcare Improvements; 2012.

31. Arora KS, Shields LE, Grobman WA, D'Alton ME, Lappen JR, Mercer BM. Triggers, bundles, protocols, and checklists--what every maternal care provider needs to know. Am J Obstet

For peer review only

Gynecol. 2016;214(4):444-51. Epub 2015/10/20. doi: 10.1016/j.ajog.2015.10.011. PubMed PMID: 26478105.

Supporting information

S1 Table S1. Interventions and criteria pondering results

S2. Supplemental Annex 1. Search strategies

S3. Supplemental Annex 2. Flowcharts for literature review

For peer review only

Table 1. List of interventions proposed

Potential interventions
1. ß-blockers at least at initial dose, plan increase to maximum tolerable dose during outpatient care.
2. Angiotensin converting enzyme inhibitors (ACE)-inhibitor or Angiotensin II antagonists at least at initial dose, plan increase to its maximum tolerable dose during outpatient care.
3. Mineralocorticoid antagonists at initial dose, plan increase to maximum tolerable dose if ejection fraction is ≦ 35% during outpatient care.
4. Ivabradine at least at initial dose and plan increase to maximum tolerable dose during outpatient care, if heart rate >70 bpm despite ß-blockers at maximum tolerable dose and being in sinus rhythm.
5. Angiotensin receptor–neprilysin inhibitor (ARNI) at least at initial dose, plan increase to maximum tolerable dose during outpatient care.
6. Diuretic dose should be adjusted based on imaging, laboratory, and functional class examinations.
7. Implant a resynchronizer in case of complete blockage of the left branch with a QRS >0.15 sec, in sinus rhythm, receiving optimal medical treatment and persisting in heart failure Class III-IV.
8. Antipneucoccal and influenza vaccines.
9. Measure ferritin and correct if low (<100 ng/mL or 100 to 300 ng/mL if transferrin saturation is <20%).
10. Measure urea, creatinine, calculate GFR and study ionogram (sodium, potassium and chlorine) to establish prognosis and therapy feasibility.
11. Perform cardiac ultrasound and communicate results to treating physician to establish therapeutic action, adjust medication and improve risk stratification.
12. Perform pulmonary ultrasound and communicate result to treating physician to establish therapeutic action, adjust medication and improve the patient's risk stratification.
13. Fit pulmonary artery pressure monitoring device for treating physician to improve monitoring and adjust medication.

14. Measure BNP or NT-proBNP and report to treating physician to establish therapeutic action, adjust medication and improve the patient's risk stratification.

15. Monitor blood pressure and try to reach values between 130/80 - 110/70, prioritizing use of ß-blockers and ACE-inhibitor or angiotensin receptor neprilysin inhibitor

16. Instruct patient to weigh daily and consult or increase the diuretic dose if increase is ≧ 2kg in 3 days.

17. Indicate patient to ingest a maximum of 2grs of sodium per day and inform which foods are rich in sodium.

18. If patient is a smoker, recommend smoking cessation and an action plan to achieve it.

19. Advise patient to lose weight if obese or overweight and not in advanced stages of CHF.

20. Advise patient to perform aerobic exercise at least 4 times/week for 40 min up to 80% of predicted HR for their age.

21. Within 1 week of discharge, specialist nurse should call patient to follow-up on adherence to dietary hygiene measures, medication and its adverse effects, weight gain and monitoring by the cardiologist.

22. Schedule an appointment for a consultation with the specialist within one week of discharge.

23. Patient should be evaluated by a psychologist/psychiatrist to screen for depression and initiate treatment accordingly.

24. Reassure patient that there are no restrictions on sexual activity. If phosphodiesterase inhibitors are used, the patient should be informed about contraindications and precautions.

25. Identify the cause of decompensation of IC.

26. If patient has obstructive sleep apnoea, treatment with continuous positive airway pressure should be initiated.

Table 2. Proposed Bundle

Interventions	
Pre discharge medications	Beta Blockers – reach optimal dose
	Angiotensin-converting enzyme inhibitors (ACE-inhibitors) or angiotensin receptor/neprilysin inhibitor (ARNI) reach optimal dose
	Anti-mineralocorticoids – reach optimal dose

For peer review only

	Furosemide – reach optimal dose
Continuum of care after discharge	Follow up appointment with the specialist seven days after discharge
	Daily weight monitoring under same conditions
Lifestyle habits	Low sodium diet – intake of <2grs of sodium per day
	Tobacco cessation counseling
Predischarge tests	Renal function and ionogram evaluation
	Blood pressure control (pre-discharge)
	Echocardiogram during hospitalization
	Determination of the decompensating cause

Title of Figure 1:

Flowchart of Delphi process

For peer review only

For peer review only - http://bmjopen.bmj.com/site/about/guidelines.xhtml

Figure 1. Flowchart of Delphi process

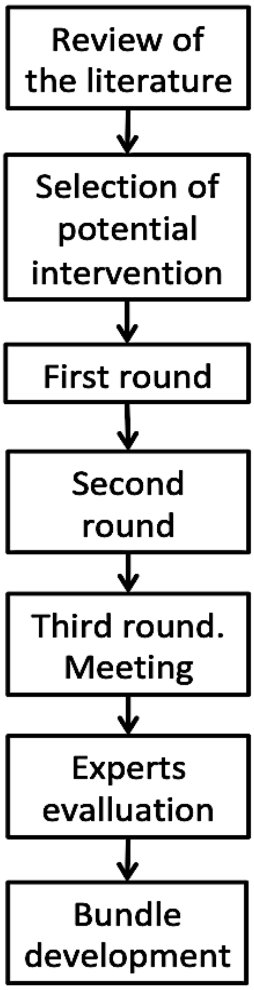

Supplemental Table 1. Interventions and criteria pondering results

	1. Priority	2. Intended effects	3. Unintended effects	4. Balance	5. Quality of evidence on effects	6.Values and preferences	7. Resource required	8. Quality of evidence on resources	9. Cost-effectiveness	10. Equity	11. Acceptability	12. Feasibility	13. Measurability		
Weighting factor	1.00	1.00	0.89	0.89	0.89	0.78	0.78	0.78	0.89	0.89	0.78	0.89	0.78	Score	
Recommendation															
ACE-inhibitors	9.00	9.00	2.67	7.11	8.00	6.22	7.00	6.22	8.00	7.11	7.00	8.00	7.00	**96.00**	7.38
Lab	9.00	9.00	1.78	7.11	7.11	6.22	7.00	5.44	8.00	7.56	7.00	8.00	7.00	**95.67**	7.36
Blood Pressure	8.00	8.00	2.67	7.11	7.11	6.22	7.00	6.22	8.00	7.11	6.61	8.00	6.61	**92.33**	7.10
Dairy weight	8.50	8.50	1.78	8.00	6.22	6.22	6.22	5.44	7.11	7.56	6.22	7.11	6.22	**90.56**	6.97
Beta block	9.00	9.00	2.67	7.11	8.00	6.22	6.22	2.72	7.11	7.11	7.00	7.56	7.00	**90.39**	6.95
Sodium	8.50	8.00	1.78	7.11	6.22	5.44	7.00	5.44	7.56	7.56	6.22	7.56	5.44	**89.28**	6.87
Follow-up	9.00	9.00	1.78	7.56	6.22	6.22	4.67	5.44	7.11	7.11	6.22	7.11	6.22	**89.11**	6.85
Smoking cesation	9.00	9.00	2.22	7.11	7.11	4.67	5.44	5.44	7.11	7.11	6.22	6.22	5.44	**86.67**	6.67
Anti mineralocorticoids	8.00	8.00	4.44	6.22	8.00	5.44	6.22	6.22	7.11	7.11	6.22	7.11	6.22	**86.44**	6.65
Diuretics	9.00	8.00	4.44	6.67	5.33	5.44	6.61	5.44	7.11	6.67	6.22	8.00	7.00	**86.06**	6.62
Sacubitril	8.00	9.00	2.67	7.11	8.00	6.22	4.67	6.22	6.22	5.33	5.44	6.22	7.00	**85.78**	6.60
Echocardiography	8.00	8.00	0.89	7.11	6.22	6.22	3.89	4.67	7.11	6.22	6.22	6.22	6.61	**84.61**	6.51
Descompensation Cause	9.00	8.50	4.44	7.11	6.22	6.22	5.06	5.44	7.11	6.67	6.22	6.22	6.22	**84.56**	6.50
Telephone tracking	8.00	8.00	1.78	7.11	6.22	6.22	4.28	5.44	6.67	6.22	5.44	5.33	6.22	**82.39**	6.34
Flu vaccine	7.00	7.00	2.22	6.67	5.33	5.44	5.44	4.67	7.11	7.11	6.22	7.11	6.22	**82.11**	6.32
Weightloss	7.00	7.00	2.67	6.22	5.33	4.67	5.83	4.67	6.22	5.78	5.44	6.22	5.44	**76.17**	5.86
Exercicse	7.00	7.00	7.11	7.11	6.22	5.44	6.22	3.89	6.22	7.11	5.44	6.22	5.44	**75.22**	5.79
Sexual activity	7.00	7.00	4.00	6.22	4.44	5.44	6.22	4.67	6.22	6.22	4.67	6.22	4.67	**74.00**	5.69
ProBNP	7.00	7.00	1.78	6.22	6.22	5.44	3.89	5.44	4.44	3.56	3.89	4.44	6.22	**71.00**	5.46
Lung echography	6.00	6.00	1.78	6.67	5.33	5.06	3.89	4.67	4.89	4.44	5.06	5.33	6.22	**70.78**	5.44
Psychologist	6.00	7.00	3.56	6.22	5.33	4.67	4.67	4.67	6.22	5.33	4.67	4.44	4.67	**69.33**	5.33
Ferritin	7.00	3.00	2.67	6.22	6.22	4.67	3.89	3.89	5.78	4.44	4.67	5.33	5.83	**67.28**	5.18
Ivabrav	6.00	6.00	2.67	6.22	6.22	4.67	5.44	4.67	5.33	4.44	4.28	6.22	5.83	**67.22**	5.17
Apnea	7.00	7.00	3.56	6.22	5.78	4.67	2.33	4.67	5.33	3.56	4.67	4.00	5.44	**66.11**	5.09
Resynchronizer	6.00	4.00	3.56	6.22	6.67	4.67	1.56	5.44	5.33	2.67	4.67	4.00	6.22	**62.89**	4.84
Wireless HF monitor	3.00	6.00	2.67	4.44	5.33	2.33	0.78	4.28	4.44	1.78	2.72	1.78	5.44	**48.67**	3.74

Supplemental Annex 1. Search strategies

Search	Query	Items found
#17	Search **(#15 AND #16)**	1046
#16	Search **((Systematic Review[sb] OR Systematic Review[tiab] OR Meta-Analysis[pt] OR Meta-Analys*[tiab] OR "Cochrane Database Syst Rev"[ta] OR Metaanalysis[tiab] OR Metanalysis[tiab] OR Sysrev_Methods[sb] OR (MEDLINE[tiab] AND Cochrane[tiab]) OR Guideline[pt] OR Practice Guideline[pt] OR Guideline*[ti] OR Guide Line*[tiab] OR Consensus[tiab] OR Recommendation*[ti] OR Randomized Controlled Trial[pt] OR Random*[ti] OR Controlled Trial*[tiab] OR Control Trial*[tiab] OR Technology Assessment, Biomedical[Mesh] OR Technology Assessment[tiab] OR Technology Appraisal[tiab] OR HTA[tiab] OR Overview[ti] OR (Review[ti] AND Literature[ti])))**	1277580
#15	Search **(#8 AND #14)**	5959
#14	Search **(#9 OR #10 OR #11 OR #12 OR #13)**	37520
#13	Search **Re-Hospitaliz*[tiab]**	1206
#12	Search **Rehospitaliz*[tiab]**	5251
#11	Search **Readmitted[tiab]**	6511
#10	Search **Readmission*[tiab]**	24703
#9	Search **Patient Readmission[Mesh]**	15507
#8	Search **(#1 OR #2 OR #3 OR #4 OR #5 OR #6 OR #7)**	204191
#7	Search **Heart Failure[tiab]**	163364
#6	Search **Myocardial Decompensat*[tiab]**	33
#5	Search **Myocardial Failure[tiab]**	757
#4	Search **Cardiac Decompensat*[tiab]**	1156
#3	Search **Heart Decompensat*[tiab]**	122
#2	Search **Cardiac Failure[tiab]**	11760
#1	Search **Heart Failure[MeSH]**	115775

For peer review only

No.	Query	Results
#19	#17 AND #18	**80**
#18	(((('systematic review':ti,ab OR 'meta analysis (topic)':pt OR 'meta analysis':ti,ab OR cochrane:jt OR metaanalysis:ti,ab OR metanalysis.ti,ab. OR (medline:ab AND cochrane:ab) OR practice) AND guideline:pt OR guideline*:ti OR 'guide lines':ti,ab OR consensus:ti,ab OR recommendation*:ti OR randomized) AND controlled AND trial:pt OR random*:ti OR controlled) AND trial*:ti,ab OR 'control trial':ti,ab OR biomedical) AND technology AND 'assessment'/exp OR 'technology assessment':ti,ab OR 'technology appraisal':ti,ab OR hta:ti,ab OR overview:ti OR (review:ti AND literature:ti)	**194196**

No.	Query	Results
#17	#8 AND #16	**16307**
#16	#9 OR #10 OR #11 OR #12 OR #13 OR #14 OR #15	**75899**
#15	're hospitaliz*':ti,ab	**3444**
#14	're hospitalis*':ti,ab	**486**
#13	rehospitalis*:ti,ab	**1208**
#12	rehospitaliz*:ti,ab	**8201**
#11	readmitted:ti,ab	**13183**
#10	readmission*:ti,ab	**45071**
#9	'hospital readmission'/exp	**56483**
#8	#1 OR #2 OR #3 OR #4 OR #5 OR #6 OR #7	**536876**
#7	'heart failure':ti,ab	**259617**
#6	(myocardial NEAR/1 decompensat*):ti,ab	**57**
#5	'myocardial failure':ti,ab	**934**
#4	(cardiac NEAR/1 decompensat*):ti,ab	**1917**
#3	(heart NEAR/1 decompensat*):ti,ab	**6759**
#2	'cardiac failure':ti,ab	**16881**
#1	'heart failure'/exp	**491308**

CINAHL (EBSCO) 26-09-2019

#	Query	Results
S22	S20 AND S21	610
S21	(MH "Systematic Review") OR (MH "Cochrane Library") OR TI Meta-Analysis OR AB Meta-Analysis OR TI Metaanalysis OR AB Metaanalysis OR (AB Medline AND AB Cochrane) OR TI Guideline* OR AB Guideline* OR TI "Guide Lines" OR AB "Guide Lines" OR TI Consensus OR TI Recommendation* OR TI Random* OR AB Random* OR TI "Controlled Trial" OR AB "Controlled Trial" OR TI "Technology Assessment" OR AB "Technology Assessment" OR TI "Technology Appraisal" OR AB "Technology Appraisal" OR TI HTA OR AB HTA OR TI Overview OR (TI Review AND TI Literature)	547,021
S20	S13 AND S19	2,297
S19	S14 OR S15 OR S16 OR S17 OR S18	13,923
S18	TI Re-Hospitali* OR AB Re-Hospitali*	504
S17	TI Rehospitali* OR AB Rehospitali*	2,196
S16	TI Readmitted OR AB Readmitted	2,020

For peer review only - http://bmjopen.bmj.com/site/about/guidelines.xhtml

S15	TI Readmission* OR AB Readmission*	12,455
S14	(MH "Readmission")	11,566
S13	S1 OR S2 OR S3 OR S4 OR S5 OR S6 OR S7 OR S8 OR S9 OR S10 OR S11 OR S12	35,130
S12	TI "Heart Failure" OR AB "Heart Failure"	44,414
S11	TI "Myocardial Decompensated" OR AB "Myocardial Decompensated"	173
S10	TI "Myocardial Decompensation" OR AB "Myocardial Decompensation"	7
S9	TI "Myocardial Failure" OR AB "Myocardial Failure"	44
S8	TI "Cardiac Decompensated" OR AB "Cardiac Decompensated"	386
S7	TI "Cardiac Decompensated" OR AB "Cardiac Decompensated"	0
S6	TI "Cardiac Decompensation" OR AB "Cardiac Decompensation"	98
S5	TI "Heart Decompensated" OR AB "Heart Decompensated"	1,597
S4	TI "Heart Decompensated" OR AB "Heart Decompensated"	0
S3	TI "Heart Decompensation" OR AB "Heart Decompensation"	8
S2	TI "Cardiac Failure" OR AB "Cardiac Failure"	1,227
S1	(MH "Heart Failure+")	36,791

Global Health (OVID) 26-09-2019

☐	# ▲	Searches	Results
☐	1	Cardiac Failure.ti,ab.	1058
☐	2	Heart Decompensat*.ti,ab.	1
☐	3	Cardiac Decompensat*.ti,ab.	38
☐	4	Myocardial Failure.ti,ab.	61
☐	5	Myocardial Decompensat*.ti,ab.	0
☐	6	Heart Failure.ti,ab.	9089
☐	7	or/1-6	10064
☐	8	Readmission*.ti,ab.	2050
☐	9	Readmitted.ti,ab.	1113
☐	10	Rehospitaliz*.ti,ab.	310
☐	11	Re-Hospitaliz*.ti,ab.	121
☐	12	or/8-11	3203
☐	13	7 and 8	158

Estrategias Heart Failure Bundles

For peer review only

PubMed 24-09-2019

	Query	Items found
#14	**(#13 NOT Branch[tiab])**	319
#13	**(#4 AND #12)**	2109
#12	**(#5 OR #6 OR #7 OR #8 OR #9 OR #10 OR #11)**	204129
#11	**Heart Failure[tiab]**	163313
#10	**Myocardial Decompensat*[tiab]**	33
#9	**Myocardial Failure[tiab]**	757
#8	**Cardiac Decompensat*[tiab]**	1155
#7	**Heart Decompensat*[tiab]**	122
#6	**Cardiac Failure[tiab]**	11758
#5	**Heart Failure[MeSH]**	115710
#4	**(#1 OR #2 OR #3)**	62341
#3	**Bundles[tiab]**	27534
#2	**Bundle[tiab]**	38958
#1	**Patient Care Bundles[MeSH]**	662

EMBase (Elsevier) 24-09-2019

No.	Query	Results
#14	#13 NOT branch:ti,ab	**1009**
#13	#4 AND #12	**5474**
#12	#5 OR #6 OR #7 OR #8 OR #9 OR #10 OR #11	**536741**
#11	(myocardial NEAR/1 decompensat*):ti,ab	**57**
#10	'myocardial failure':ti,ab	**934**
#9	(cardiac NEAR/1 decompensat*):ti,ab	**1917**
#8	(heart NEAR/1 decompensat*):ti,ab	**6757**
#7	'cardiac failure':ti,ab	**16879**
#6	'heart failure':ti,ab	**259558**
#5	'heart failure'/exp	**491184**
#4	#1 OR #2 OR #3	**78092**
#3	bundles:ti,ab	**31971**
#2	bundle:ti,ab	**51066**
#1	'care bundle'/exp	**1150**

CINAHL (EBSCO) 24-09-2019

#	Query	Results
S13	S12 NOT (TI Branch OR AB Branch)	74
S12	S3 AND S11	548
S11	S4 OR S5 OR S6 OR S7 OR S8 OR S9 OR S10	34,961
S10	TI "Heart Failure" OR AB "Heart Failure"	44,368
S9	TI "Myocardial Decompensation" OR AB "Myocardial Decompensation"	7
S8	TI "Myocardial Failure" OR AB "Myocardial Failure"	44
S7	TI "Cardiac Decompensation" OR AB "Cardiac Decompensation"	98
S6	(TI "Heart Decompensation" OR AB "Heart Decompensation") OR (TI "Heart Decompensated" OR "Heart Decompensated")	8
S5	TI "Cardiac Failure" OR AB "Cardiac Failure"	1,227
S4	(MH "Heart Failure+")	36,787
S3	S1 OR S2	6,601
S2	TI Bundles OR AB Bundles	6,463
S1	TI Bundle OR AB Bundle	6,572

Cochrane Library (Wiley) 24-09-2019

ID Search Hits
#1 MeSH descriptor: [Patient Care Bundles] explode all trees 21
#2 Bundle:ti,ab,kw 1731
#3 Bundles:ti,ab,kw 354
#4 #1 OR #2 OR #3 1968
#5 MeSH descriptor: [Heart Failure] explode all trees 8355
#6 (Cardiac NEAR/1 Failure):ti,ab,kw 993
#7 (Heart NEAR/1 Decompesat*):ti,ab,kw 1
#8 (Cardiac NEAR/1 Decompesat*):ti,ab,kw 0
#9 (Myocardial NEAR/1 Failure):ti,ab,kw 118
#10 (Myocardial NEAR/1 Decompensat*):ti,ab,kw 1
#11 (Heart NEAR/1 Failure):ti,ab,kw 27302
#12 #5 OR #6 OR #7 OR #8 OR #9 OR #10 OR #11 27757
#13 #4 AND #12 338
#14 #13 NOT Branch:ti,ab,kw 32

Global Health (OVID) 26-09-2019

	# ▲	Searches	Results
☐	1	Bundle.ti,ab.	1822
☐	2	Bundles.ti,ab.	1467
☐	3	1 or 2	3054
☐	4	Cardiac Failure.ti,ab.	1058

For peer review only

☐	5	Heart Decompensat*.ti,ab.	1
☐	6	Cardiac Decompensat*.ti,ab.	38
☐	7	Myocardial Failure.ti,ab.	61
☐	8	Myocardial Decompensat*.ti,ab.	0
☐	9	Heart Failure.ti,ab.	9089
☐	10	or/4-9	10064
☐	11	3 and 10	66
☐	12	11 not Branch.ti,ab.	17

For peer review only

Supplemental Annex 2. Flowcharts

Flow chart for literature review of HF interventions

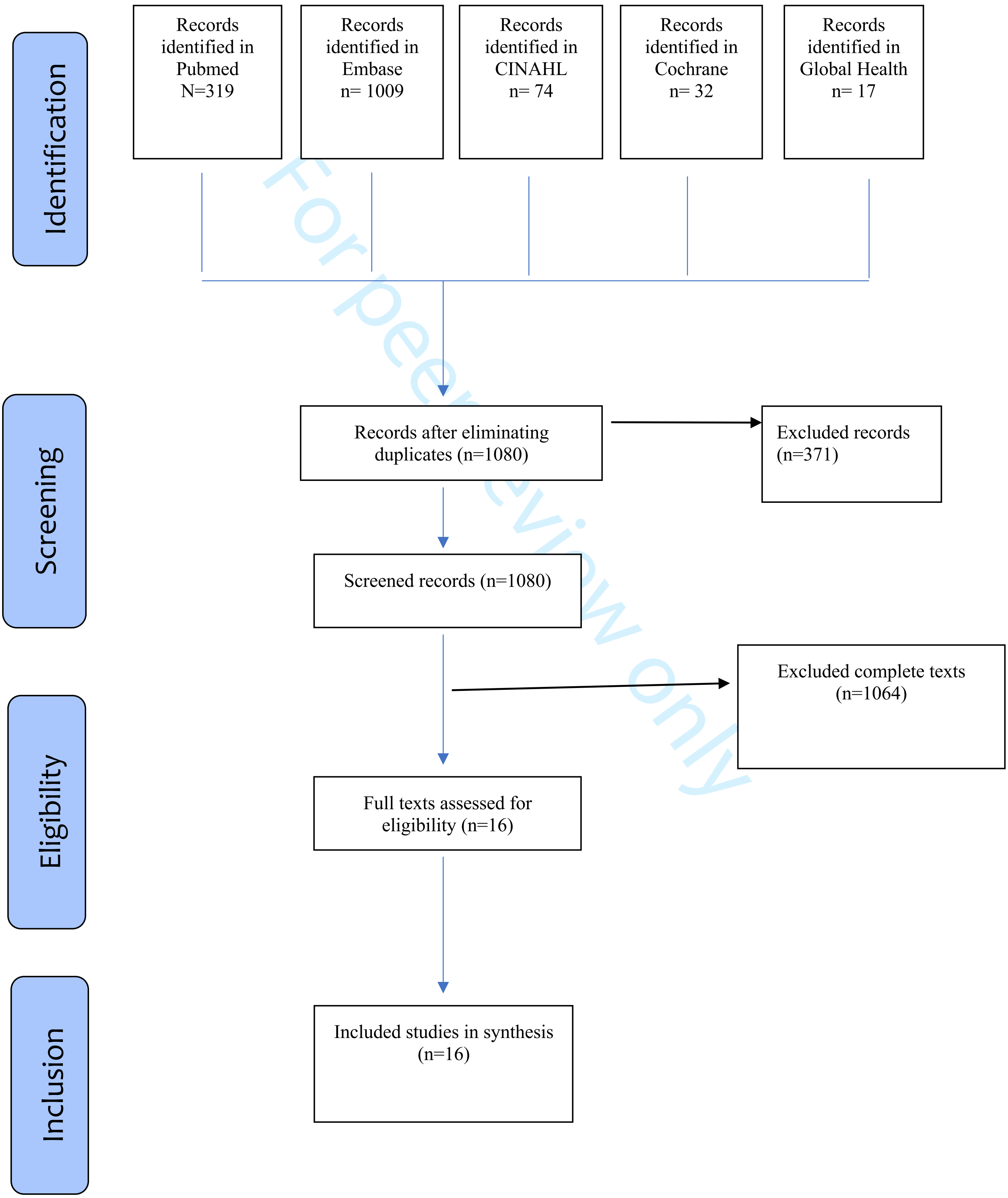

For peer review only

Flow chart for literature review of Readmission

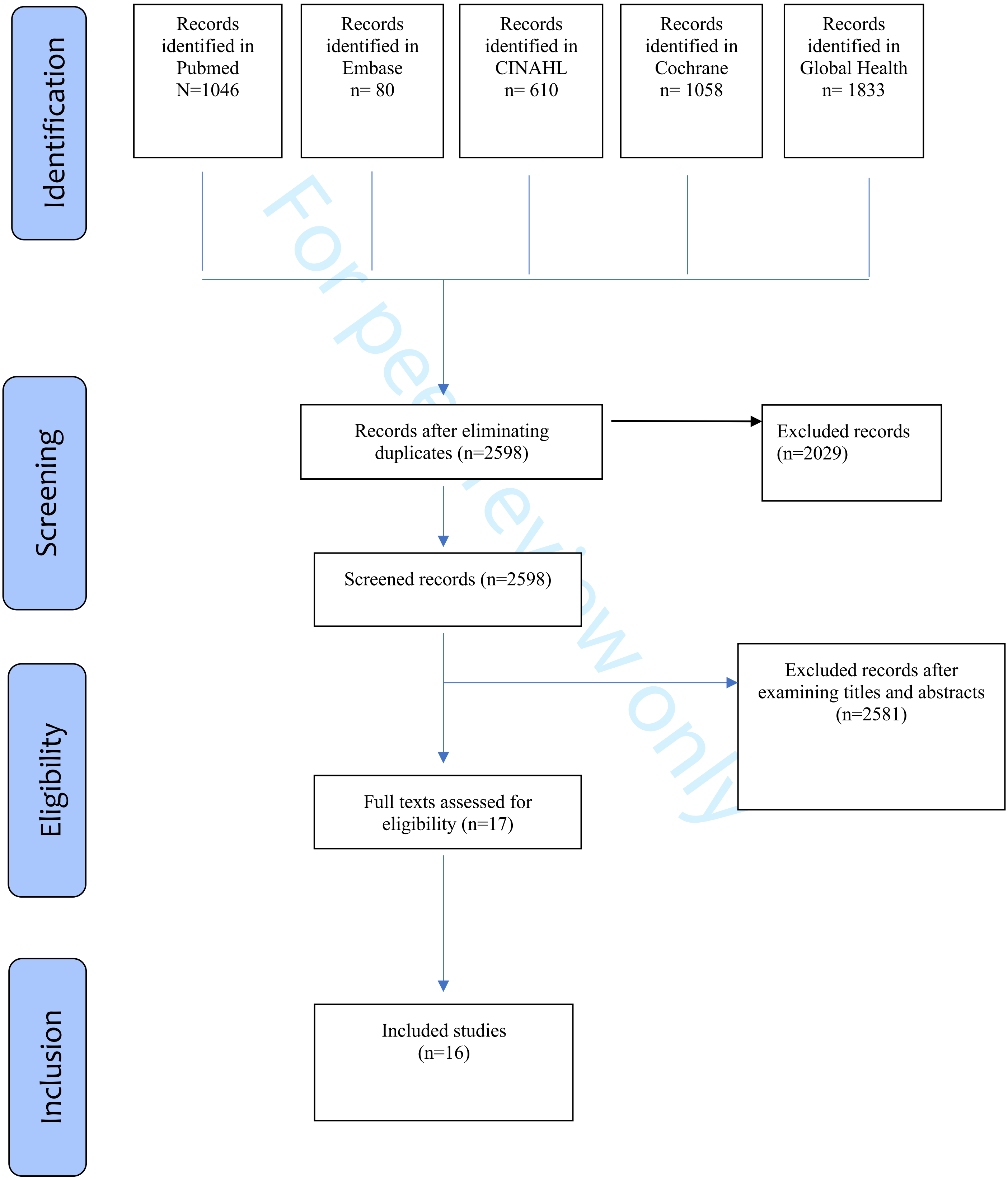

For peer review only

Care Bundle to Reduce Readmission in Patients with Heart Failure: A Modified Delphi Consensus Panel in Argentina

Journal:	*BMJ Open*
Manuscript ID	bmjopen-2020-040028.R2
Article Type:	Original research
Date Submitted by the Author:	24-Nov-2020
Complete List of Authors:	Roberti, Javier; Universidad Nacional de Entre Rios; Institute for Clinical Effectiveness and Health Policy<br>Vita, Tomas; Institute for Clinical Effectiveness and Health Policy<br>Piastrella, Jimena; Novartis Argentina SA<br>Porley, Carlos; Novartis Argentina SA<br>Pereyra, Lisandro; Novartis Argentina SA<br>Diez, Mirta; Instituto Cardiovascular de Buenos Aires<br>Renedo, Florencia; Fundacion Favaloro Hospital Universitario<br>Fairman, Enrique; Clinica Bazterrica<br>Fernandez, Alberto; Sanatorio Modelo Quilmes<br>Thierer, Jorge; CEMIC<br>Garcia Elorrio, Ezequiel; Institute for Clinical Effectiveness and Health Policy
**Primary Subject Heading**:	Cardiovascular medicine
Secondary Subject Heading:	Cardiovascular medicine
Keywords:	Adult cardiology < CARDIOLOGY, Heart failure < CARDIOLOGY, Quality in health care < HEALTH SERVICES ADMINISTRATION & MANAGEMENT, Protocols & guidelines < HEALTH SERVICES ADMINISTRATION & MANAGEMENT

SCHOLARONE™ Manuscripts

BMJ

I, the Submitting Author has the right to grant and does grant on behalf of all authors of the Work (as defined in the below author licence), an exclusive licence and/or a non-exclusive licence for contributions from authors who are: i) UK Crown employees; ii) where BMJ has agreed a CC-BY licence shall apply, and/or iii) in accordance with the terms applicable for US Federal Government officers or employees acting as part of their official duties; on a worldwide, perpetual, irrevocable, royalty-free basis to BMJ Publishing Group Ltd ("BMJ") its licensees and where the relevant Journal is co-owned by BMJ to the co-owners of the Journal, to publish the Work in this journal and any other BMJ products and to exploit all rights, as set out in our licence.

The Submitting Author accepts and understands that any supply made under these terms is made by BMJ to the Submitting Author unless you are acting as an employee on behalf of your employer or a postgraduate student of an affiliated institution which is paying any applicable article publishing charge ("APC") for Open Access articles. Where the Submitting Author wishes to make the Work available on an Open Access basis (and intends to pay the relevant APC), the terms of reuse of such Open Access shall be governed by a Creative Commons licence – details of these licences and which Creative Commons licence will apply to this Work are set out in our licence referred to above.

Other than as permitted in any relevant BMJ Author's Self Archiving Policies, I confirm this Work has not been accepted for publication elsewhere, is not being considered for publication elsewhere and does not duplicate material already published. I confirm all authors consent to publication of this Work and authorise the granting of this licence.

For peer review only

Care Bundle to Reduce Readmission in Patients with Heart Failure: A Modified Delphi Consensus Panel in Argentina

Javier Roberti[1,2*], Tomás Vita[1], Jimena Piastrella[3], Carlos Porley[3], Lisandro Pereyra[3] Mirta Diez[4], Florencia Renedo[5], Enrique Fairman[6], Alberto Fernández[7], Jorge Thierer[8], Ezequiel García Elorrio[1]

1. Institute for Clinical Effectiveness and Health Policy (IECS), Buenos Aires, Argentina
2. National Scientific and Technical Research Council (CONICET)- Argentina
3. Novartis Argentina, Buenos Aires, Argentina
4. Instituto Cardiológico Buenos Aires – ICBA, Buenos Aires, Argentina
5. Fundación Favaloro, Buenos Aires, Argentina
6. Clínica Bazterrica, Buenos Aires, Argentina
7. Clínica Modelo Quilmes, Quilmes, Argentina
8. CEMIC, Buenos Aires, Argentina

***Corresponding Author**

Javier Roberti, Email: jroberti@iecs.org.ar

For peer review only

Abstract

Objectives. The aim of this study was to develop consensus among Argentine cardiologists on a care bundle to reduce readmissions of patients with heart failure (HF).

Setting: Hospitals and cardiology clinics in Argentina that provide in-hospital care for patients with HF.

Participants: Twenty-four cardiology experts participated in the two online rounds and 18 (75%) of them participated in the third-round meeting.

Methods: This study used a mixed method design; it was conducted between August 2019 and January 2020. The development of a care bundle (a set of evidence-based interventions applied to improve clinical outcomes) involved three phases: a) a literature review to define the list of interventions to be evaluated; b) a modified Delphi panel to select interventions for the bundle; c) definition of the HF care bundle. Also, the process included three rounds of scoring.

Results: Twenty-six interventions were evaluated. The interventions in the final bundle covered four categories: medication, continuum of care, lifestyle habits, predischarge tests. These were: Medication: Beta-blockers, angiotensin receptor neprilysin inhibitors or ACE-inhibitors, furosemide and anti-mineralocorticoids; Continuum of care: follow-up appointment, daily weight monitoring; Lifestyle habits: smoking cessation counseling and

For peer review only

low-sodium diet; Pre-discharge tests: renal function, ionogram, blood pressure control, echocardiogram and determination of decompensating cause.

Conclusion: Following a systematic mixed method approach, we have developed a care bundle of interventions that could decrease readmission of patients with HF. The application of this bundle could contribute to scale evidence-based interventions.

For peer review only

Article summary

Strengths and limitations of this study

- Potential interventions were chosen through a systematic review
- Cardiologist experts participated in a transparent consensus process
- As in most consensuses, participants could have misinterpreted statements
- Potential bias from cardiologists as only specialty involved in process.

Funding statement: This work was supported by Novartis Argentina SA, grant number NCC CLCZ696BAR05R

Introduction

Despite several medical advances to treat heart failure (HF), mortality and hospital readmission have not changed significantly.[1] The adherence to treatment and other

related responsibilities demanded by the health system place a significant burden on patients and their caregivers.[2,3] Moreover, a high percentage of patients with HF are not receiving an adequate treatment despite the increased use of both evidence-based therapies and performance measures.[4-6] In this context, the use of a care bundle with additional strategies such as quality improvement collaboratives (QICs) to scale-up its use could contribute to the optimization of the treatment of patients with HF.[7,8]

A care bundle is defined as a set of evidence-based interventions, called elements, that should be applied together in every eligible patient to enhance the reliability of care and to improve clinical outcomes.[9,10] The completion of the interventions of a bundle should be measured as all-or-nothing; when all components were performed collectively and reliably, they improved patient outcomes.[11] Therefore, a care bundle approach for HF should focus on providing evidence-based clinical practice, engaging patients and careers as active partners, and creating processes to ensure a quality handoff from hospital care.

Hospitalizations in HF are the main trigger for treatment interventions; however, financers' lack of awareness of the clinical burden and the low urgency to intervene in these patients compared to other cardiovascular diseases represent significant challenges for implementation.[12,13] In fact, HF guidelines recommend initiating and uptitrating disease-modifying therapies during hospitalization.[14] As the preliminary phase of a future QIC, the aim of this study was to develop consensus among Argentine cardiologists on a care bundle to reduce hospital readmissions of patients with HF.

For peer review only

Methods

This study used a mixed method design and was conducted between August 2019 and January 2020. The approach used to develop a care bundle involved three phases: a) a literature review to define the list of interventions that would be evaluated; b) a modified Delphi panel to select interventions for the bundle; finally, c) development of the final HF care bundle. The process included seven steps, with three rounds of scoring. See Figure 1 for an illustration of the study design.

(Figure 1)

Phase 1.

Step 1. Explorative review of the literature

In preparation of the Delphi questionnaire to be distributed, a review of the literature was performed, using a pragmatic exploratory approach. We searched in PubMed, LILACS, EMBASE, The Cochrane Library and Google Scholar for relevant literature. No HF care bundle was identified in the literature; although a review of gray literature showed isolated experiences shared over the internet. We included articles describing interventions for HF, with special attention to those specified in the Guidelines of the American College of Cardiology (ACC), the American Heart Association (AHA), the European Society of Cardiology (ESC) and the Argentine Cardiology Society.[7,8,15] Supplemental annex 1 shows search strategies used; supplemental annex 2 shows the PRISMA flowchart of selection process.

(Table 1)

For peer review only - http://bmjopen.bmj.com/site/about/guidelines.xhtml

Step 2. Developing the list of interventions

The target patient for the interventions was an individual with signs and symptoms of HF and an ejection fraction lower than 40% who had been admitted to hospital and was expected to be discharged during the following 48 hours. Based on the literature, we proposed a list of 26 interventions to be assessed by experts. To guide the selection of interventions during the consensus process, we used the criteria proposed by GRADE from *Evidence to Decision* framework validated and adopted by WHO (project EtD / DECIDE) and others from the care package literature to help panels of experts use evidence in a structured and transparent way to inform decisions in the context of healthcare recommendations.[10] Based on the literature, we selected a list of 26 interventions to be assessed by experts. Table 1 shows the 26 interventions. The potential interventions were presented to panelists as short statements with references to supporting literature. For each statement, panelists were asked to rate interventions according to 13 criteria or parameters using a nine-point Likert scale ranging from 1 (extremely inappropriate / insignificant) to 9 (extremely appropriate/ significant).[16] The 13 criteria were 1) priority, 2) unintended effects, 3) intended effects, 4) balance between unintended and intended effects, 5) quality of evidence on effects, 6) values and preferences, 7) resource required, 8) quality of evidence on resources, 9) cost-effectiveness, 10) equity, 11) acceptability, 12) feasibility, and 13) measurability. The EtD framework ensures that panels consider important criteria, contributes to structure discussions and to identify the reasons for disagreements, based on the balance of health benefits and harms, human rights and sociocultural acceptability, health equity, equality and non-discrimination, societal implications, financial and economic considerations,

For peer review only

feasibility and health system considerations and the meta-criterion quality of evidence.[17,18]

Phase 2. Consensus on a care bundle.

Step 1. Selection of experts' panel

We used a modified RAND-UCLA Delphi method in which experts used their professional judgment alongside the best available evidence to identify areas where consensus could be reached for the topic under consideration.[16] A total of 26 experts in chronic HF were selected from different clinical contexts in Argentina, mainly from the high-density area of Buenos Aires. All participants were specialists in cardiology, with proven experience in treating patients with HF and in scientific research on HF, were currently practicing in hospitals which had a cardiology fellowship, at least 80 beds, and with 3,000 admissions or more per year. These physicians were known to the team for their involvement in HF treatment and research and some of them were recommended by the first selected participants. The 26 potential participants were sent a recruitment letter and information sheet via e-mail; follow-up telephone calls were made to confirm that the material had been received. Of the total, 24 (92%) professionals accepted the invitation. Panelists were given a small *honorarium* for their time and effort.

Step 2. Interventions rating

Permanent disagreement was defined as at least six panelists rating the indication in the 1-3 region, and at least six panelists rating it in the 7-9 region after three rounds of scoring. Each intervention obtained 13 scores, one for each parameter or criterion.[16] Then, this score was multiplied by the weighting factor obtained in the rating of the parameters and

For peer review only

mean was calculated for each intervention. Interventions were then sorted according to this global score.

Step 3. First round online survey

For the first round, the experts received a link for the online questionnaire (surveymonkey.com, San Mateo, California, USA). Twenty-six interventions in 13 criteria were evaluated for disagreement following the classic RAND definition of at least six panelists rating the parameter in the 1-3 region and at least six panelists rating it in the 7-9 region.[16] Security sockets layer (SSL) encryption was used to protect data while being transmitted by ensuring secure connections between participants and the server. An email was issued to each participant requesting them to open the survey tool, answer the questions and press the submit button. All participants had the contact details for the research team that could be contacted in case of any doubts regarding the materials provided. Participants had 10 days to complete the survey; a reminder email was sent to non-responders.

Step 4. Second online survey round

In round two, all interventions and criteria that had not reach an agreement in the first round were included for a new vote. Participants were shown the whole group's ratings as frequency data on a rating scale for each item, alongside their own ratings for each statement in round one. They could revise their own original rating considering the group ratings if they so wished. Participants had 10 days to complete round two; a reminder was sent to non-responders.

For peer review only

Step 5. Consensus meeting

All participants of the online survey were invited via email and telephone to a three-hour in-person consensus meeting. The main objectives of the meeting were to discuss the indications that have been rated with disagreement and inconsistencies in appropriateness ratings. Panelists were encouraged to refer to the literature. Of the total of participants who responded the online rounds, 18 (75%) took part in the meeting. During the session, results of the second online survey were presented and discussed, followed by an anonymous onsite rating of those items that had showed disagreement on previous rounds. In addition, the 13 quality criteria were anonymously rated using a 1-9 Likert scale to obtain a factor for final evaluation of each intervention. The panel mean score was calculated for each statement and classified into three categories: 1 – 3.5 (potentially inappropriate); 3.6 – 6.4 (uncertain), and 6.5 – 9 (potentially appropriate). The moderator was a physician, not a specialist in the area to avoid bringing their bias to the discussion, had led the literature review and was familiar with the material.

Step 6. Development of HF care bundle with expert committee

We invited five of the participants of previous rounds with extensive expertise to join an expert committee to discuss the results of the survey and rate the bundle. The results of the process were presented in this meeting. The expert committee selected those interventions with the highest scores for the final evaluation and no disagreements. Based on these results, a final bundle of interventions was developed.

For peer review only

Ethical aspects and role of funding source

Written informed consent was obtained from all participants included in the study, and all procedures were conducted according to the Declaration of Helsinki. The founder of the study did not intervene in any stage of the consensus process. This study was evaluated and approved by an independent ethics committee, Independent Ethics Committee for Clinical Pharmacology Trials Prof Luis M. Zieher, Buenos Aires, Argentina, protocol number 0042/19v.05 on 17 October 2019

Patient and Public Involvement

The research question addresses a most significant issue for patients and was informed by a review of the literature on patients' journey when admitted for heart failure care. Only physicians with a vast clinical experience participated in the consensus process; these participants brought patients' concerns and perspectives into the process. The results of this study are disseminated via open access publication. This study is complemented by a qualitative research on patients' perspectives, reported separately.

Results

Twenty-four experts participated in the two online rounds and 18 (75%) of them participated in the third-round meeting. Eight (33.3%) were female; 16 (66.7%) participants worked in the private sector, 2 (8.3%) in the public sector and 6 (25%) in both sectors. Sixteen (66.7%) participants worked in the metropolitan area of Buenos Aires, while the rest, 8 (33.3%) participants, worked in other provinces of Argentina. The median of professional experience was 26.5 years (range, 9 – 40 years).

For peer review only

Twenty-six interventions were evaluated, and each intervention obtained a global score. After the three rounds, consensus was reached for all interventions except for one intervention under one criterion (unintended effects of telephone tracking of patients). The mean global scores of 13 interventions were above the cutoff score of 6.5 and seven of these reached a median score of 7.1 (Supplemental table 1). Of the 13 final interventions, 5 (38%) were related to medications, 2 (15%) were diagnostic tests, and 2 (15%) were habit recommendations (“hyposodic diet” and “daily weight monitoring”). The two most valuable criteria ranked by experts were the priority and the desirable effects of each intervention. “Administration of ACE-inhibitors”, the “evaluation of renal function”, “performance of an ionogram” and “blood pressure control” were considered the most important interventions. The intervention “use of beta-blockers” was ranked in the fifth position while the “use of anti-mineralocorticoids”, “furosemide” and “angiotensin receptor neprilysin inhibitor (ARNI)” were in the ninth, the tenth and the eleventh positions, respectively. The use of ARNI positioned better on undesirable effects than the administration of furosemide and anti-mineralocorticoids. “The administration of ARNI” and “the use of ACE-inhibitors” obtained the same scores in six criteria; mainly related to scientific evidence evaluation; of note, under the criteria evaluating equity, ACE-inhibitors obtained a higher score. “Smoking cessation” and “blood pressure control” were ranked higher than the use of medication because, unlike medication, the first two had no potential undesirable effects according to participants.

“Daily weight monitoring” and “a follow-up appointment within a week of discharge” were ranked in the fourth and the seventh positions, respectively. “Echocardiography during

For peer review only

hospitalization” and “determining the cause of decompensation” were ranked above the established cut-off point. “Measurement of biomarker pro-BNP and BNP” ranked below the cut-off point. “Physical exercise” and “cardiac resynchronization therapy” did not rank highly for the overall goal of reducing hospital readmission.

Proposed Bundle

The proposed bundle was based on four categories of interventions: medication, continuum of care, lifestyle habits, predischarge tests (Table 2). Beta-blockers, ARNI or ACE-inhibitors, furosemide and anti-mineralocorticoids were included as interventions related to pharmacologic treatment. ARNI or ACE-inhibitors can be used interchangeably. The election would depend on clinician judge and patient choice. Follow-up appointment with a specialist within a week of discharge and daily weight monitoring were also included as interventions that reduce 30-day hospital readmission under “continuum of care” category, while smoking cessation counseling and low sodium diet were interventions classified as lifestyle habits. The interventions included under pre discharge tests were “renal function evaluation”, “performance of ionogram”, “blood pressure control”, “performance of an echocardiogram during hospitalization” and “determination of the decompensating cause”.

(Table 2)

Discussion

Following a validated and systematic consensus method, we created a care bundle of interventions that could contribute to the management of HF and prevent readmission within the 30 days after discharge. The reduction of readmissions of patients with HF

For peer review only

represents a challenge because several factors such as a lack of a targeted therapy for patients with preserved ejection fraction, the natural history of HF, the need for daily management to avoid decompensations, and problems related to access to care, health literacy, and other socioeconomic factors.[19] HF is a complex syndrome and the result of congestion and/or poor perfusion, leading to negative effects on multiple organ systems. In fact, patients with HF have multiple comorbidities, a heavy medication burden, and experience symptoms that may be attributed to several etiologies.[19]

Several studies have evaluated single interventions to improve outcomes in patients with HF. Although the ESC, ACC and HF Argentinean guidelines have established recommendations; critical interventions have not been specified.[7,8,15]. For participants, the priority and desirable effects were the two most important criteria to evaluate each intervention. Probably, these two criteria are what cardiologists in the local context refer to when assessing any treatment in time-constrained settings. We have defined priorities in a time constrained setting as it is usually the case with clinical work. As all the potential interventions had been recommended on HF guidelines, no significant differences in global scores were observed among those above the cut-off point. Indeed, when the results were shown to the experts, they all agreed on the significance of the interventions that were finally included in the bundle.

Almost half of the interventions were related to medication, which, with robust evidence supporting its efficacy, is widely accepted as the backbone of HF treatment. It has been shown that interventions to improve adherence to medications for HF significantly reduce the risk for both hospitalization and death.[4] Interestingly, the use of beta-blockers was only fifth on the interventions ranking although these drugs have been confirmed to

For peer review only - http://bmjopen.bmj.com/site/about/guidelines.xhtml

reduce mortality, readmission and improve symptomatology.[20-23] It is possible that the use of beta-blockers was not chosen as the most important intervention in our study as many patients have HF in very advanced stages and require inotropes during hospitalization, while beta-blockers are administered during outpatient care. Clinical trials of ARNI showed a significant reduction in mortality and readmission rates when compared to ACE inhibitors.[24,25] The administration of ACE-inhibitors was pondered as the most important intervention and, at a granular level, the difference between ARNI and ACE-inhibitors was based mainly on costs and access. The high cost of the drug in an economically constrained setting explains the gap between evidence and real-world practice. Due to new evidence, the final recommendations in the bundle allow the physician to decide between ARNI or ACE-inhibitors.[24,25]

Interestingly, many of the interventions were not strictly clinical practices such as daily weight monitoring or a follow-up appointment, which reflects the importance of an integrated care and the complex setting in which professionals and the health system interact to achieve the best quality outcomes. Moreover, in a follow-up appointment, the patient could be provided with information to prevent re-admission. Importantly, laboratory examinations such as tests of renal function and the ionogram obtained a high score and were valued as the second most important intervention. Probably related to this, of the five medications included in the bundle, four could negatively affect renal function. Also, in the Argentinian health system, BNP test is expensive and not covered by most health insurances, which could explain the fact that this test was not included in the final list of elements.

For peer review only - http://bmjopen.bmj.com/site/about/guidelines.xhtml

In pioneering care bundles, two components were essential to their success; participating clinicians agreed that there was sufficient medical evidence supporting each individual element.[26-29] Second, the list of elements in the bundle should not be extensive; the goal of the bundle approach is to develop a short list of interventions already recommended and accepted in guidelines. This allows improvement rather than a debate on validity of the elements. Moreover, it is essential that elements have the consensus of local clinicians.[30] On the other hand, checklists and protocols serve to augment memory and limit the chance of human error, improving communication, standardizing responses, and reducing unnecessary clinical variation.[31]

Our study has limitations that should be acknowledged. Participants could have misinterpreted the original statements describing the potential interventions and the same applies to criteria definitions. We tried to control this factor by providing participants with information and discussing definitions during the meeting. An important limitation is related to the method itself; our results reflect the opinion of selected specialists with a vast experience. Cardiologists see most of HF patients and once the bundle applicability is assessed, it could be disseminated to other contexts that include other specialties. The interventions were not tested in community or primary care, but this bundle is intended to be used during patient hospitalization, before discharge. Also, we disclosed the funding by a pharmaceutical company and the limited participation of professionals affiliated to this company, which could be interpreted as a limitation. We have made every effort to maintain the transparency of the process in every stage.

In conclusion, following a mixed method approach, we developed a care bundle of interventions that could decrease readmission of patients with HF. Our next step will be

For peer review only

to study the implementation of this bundle in several sites. The application of this bundle could be used to scale evidence-based interventions under the framework of Quality Improvement Learning Collaborative.

Acknowledgements

We thank the participation of the following health professionals: José Luis Barisani (Clínica Adventista), Roberto Colque (Sanatorio Allende), Marcelo Crespo (Sanatorio Los Arcos), Clara Huerta (Hospital Córdoba), Juan Pablo Escalante (Instituto Cardiovascular Rosario), David Flores (Hospital Nacional de Clínicas), Jimena Gambarte (Hospital Alemán), Daniela García Brasca (Hospital Italiano de Córdoba), Alfredo Larraburu (Sanatorio Los Arcos), Adrián Lescano (Centro Gallego), Javier Marino (ICBA), Lidia Lobo Márquez (Instituto de Cardiología), Cristian Mastantuono (Hospital Pirovano), Felipe Martínez (Universidad Nacional de Córdoba), Stella Pereiro (Hospital Churruca), Eduardo Perna (Instituto de Cardiología de Corrientes Juana Francisca Cabral), José Santucci (Hospital Austral), Julieta Soricetti (Hospital Durand), Ezequiel Zaidel (Sanatorio Güemes).

A. **Contributorship statement:** Javier Roberti: Methodology, investigation, analysis, writing. Tomas Vita: investigation, analysis, writing. Jimena Piastrella, Carlos Porley, and Lisandro Pereyra: investigation, interpretation, review; Mirta Diez, Florencia Renedo, Enrique Fairman, Alberto Fernández, Jorge Thierer: investigation, interpretation, review; Ezequiel García Elorrio: concept, methodology, analysis, interpretation, review.

B. **Competing interests:** Jimena Piastrella, Carlos Porley, and Lisandro Pereyra are employees of Novartis Argentina SA, funder of this study. Javier Roberti, Tomas Vita,

Florencia Renedo, Enrique Fairman, Alberto Fernandez, Jorge Thierer and Ezequiel Garcia Elorrio have no competing interest to declare.

C. **Funding**: This study was supported by Novartis Argentina SA (grant number NCC CLCZ696BAR05R).

D. **Data sharing statement**: Supplementary data such as search strategies, flowcharts and table of scores are available in supplementary materials and upon request.

References

1. Bytyci I, Bajraktari G. Mortality in heart failure patients. Anatol J Cardiol. 2015;15(1):63-8. Epub 2015/01/01. doi: 10.5152/akd.2014.5731.

2. Nordfonn OK, Morken IM, Bru LE, Husebo AML. Patients' experience with heart failure treatment and self-care-A qualitative study exploring the burden of treatment. J Clin Nurs. 2019;28(9-10):1782-93. Epub 2019/01/23. doi: 10.1111/jocn.14799.

3. Evangelista LS, Shinnick MA. What do we know about adherence and self-care? J Cardiovasc Nurs. 2008;23(3):250-7. Epub 2008/04/26. doi: 10.1097/01.JCN.0000317428.98844.4d.

4. Ruppar TM, Cooper PS, Mehr DR, Delgado JM, Dunbar-Jacob JM. Medication Adherence Interventions Improve Heart Failure Mortality and Readmission Rates: Systematic Review and Meta-Analysis of Controlled Trials. J Am Heart Assoc. 2016;5(6). Epub 2016/06/19. doi: 10.1161/JAHA.115.002606.

5. Fonarow GC, Abraham WT, Albert NM, Gattis Stough W, Gheorghiade M, Greenberg BH, et al. Influence of a performance-improvement initiative on quality of care for patients hospitalized

For peer review only - http://bmjopen.bmj.com/site/about/guidelines.xhtml

with heart failure: results of the Organized Program to Initiate Lifesaving Treatment in Hospitalized Patients With Heart Failure (OPTIMIZE-HF). Arch Intern Med. 2007;167(14):1493-502. doi: 10.1001/archinte.167.14.1493.

6. Panella M, Marchisio S, Demarchi ML, Manzoli L, Di Stanislao F. Reduced in-hospital mortality for heart failure with clinical pathways: the results of a cluster randomised controlled trial. Qual Saf Health Care. 2009;18(5):369-73. doi: 10.1136/qshc.2008.026559.

7. Ponikowski P, Voors AA, Anker SD, Bueno H, Cleland JG, Coats AJ, et al. 2016 ESC Guidelines for the diagnosis and treatment of acute and chronic heart failure: The Task Force for the diagnosis and treatment of acute and chronic heart failure of the European Society of Cardiology (ESC) Developed with the special contribution of the Heart Failure Association (HFA) of the ESC. Eur Heart J. 2016;37(27):2129-200. doi: 10.1093/eurheartj/ehw128.

8. Yancy CW, Jessup M, Bozkurt B, Butler J, Casey DE, Jr., Colvin MM, et al. 2017 ACC/AHA/HFSA Focused Update of the 2013 ACCF/AHA Guideline for the Management of Heart Failure: A Report of the American College of Cardiology/American Heart Association Task Force on Clinical Practice Guidelines and the Heart Failure Society of America. J Am Coll Cardiol. 2017;70(6):776-803. doi: 10.1016/j.jacc.2017.04.025.

9. Lavallee JF, Gray TA, Dumville J, Russell W, Cullum N. The effects of care bundles on patient outcomes: a systematic review and meta-analysis. Implement Sci. 2017;12(1):142. doi: 10.1186/s13012-017-0670-0.

10. Resar R, Griffin F, Haraden C, Nolan T. Using Care Bundles to Improve Health Care Quality. Cambridge, Massachusetts: Institute for Healthcare Improvement;, 2012.

For peer review only

11. Agency for Healthcare Research and Quality. Eliminating CLABSI: A National Patient Safety Imperative. Final Report. Rockville, MD: 2013. Available online at https://www.ahrq.gov/hai/cusp/clabsi-final/index.html. Accessed 10 December 2019.

12. Arora S, Patel P, Lahewala S, Patel N, Patel NJ, Thakore K, et al. Etiologies, Trends, and Predictors of 30-Day Readmission in Patients With Heart Failure. Am J Cardiol. 2017;119(5):760-9. Epub 2017/01/23. doi: 10.1016/j.amjcard.2016.11.022.

13. Wan TTH, Terry A, Cobb E, McKee B, Tregerman R, Barbaro SDS. Strategies to Modify the Risk of Heart Failure Readmission: A Systematic Review and Meta-Analysis. Health Serv Res Manag Epidemiol. 2017;4:2333392817701050. Epub 2017/05/04. doi: 10.1177/2333392817701050.

14. Seferovic PM, Ponikowski P, Anker SD, Bauersachs J, Chioncel O, Cleland JGF, et al. Clinical practice update on heart failure 2019: pharmacotherapy, procedures, devices and patient management. An expert consensus meeting report of the Heart Failure Association of the European Society of Cardiology. Eur J Heart Fail. 2019;21(10):1169-86. Epub 2019/05/28. doi: 10.1002/ejhf.1531.

15. Sociedad Argentina de Cardiología. Consenso de insuficiencia cardíaca crónica. Revista Argentina de Cardiología. 2016; 84(S3):3-50.

16. Fitch K, Bernstein SJ, Aguilar DM, Burnand B, LaCalle JR, Lazaro P, et al. The RAND/UCLA Appropriateness Method User's Manual. Santa Monica, CA: RAND Corporation, 2001. https://www.rand.org/pubs/monograph_reports/MR1269.html. Also available in print form

For peer review only

17. Moberg J, Oxman AD, Rosenbaum S, Schunemann HJ, Guyatt G, Flottorp S, et al. The GRADE Evidence to Decision (EtD) framework for health system and public health decisions. Health Res Policy Syst. 2018;16(1):45. Epub 2018/05/31. doi: 10.1186/s12961-018-0320-2.

18. Alonso-Coello P, Schunemann HJ, Moberg J, Brignardello-Petersen R, Akl EA, Davoli M, et al. [GRADE Evidence to Decision (EtD) frameworks: a systematic and transparent approach to making well informed healthcare choices. 1: Introduction]. Gac Sanit. 2018;32(2):166 e1- e10. Epub 2017/08/22. doi: 10.1016/j.gaceta.2017.02.010.

19. Goldgrab D, Balakumaran K, Kim MJ, Tabtabai SR. Updates in heart failure 30-day readmission prevention. Heart Fail Rev. 2019;24(2):177-87. Epub 2018/11/30. doi: 10.1007/s10741-018-9754-4.

20. Hjalmarson A, Goldstein S, Fagerberg B, Wedel H, Waagstein F, Kjekshus J, et al. Effects of controlled-release metoprolol on total mortality, hospitalizations, and well-being in patients with heart failure: the Metoprolol CR/XL Randomized Intervention Trial in congestive heart failure (MERIT-HF). MERIT-HF Study Group. JAMA. 2000;283(10):1295-302. doi: 10.1001/jama.283.10.1295.

21. Packer M, Coats AJ, Fowler MB, Katus HA, Krum H, Mohacsi P, et al. Effect of carvedilol on survival in severe chronic heart failure. N Engl J Med. 2001;344(22):1651-8. doi: 10.1056/NEJM200105313442201.

22. Packer M, Bristow MR, Cohn JN, Colucci WS, Fowler MB, Gilbert EM, et al. The effect of carvedilol on morbidity and mortality in patients with chronic heart failure. U.S. Carvedilol Heart Failure Study Group. N Engl J Med. 1996;334(21):1349-55. doi: 10.1056/NEJM199605233342101.

For peer review only

23. Effect of metoprolol CR/XL in chronic heart failure: Metoprolol CR/XL Randomised Intervention Trial in Congestive Heart Failure (MERIT-HF). Lancet. 1999;353(9169):2001-7.

24. McMurray JJ, Packer M, Desai AS, Gong J, Lefkowitz MP, Rizkala AR, et al. Angiotensin-neprilysin inhibition versus enalapril in heart failure. N Engl J Med. 2014;371(11):993-1004. doi: 10.1056/NEJMoa1409077.

25. Wachter R, Senni M, Belohlavek J, Straburzynska-Migaj E, Witte KK, Kobalava Z, et al. Initiation of sacubitril/valsartan in haemodynamically stabilised heart failure patients in hospital or early after discharge: primary results of the randomised TRANSITION study. Eur J Heart Fail. 2019;21(8):998-1007. doi: 10.1002/ejhf.1498.

26. Resar R, Pronovost P, Haraden C, Simmonds T, Rainey T, Nolan T. Using a Bundle Approach to Improve Ventilator Care Processes and Reduce Ventilator-Associated Pneumonia. The Joint Commission Journal on Quality and Patient Safety. 2005;31(5):243-8. doi: 10.1016/s1553-7250(05)31031-2.

27. Burger CD, Resar RK. "Ventilator bundle" approach to prevention of ventilator-associated pneumonia. Mayo Clin Proc. 2006;81(6):849-50. Epub 2006/06/15. doi: 10.4065/81.6.849.

28. Bird D, Zambuto A, O'Donnell C, Silva J, Korn C, Burke R, et al. Adherence to ventilator-associated pneumonia bundle and incidence of ventilator-associated pneumonia in the surgical intensive care unit. Arch Surg. 2010;145(5):465-70. Epub 2010/05/19. doi: 10.1001/archsurg.2010.69.

For peer review only

For peer review only - http://bmjopen.bmj.com/site/about/guidelines.xhtml

29. Furuya EY, Dick A, Perencevich EN, Pogorzelska M, Goldmann D, Stone PW. Central line bundle implementation in US intensive care units and impact on bloodstream infections. PLoS One. 2011;6(1):e15452. Epub 2011/01/27. doi: 10.1371/journal.pone.0015452.

30. Resar R, Griffin F, Haraden C, Nolan T. Using Care Bundles to Improve Health Care Quality. Cambridge, Massachusetts: Institute for Healthcare Improvements; 2012.

31. Arora KS, Shields LE, Grobman WA, D'Alton ME, Lappen JR, Mercer BM. Triggers, bundles, protocols, and checklists--what every maternal care provider needs to know. Am J Obstet Gynecol. 2016;214(4):444-51. Epub 2015/10/20. doi: 10.1016/j.ajog.2015.10.011. PubMed PMID: 26478105.

Supporting information

S1 Table S1. Interventions and criteria pondering results

S2. Supplemental Annex 1. Search strategies

S3. Supplemental Annex 2. Flowcharts for literature review

Table 1. List of interventions proposed

Potential interventions
1. ß-blockers at least at initial dose, plan increase to maximum tolerable dose during outpatient care.
2. Angiotensin converting enzyme inhibitors (ACE)-inhibitor or Angiotensin II antagonists at least at initial dose, plan increase to its maximum tolerable dose during outpatient care.
3. Mineralocorticoid antagonists at initial dose, plan increase to maximum tolerable dose if ejection fraction is ≦ 35% during outpatient care.
4. Ivabradine at least at initial dose and plan increase to maximum tolerable dose during outpatient care, if heart rate >70 bpm despite ß-blockers at maximum tolerable dose and being in sinus rhythm.
5. Angiotensin receptor–neprilysin inhibitor (ARNI) at least at initial dose, plan increase to maximum tolerable dose during outpatient care.
6. Diuretic dose should be adjusted based on imaging, laboratory, and functional class examinations.

For peer review only

7. Implant a resynchronizer in case of complete blockage of the left branch with a QRS >0.15 sec, in sinus rhythm, receiving optimal medical treatment and persisting in heart failure Class III-IV.

8. Antipneucoccal and influenza vaccines.

9. Measure ferritin and correct if low (<100 ng/mL or 100 to 300 ng/mL if transferrin saturation is <20%).

10. Measure urea, creatinine, calculate GFR and study ionogram (sodium, potassium and chlorine) to establish prognosis and therapy feasibility.

11. Perform cardiac ultrasound and communicate results to treating physician to establish therapeutic action, adjust medication and improve risk stratification.

12. Perform pulmonary ultrasound and communicate result to treating physician to establish therapeutic action, adjust medication and improve the patient's risk stratification.

13. Fit pulmonary artery pressure monitoring device for treating physician to improve monitoring and adjust medication.

14. Measure BNP or NT-proBNP and report to treating physician to establish therapeutic action, adjust medication and improve the patient's risk stratification.

15. Monitor blood pressure and try to reach values between 130/80 - 110/70, prioritizing use of ß-blockers and ACE-inhibitor or angiotensin receptor neprilysin inhibitor

16. Instruct patient to weigh daily and consult or increase the diuretic dose if increase is ≧ 2kg in 3 days.

17. Indicate patient to ingest a maximum of 2grs of sodium per day and inform which foods are rich in sodium.

18. If patient is a smoker, recommend smoking cessation and an action plan to achieve it.

19. Advise patient to lose weight if obese or overweight and not in advanced stages of CHF.

20. Advise patient to perform aerobic exercise at least 4 times/week for 40 min up to 80% of predicted HR for their age.

21. Within 1 week of discharge, specialist nurse should call patient to follow-up on adherence to dietary hygiene measures, medication and its adverse effects, weight gain and monitoring by the cardiologist.

22. Schedule an appointment for a consultation with the specialist within one week of discharge.

23. Patient should be evaluated by a psychologist/psychiatrist to screen for depression and initiate treatment accordingly.

24. Reassure patient that there are no restrictions on sexual activity. If phosphodiesterase inhibitors are used, the patient should be informed about contraindications and precautions.

25. Identify the cause of decompensation of IC.

26. If patient has obstructive sleep apnoea, treatment with continuous positive airway pressure should be initiated.

For peer review only

Table 2. Proposed Bundle

Interventions	
Pre discharge medications	Beta Blockers – reach optimal dose
	Angiotensin-converting enzyme inhibitors (ACE-inhibitors) or

	angiotensin receptor/neprilysin inhibitor (ARNI) reach optimal dose
	Anti-mineralocorticoids – reach optimal dose
	Furosemide – reach optimal dose
Continuum of care after discharge	Follow up appointment with the specialist seven days after discharge
	Daily weight monitoring under same conditions
Lifestyle habits	Low sodium diet – intake of <2grs of sodium per day
	Tobacco cessation counseling
Predischarge tests	Renal function and ionogram evaluation
	Blood pressure control (pre-discharge)
	Echocardiogram during hospitalization
	Determination of the decompensating cause

Title of Figure 1:

Flowchart of Delphi process

For peer review only

Figure 1. Flowchart of Delphi process

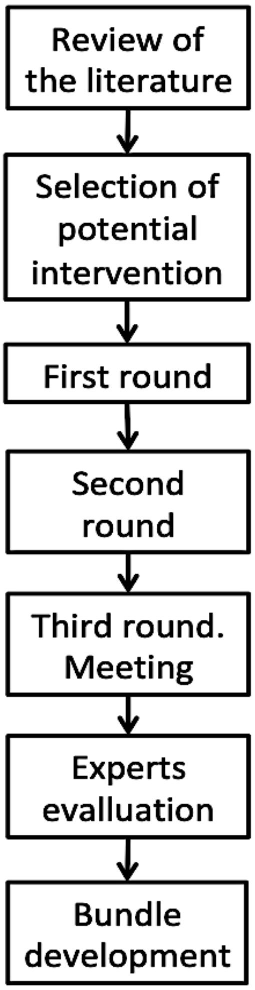

For peer review only

Supplemental Table 1. Interventions and criteria pondering results

	1. Priority	2. Intended effects	3. Unintended effects	4. Balance	5. Quality of evidence on effects	6.Values and preferences	7. Resource required	8. Quality of evidence on resources	9. Cost-effectiveness	10. Equity	11. Acceptability	12. Feasibility	13. Measurability	Score	
Weighting factor	1.00	1.00	0.89	0.89	0.89	0.78	0.78	0.78	0.89	0.89	0.78	0.89	0.78		
Recommendation															
ACE-inhibitors	9.00	9.00	2.67	7.11	8.00	6.22	7.00	6.22	8.00	7.11	7.00	8.00	7.00	**96.00**	7.38
Lab	9.00	9.00	1.78	7.11	7.11	6.22	7.00	5.44	8.00	7.56	7.00	8.00	7.00	**95.67**	7.36
Blood Pressure	8.00	8.00	2.67	7.11	7.11	6.22	7.00	6.22	8.00	7.11	6.61	8.00	6.61	**92.33**	7.10
Dairy weight	8.50	8.50	1.78	8.00	6.22	6.22	6.22	5.44	7.11	7.56	6.22	7.11	6.22	**90.56**	6.97
Beta block	9.00	9.00	2.67	7.11	8.00	6.22	6.22	2.72	7.11	7.11	7.00	7.56	7.00	**90.39**	6.95
Sodium	8.50	8.00	1.78	7.11	6.22	5.44	7.00	5.44	7.56	7.56	6.22	7.56	5.44	**89.28**	6.87
Follow-up	9.00	9.00	1.78	7.56	6.22	6.22	4.67	5.44	7.11	7.11	6.22	7.11	6.22	**89.11**	6.85
Smoking cesation	9.00	9.00	2.22	7.11	7.11	4.67	5.44	5.44	7.11	7.11	6.22	6.22	5.44	**86.67**	6.67
Anti mineralocorticoids	8.00	8.00	4.44	6.22	8.00	5.44	6.22	6.22	7.11	7.11	6.22	7.11	6.22	**86.44**	6.65
Diuretics	9.00	8.00	4.44	6.67	5.33	5.44	6.61	5.44	7.11	6.67	6.22	8.00	7.00	**86.06**	6.62
Sacubitril	8.00	9.00	2.67	7.11	8.00	6.22	4.67	6.22	6.22	5.33	5.44	6.22	7.00	**85.78**	6.60
Echocardiography	8.00	8.00	0.89	7.11	6.22	6.22	3.89	4.67	7.11	6.22	6.22	6.22	6.61	**84.61**	6.51
Descompensation Cause	9.00	8.50	4.44	7.11	6.22	6.22	5.06	5.44	7.11	6.67	6.22	6.22	6.22	**84.56**	6.50
Telephone tracking	8.00	8.00	1.78	7.11	6.22	6.22	4.28	5.44	6.67	6.22	5.44	5.33	6.22	**82.39**	6.34
Flu vaccine	7.00	7.00	2.22	6.67	5.33	5.44	5.44	4.67	7.11	7.11	6.22	7.11	6.22	**82.11**	6.32
Weightloss	7.00	7.00	2.67	6.22	5.33	4.67	5.83	4.67	6.22	5.78	5.44	6.22	5.44	**76.17**	5.86
Exercicse	7.00	7.00	7.11	7.11	6.22	5.44	6.22	3.89	6.22	7.11	5.44	6.22	5.44	**75.22**	5.79
Sexual activity	7.00	7.00	4.00	6.22	4.44	5.44	6.22	4.67	6.22	6.22	4.67	6.22	4.67	**74.00**	5.69
ProBNP	7.00	7.00	1.78	6.22	6.22	5.44	3.89	5.44	4.44	3.56	3.89	4.44	6.22	**71.00**	5.46
Lung echography	6.00	6.00	1.78	6.67	5.33	5.06	3.89	4.67	4.89	4.44	5.06	5.33	6.22	**70.78**	5.44
Psychologist	6.00	7.00	3.56	6.22	5.33	4.67	4.67	4.67	6.22	5.33	4.67	4.44	4.67	**69.33**	5.33
Ferritin	7.00	3.00	2.67	6.22	6.22	4.67	3.89	3.89	5.78	4.44	4.67	5.33	5.83	**67.28**	5.18
Ivabrav	6.00	6.00	2.67	6.22	6.22	4.67	5.44	4.67	5.33	4.44	4.28	6.22	5.83	**67.22**	5.17
Apnea	7.00	7.00	3.56	6.22	5.78	4.67	2.33	4.67	5.33	3.56	4.67	4.00	5.44	**66.11**	5.09
Resynchronizer	6.00	4.00	3.56	6.22	6.67	4.67	1.56	5.44	5.33	2.67	4.67	4.00	6.22	**62.89**	4.84
Wireless HF monitor	3.00	6.00	2.67	4.44	5.33	2.33	0.78	4.28	4.44	1.78	2.72	1.78	5.44	**48.67**	3.74

Supplemental Annex 1. Search strategies

Search	Query	Items found
#17	Search **(#15 AND #16)**	1046
#16	Search **((Systematic Review[sb] OR Systematic Review[tiab] OR Meta-Analysis[pt] OR Meta-Analys*[tiab] OR "Cochrane Database Syst Rev"[ta] OR Metaanalysis[tiab] OR Metanalysis[tiab] OR Sysrev_Methods[sb] OR (MEDLINE[tiab] AND Cochrane[tiab]) OR Guideline[pt] OR Practice Guideline[pt] OR Guideline*[ti] OR Guide Line*[tiab] OR Consensus[tiab] OR Recommendation*[ti] OR Randomized Controlled Trial[pt] OR Random*[ti] OR Controlled Trial*[tiab] OR Control Trial*[tiab] OR Technology Assessment, Biomedical[Mesh] OR Technology Assessment[tiab] OR Technology Appraisal[tiab] OR HTA[tiab] OR Overview[ti] OR (Review[ti] AND Literature[ti])))**	1277580
#15	Search **(#8 AND #14)**	5959
#14	Search **(#9 OR #10 OR #11 OR #12 OR #13)**	37520
#13	Search **Re-Hospitaliz*[tiab]**	1206
#12	Search **Rehospitaliz*[tiab]**	5251
#11	Search **Readmitted[tiab]**	6511
#10	Search **Readmission*[tiab]**	24703
#9	Search **Patient Readmission[Mesh]**	15507
#8	Search **(#1 OR #2 OR #3 OR #4 OR #5 OR #6 OR #7)**	204191
#7	Search **Heart Failure[tiab]**	163364
#6	Search **Myocardial Decompensat*[tiab]**	33
#5	Search **Myocardial Failure[tiab]**	757
#4	Search **Cardiac Decompensat*[tiab]**	1156
#3	Search **Heart Decompensat*[tiab]**	122
#2	Search **Cardiac Failure[tiab]**	11760
#1	Search **Heart Failure[MeSH]**	115775

For peer review only

No.	Query	Results
#19	#17 AND #18	**80**
#18	(((('systematic review':ti,ab OR 'meta analysis (topic)':pt OR 'meta analysis':ti,ab OR cochrane:jt OR metaanalysis:ti,ab OR metanalysis.ti,ab. OR (medline:ab AND cochrane:ab) OR practice) AND guideline:pt OR guideline*:ti OR 'guide lines':ti,ab OR consensus:ti,ab OR recommendation*:ti OR randomized) AND controlled AND trial:pt OR random*:ti OR controlled) AND trial*:ti,ab OR 'control trial':ti,ab OR biomedical) AND technology AND 'assessment'/exp OR 'technology assessment':ti,ab OR 'technology appraisal':ti,ab OR hta:ti,ab OR overview:ti OR (review:ti AND literature:ti)	**194196**

No.	Query	Results
#17	#8 AND #16	**16307**
#16	#9 OR #10 OR #11 OR #12 OR #13 OR #14 OR #15	**75899**
#15	're hospitaliz*':ti,ab	**3444**
#14	're hospitalis*':ti,ab	**486**
#13	rehospitalis*:ti,ab	**1208**
#12	rehospitaliz*:ti,ab	**8201**
#11	readmitted:ti,ab	**13183**
#10	readmission*:ti,ab	**45071**
#9	'hospital readmission'/exp	**56483**
#8	#1 OR #2 OR #3 OR #4 OR #5 OR #6 OR #7	**536876**
#7	'heart failure':ti,ab	**259617**
#6	(myocardial NEAR/1 decompensat*):ti,ab	**57**
#5	'myocardial failure':ti,ab	**934**
#4	(cardiac NEAR/1 decompensat*):ti,ab	**1917**
#3	(heart NEAR/1 decompensat*):ti,ab	**6759**
#2	'cardiac failure':ti,ab	**16881**
#1	'heart failure'/exp	**491308**

CINAHL (EBSCO) 26-09-2019

#	Query	Results
S22	S20 AND S21	610
S21	(MH "Systematic Review") OR (MH "Cochrane Library") OR TI Meta-Analysis OR AB Meta-Analysis OR TI Metaanalysis OR AB Metaanalysis OR (AB Medline AND AB Cochrane) OR TI Guideline* OR AB Guideline* OR TI “Guide Lines” OR AB “Guide Lines” OR TI Consensus OR TI Recommendation* OR TI Random* OR AB Random* OR TI “Controlled Trial” OR AB “Controlled Trial” OR TI “Technology Assessment” OR AB “Technology Assessment” OR TI “Technology Appraisal” OR AB “Technology Appraisal” OR TI HTA OR AB HTA OR TI Overview OR (TI Review AND TI Literature)	547,021
S20	S13 AND S19	2,297
S19	S14 OR S15 OR S16 OR S17 OR S18	13,923
S18	TI Re-Hospitali* OR AB Re-Hospitali*	504
S17	TI Rehospitali* OR AB Rehospitali*	2,196
S16	TI Readmitted OR AB Readmitted	2,020

review only

S15	TI Readmission* OR AB Readmission*	12,455
S14	(MH "Readmission")	11,566
S13	S1 OR S2 OR S3 OR S4 OR S5 OR S6 OR S7 OR S8 OR S9 OR S10 OR S11 OR S12	35,130
S12	TI "Heart Failure" OR AB "Heart Failure"	44,414
S11	TI "Myocardial Decompensated" OR AB "Myocardial Decompensated"	173
S10	TI "Myocardial Decompensation" OR AB "Myocardial Decompensation"	7
S9	TI "Myocardial Failure" OR AB "Myocardial Failure"	44
S8	TI "Cardiac Decompensated" OR AB "Cardiac Decompensated"	386
S7	TI "Cardiac Decompensated" OR AB "Cardiac Decompensated"	0
S6	TI "Cardiac Decompensation" OR AB "Cardiac Decompensation"	98
S5	TI "Heart Decompensated" OR AB "Heart Decompensated"	1,597
S4	TI "Heart Decompensated" OR AB "Heart Decompensated"	0
S3	TI "Heart Decompensation" OR AB "Heart Decompensation"	8
S2	TI "Cardiac Failure" OR AB "Cardiac Failure"	1,227
S1	(MH "Heart Failure+")	36,791

Global Health (OVID) 26-09-2019

☐	# ▲	Searches	Results
☐	1	Cardiac Failure.ti,ab.	1058
☐	2	Heart Decompensat*.ti,ab.	1
☐	3	Cardiac Decompensat*.ti,ab.	38
☐	4	Myocardial Failure.ti,ab.	61
☐	5	Myocardial Decompensat*.ti,ab.	0
☐	6	Heart Failure.ti,ab.	9089
☐	7	or/1-6	10064
☐	8	Readmission*.ti,ab.	2050
☐	9	Readmitted.ti,ab.	1113
☐	10	Rehospitaliz*.ti,ab.	310
☐	11	Re-Hospitaliz*.ti,ab.	121
☐	12	or/8-11	3203
☐	13	7 and 8	158

Estrategias Heart Failure Bundles

For peer review only

PubMed 24-09-2019

	Query	Items found
#14	**(#13 NOT Branch[tiab])**	319
#13	**(#4 AND #12)**	2109
#12	**(#5 OR #6 OR #7 OR #8 OR #9 OR #10 OR #11)**	204129
#11	**Heart Failure[tiab]**	163313
#10	**Myocardial Decompensat*[tiab]**	33
#9	**Myocardial Failure[tiab]**	757
#8	**Cardiac Decompensat*[tiab]**	1155
#7	**Heart Decompensat*[tiab]**	122
#6	**Cardiac Failure[tiab]**	11758
#5	**Heart Failure[MeSH]**	115710
#4	**(#1 OR #2 OR #3)**	62341
#3	**Bundles[tiab]**	27534
#2	**Bundle[tiab]**	38958
#1	**Patient Care Bundles[MeSH]**	662

EMBase (Elsevier) 24-09-2019

No.	Query	Results
#14	#13 NOT branch:ti,ab	**1009**
#13	#4 AND #12	**5474**
#12	#5 OR #6 OR #7 OR #8 OR #9 OR #10 OR #11	**536741**
#11	(myocardial NEAR/1 decompensat*):ti,ab	**57**
#10	'myocardial failure':ti,ab	**934**
#9	(cardiac NEAR/1 decompensat*):ti,ab	**1917**
#8	(heart NEAR/1 decompensat*):ti,ab	**6757**
#7	'cardiac failure':ti,ab	**16879**
#6	'heart failure':ti,ab	**259558**
#5	'heart failure'/exp	**491184**
#4	#1 OR #2 OR #3	**78092**
#3	bundles:ti,ab	**31971**
#2	bundle:ti,ab	**51066**
#1	'care bundle'/exp	**1150**

CINAHL (EBSCO) 24-09-2019

#	Query	Results
S13	S12 NOT (TI Branch OR AB Branch)	74
S12	S3 AND S11	548
S11	S4 OR S5 OR S6 OR S7 OR S8 OR S9 OR S10	34,961
S10	TI "Heart Failure" OR AB "Heart Failure"	44,368
S9	TI "Myocardial Decompensation" OR AB "Myocardial Decompensation"	7
S8	TI "Myocardial Failure" OR AB "Myocardial Failure"	44
S7	TI "Cardiac Decompensation" OR AB "Cardiac Decompensation"	98
S6	(TI "Heart Decompensation" OR AB "Heart Decompensation") OR (TI "Heart Decompensated" OR "Heart Decompensated")	8
S5	TI "Cardiac Failure" OR AB "Cardiac Failure"	1,227
S4	(MH "Heart Failure+")	36,787
S3	S1 OR S2	6,601
S2	TI Bundles OR AB Bundles	6,463
S1	TI Bundle OR AB Bundle	6,572

Cochrane Library (Wiley) 24-09-2019

ID Search Hits
#1 MeSH descriptor: [Patient Care Bundles] explode all trees 21
#2 Bundle:ti,ab,kw 1731
#3 Bundles:ti,ab,kw 354
#4 #1 OR #2 OR #3 1968
#5 MeSH descriptor: [Heart Failure] explode all trees 8355
#6 (Cardiac NEAR/1 Failure):ti,ab,kw 993
#7 (Heart NEAR/1 Decompesat*):ti,ab,kw 1
#8 (Cardiac NEAR/1 Decompesat*):ti,ab,kw 0
#9 (Myocardial NEAR/1 Failure):ti,ab,kw 118
#10 (Myocardial NEAR/1 Decompensat*):ti,ab,kw 1
#11 (Heart NEAR/1 Failure):ti,ab,kw 27302
#12 #5 OR #6 OR #7 OR #8 OR #9 OR #10 OR #11 27757
#13 #4 AND #12 338
#14 #13 NOT Branch:ti,ab,kw 32

Global Health (OVID) 26-09-2019

☐	# ▲	Searches	Results
☐	1	Bundle.ti,ab.	1822
☐	2	Bundles.ti,ab.	1467
☐	3	1 or 2	3054
☐	4	Cardiac Failure.ti,ab.	1058

For peer review only

☐	5	Heart Decompensat*.ti,ab.	1
☐	6	Cardiac Decompensat*.ti,ab.	38
☐	7	Myocardial Failure.ti,ab.	61
☐	8	Myocardial Decompensat*.ti,ab.	0
☐	9	Heart Failure.ti,ab.	9089
☐	10	or/4-9	10064
☐	11	3 and 10	66
☐	12	11 not Branch.ti,ab.	17

For peer review only

Supplemental Annex 2. Flowcharts

Flow chart for literature review of HF interventions

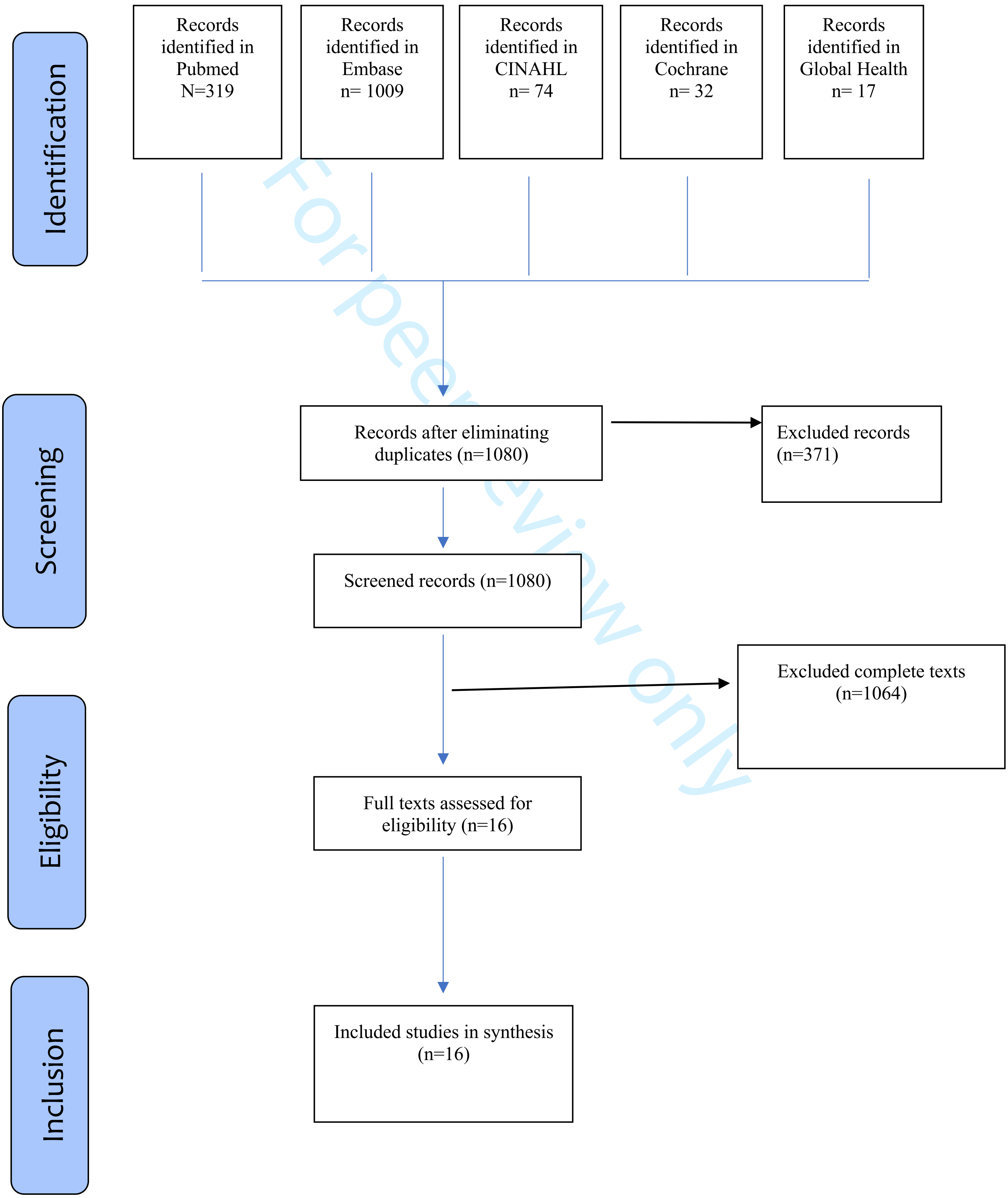

For peer review only

Flow chart for literature review of Readmission

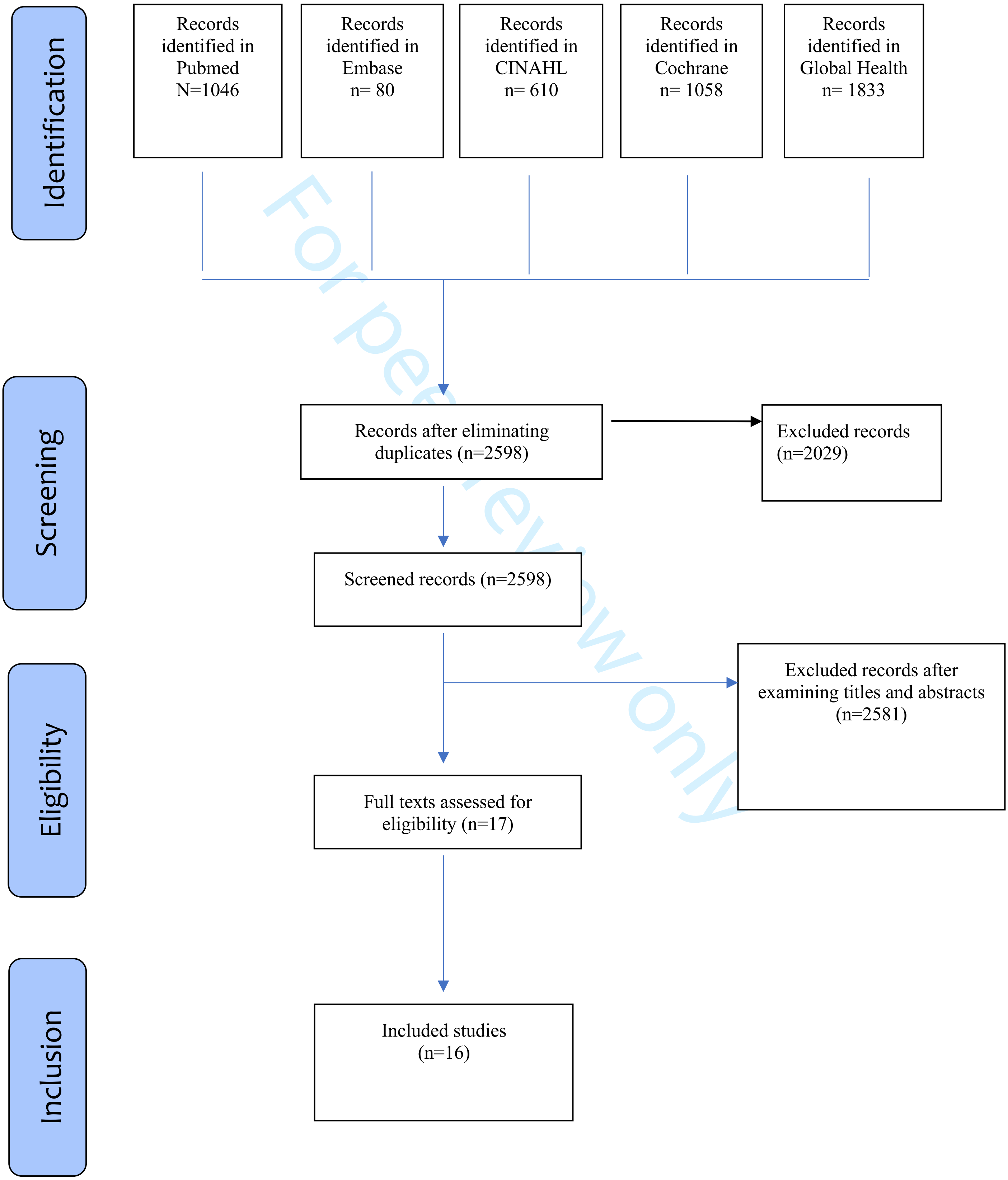

For peer review only
